# Supplementary material for: Is a definitive trial of prehospital continuous positive airway pressure versus standard oxygen therapy for acute respiratory failure indicated? The ACUTE pilot randomised controlled trial
Source: BMJ Open. 2020 Jul 23;10(7):e035915. doi: 10.1136/bmjopen-2019-035915 (PMC7380855; doi:10.1136/bmjopen-2019-035915)
Supplement: Supplementary data [file bmjopen-2019-035915supp001.pdf]

ACUTE pilot trial post-discharge healthcare resource use

Table showing post-discharge healthcare resource use up to 30 days after enrolment

		CPAP<br>N = 42	Standard Oxygen Therapy<br>N = 35	Total<br>N = 77
Discharged after index admission	n=	36	30	66
		22 (61.1%)	22 (73.3%)	44 (66.7%)
Readmission for any reason	n=	22	22	44
		5 (22.7%)	6 (27.3%)	11 (25.0%)
Healthcare provider consulted	n=	23	18	41
		14 (60.9%)	14 (77.8%)	28 (68.3%)
Healthcare provider seen	GP	7 (50.0%)	8 (57.1%)	15 (53.6%)
	Practice or district nurse	7 (50.0%)	5 (35.7%)	12 (42.9%)
	Emergency Department	4 (28.6%)	3 (21.4%)	7 (25.0%)
	Physiotherapist (out of hospital)	2 (14.3%)	3 (21.4%)	5 (17.9%)
	Other*	3 (21.4%)	7 (50.0%)	10 (35.7%)

*Other healthcare providers seen were: Oxygen supplier, care workers, community respiratory team, dentist, heart failure nurse, occupational therapy, outpatient appointment, outpatient x-ray, pharmacist, social worker. Some participants saw multiple other healthcare providers.
