# Supplementary material for: Is a definitive trial of prehospital continuous positive airway pressure versus standard oxygen therapy for acute respiratory failure indicated? The ACUTE pilot randomised controlled trial
Source: BMJ Open. 2020 Jul 23;10(7):e035915. doi: 10.1136/bmjopen-2019-035915 (PMC7380855; doi:10.1136/bmjopen-2019-035915)
Supplement: Supplementary data [file bmjopen-2019-035915supp002.pdf]

BMJ Publishing Group Limited (BMJ) disclaims all liability and responsibility arising from any reliance placed on this supplemental material which has been supplied by the author(s)

ACUTE pilot trial safety reporting procedures and adverse events

Flow chart detailing safety reporting procedures for the ACUTE study

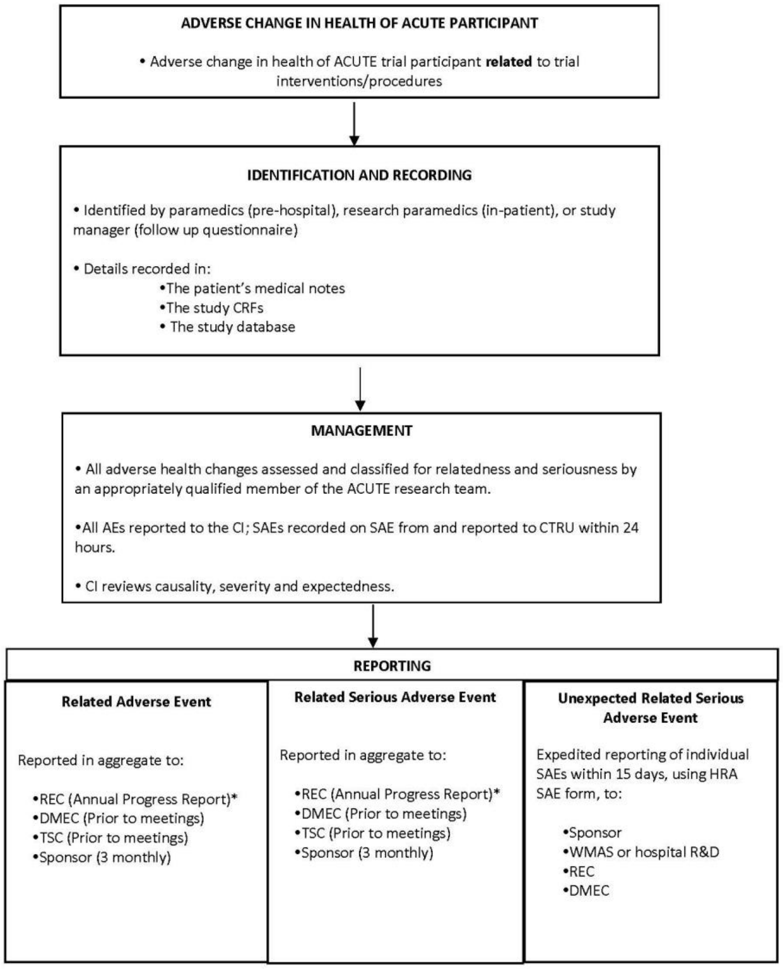

*Related AEs/SAEs resulting in treatment failure

Table of classification of adverse events.

Adverse Health Change Terminology*	Definition
**Seriousness**	
**Adverse Event (AE)**	An adverse change in health that occurs while a patient is taking part in a study
**Serious Adverse Event (SAE)**	Any adverse event occurring while a patient is taking part in a study, that results in:<br><br>• Death<br>• Life Threatening Illness<br>• Hospitalisation<br>• Prolongation of Hospitalisation<br>• Disability or Incapacity<br>• Congenital abnormality or birth defect<br>• Other adverse health change resulting in inability to perform routine activities or significant medical event
**Causality**	
**Unrelated**	An adverse change in health that occurs while a patient is taking part in a study which is not caused by or related to trial treatments.
**Related**	An adverse change in health that occurs while a patient is taking part in a study, which is caused by or related to trial treatments. An AE or SAE is considered related if the relationship between the event and trial treatments is:<br><br>• Possible - There is some evidence to suggest a causal relationship. However, the influence of other factors may have contributed to the event (e.g. the participant's clinical condition, other concomitant treatments).<br>• Probable - There is evidence to suggest a causal relationship and the influence of other factors is unlikely, or<br>• Definite - There is clear evidence to suggest a causal relationship and other possible contributing factors can be ruled out.
**Expectedness**	
**Unexpected**	Any adverse health change that is **NOT** consistent with the known and expected adverse events of trial treatments i.e. it is not listed in the protocol or related documents/literature as an expected occurrence.
**Expected**	Any adverse health change that **IS** consistent with the known and expected adverse events of trial treatments
**Severity***	
**Mild**	An adverse health change that does not interfere with routine activities
**Moderate**	An adverse health change that interferes with routine activities
**Severe**	An adverse health change that makes it impossible to perform routine activities

*The term 'severity', is used to describe the intensity, and should not be confused with 'serious' which is based on participant/event outcome or action criteria. For example, a headache may be severe but not serious, while a minor stroke is serious but may not be severe.

Table summary of the number of AEs and SAEs as well as the proportion of patients experiencing at least one AE or SAE for the ITT population

	CPAP	Standard Oxygen Therapy	Total
Patients	42	35	77
Number of AEs	27	20	47
Number of individuals with an AE	23 (54.8%)	17 (48.6%)	40 (51.9%)
Number of SAEs	22	17	39
Number of individuals with a SAE	19 (45.2%)	15 (42.9%)	34 (44.2%)

ACUTE pilot trial adverse events

Site	AE summary	AE category	Start date	End date	Serious?	Expected SAE?
Stoke	Headache	Other	19/08/2017	19/08/2017	No	No
Stoke	Arrested prior to receiving intervention	Cardiorespiratory arrest	13/09/2017	13/09/2017	Yes	No
Lichfield	Respiratory arrest	Other	17/09/2017	17/09/2017	Yes	No
Lichfield	Died prior to approach	Cardiorespiratory arrest	18/09/2017	18/09/2017	Yes	No
Stoke	Fractured NOF	Other	18/10/2017		Yes	No
Erdington	Social Care readmission	Other	23/10/2017	08/12/2017	Yes	No
Lichfield	CVA	Other	28/10/2017	15/12/2017	Yes	No
Stafford	Death	Cardiorespiratory arrest	29/10/2017	29/10/2017	Yes	Yes
Stafford	Death	Cardiorespiratory arrest	29/10/2017	29/10/2017	Yes	Yes
Stoke	Pt died	Cardiorespiratory arrest	16/11/2017	16/11/2017	Yes	No
Stoke	Re-admission within 30 days	Other	30/11/2017	30/11/2017	No	Yes
Stoke	Claustrophobia	Claustrophobia	12/12/2017	12/12/2017	No	Yes
Erdington	Distress at mask	Other	15/12/2017	15/12/2017	No	Yes

Erdington	Died within 30 days	Cardiorespiratory arrest	21/12/2017	21/12/2017	Yes	No
Stafford	Pt died	Cardiorespiratory arrest	29/12/2017	29/12/2017	Yes	Yes
Stoke	Mild claustrophobia	Claustrophobia	05/01/2018	05/01/2018	No	Yes
Erdington	Readmission to respiratory ward with pneumonia	Other	05/01/2018	15/01/2018	Yes	Yes
Stafford	Re-admission	Other	10/01/2018		Yes	No
Stoke	Death	Cardiorespiratory arrest	14/01/2018	14/01/2018	Yes	Yes
Stoke	Pt died	Cardiorespiratory arrest	17/01/2018	17/01/2018	Yes	Yes
Stafford	Pt died on day of recruitment	Cardiorespiratory arrest	23/01/2018	23/01/2018	Yes	Yes
Stafford	Died ahead of 30 day follow up	Cardiorespiratory arrest	27/01/2018	27/01/2018	Yes	Yes
Stafford	Died on elderly care ward	Cardiorespiratory arrest	28/01/2018	28/01/2018	Yes	Yes
Stoke	Agitation and distress with mask	Progressive respiratory distress	28/01/2018	28/01/2018	No	Yes
Erdington	Death	Cardiorespiratory arrest	06/02/2018	06/02/2018	Yes	Yes
Stafford	Patient died	Cardiorespiratory arrest	20/02/2018	20/02/2018	Yes	Yes
Lichfield	Died prior to 30 day	Cardiorespiratory arrest	03/03/2018	03/03/2018	Yes	Yes
Stafford	Pt re-admitted to hospital during 30-day period after recruitment	Other	08/03/2018	15/03/2018	Yes	Yes
Lichfield	Died prior to approach	Cardiorespiratory arrest	09/03/2018	09/03/2018	Yes	Yes
Erdington	Died following discharge prior to approach	Cardiorespiratory arrest	10/03/2018	10/03/2018	Yes	Yes
Lichfield	Died prior to approach	Cardiorespiratory arrest	17/03/2018	17/03/2018	Yes	Yes

Stoke	Pneumothorax	Tension pneumothorax	28/03/2018	28/03/2018	Yes	Yes
Stoke	Patient got worse	Vomiting	28/03/2018	28/03/2018	No	Yes
Stafford	Pt re-admitted, then died	Other	12/04/2018	15/04/2018	Yes	Yes
Lichfield	Mask wouldn't inflate	Other	17/04/2018	17/04/2018	No	No
Lichfield	Cardiac arrest	Cardiorespiratory arrest	08/05/2018	08/05/2018	Yes	Yes
Lichfield	Death	Cardiorespiratory arrest	31/05/2018	31/05/2018	Yes	Yes
Stafford	Died prior to approach	Cardiorespiratory arrest	03/06/2018	03/06/2018	Yes	Yes
Lichfield	Readmissions within 30 days	Other	30/06/2018	01/07/2018	Yes	Yes
Lichfield	Readmission within 30 days	Other	02/07/2018	06/07/2018	Yes	Yes
Lichfield	Claustrophobia and Progressive Respiratory Distress	Other	05/07/2018	05/07/2018	No	Yes
Lichfield	Pneumothorax	Other	05/07/2018	27/07/2018	Yes	Yes
Stafford	Re-admission	Other	06/07/2018		Yes	Yes
Lichfield	Readmission	Other	07/07/2018	27/07/2018	Yes	Yes
Stoke	Pt re-admitted during 30-day reference period	Other	25/07/2018	03/08/2018	Yes	Yes
Lichfield	Readmission	Other	28/07/2018		Yes	Yes
Stoke	Death	Cardiorespiratory arrest	22/08/2018	22/08/2018	Yes	Yes
