# Supplementary material for: Is a definitive trial of prehospital continuous positive airway pressure versus standard oxygen therapy for acute respiratory failure indicated? The ACUTE pilot randomised controlled trial
Source: BMJ Open. 2020 Jul 23;10(7):e035915. doi: 10.1136/bmjopen-2019-035915 (PMC7380855; doi:10.1136/bmjopen-2019-035915)
Supplement: Author's manuscript [file bmjopen-2019-035915.draft_revisions.pdf]

BMJ Open

BMJ Open is committed to open peer review. As part of this commitment we make the peer review history of every article we publish publicly available.

When an article is published we post the peer reviewers' comments and the authors' responses online. We also post the versions of the paper that were used during peer review. These are the versions that the peer review comments apply to.

The versions of the paper that follow are the versions that were submitted during the peer review process. They are not the versions of record or the final published versions. They should not be cited or distributed as the published version of this manuscript.

BMJ Open is an open access journal and the full, final, typeset and author-corrected version of record of the manuscript is available on our site with no access controls, subscription charges or pay-per-view fees (http://bmjopen.bmj.com).

If you have any questions on BMJ Open's open peer review process please email info.bmjopen@bmj.com

BMJ Open

Prehospital CPAP for Acute Respiratory Failure: the ACUTE pilot randomised controlled trial

Journal:	*BMJ Open*
Manuscript ID	bmjopen-2019-035915
Article Type:	Original research
Date Submitted by the Author:	22-Nov-2019

Complete List of Authors:	Fuller, Gordon; The University of Sheffield, School of Health and Related Research<br>Keating, Sam; The University of Sheffield, School of Health and Related Research<br>Goodacre, Steve; University of Sheffield, School of Health and Related Research<br>Herbert, Esther; The University of Sheffield, School of Health and Related Research<br>Perkins, Gavin; University of Warwick, Clinical Trials Unit<br>Rosser, Andy; West Midlands Ambulance Service NHS Foundation Trust<br>Gunson, Imogen; West Midlands Ambulance Service NHS Foundation Trust<br>Miller, Josh; West Midlands Ambulance Service NHS Foundation Trust<br>ward, matthew; West Midlands Ambulance Service NHS Foundation Trust<br>Bradburn, Mike; University of Sheffield, School of Health and Related Research<br>Thokala, Praveen; The University of Sheffield, School of Health and Related Research<br>Harris, Tim; Bart's Health/NHS, Emergency Medicine<br>Marsh, Maggie; Sheffield Emergency Care Forum<br>Scott, Alex; The University of Sheffield, School of Health and Related Research<br>Cooper, Cindy; University of Sheffield, Clinical Trials Research Unit

Keywords:	Adult thoracic medicine < THORACIC MEDICINE, Adult intensive & critical care < INTENSIVE & CRITICAL CARE, ACCIDENT & EMERGENCY MEDICINE

SCHOLARONE™ Manuscripts

I, the Submitting Author has the right to grant and does grant on behalf of all authors of the Work (as defined in the below author licence), an exclusive licence and/or a non-exclusive licence for contributions from authors who are: i) UK Crown employees; ii) where BMJ has agreed a CC-BY licence shall apply, and/or iii) in accordance with the terms applicable for US Federal Government officers or employees acting as part of their official duties; on a worldwide, perpetual, irrevocable, royalty-free basis to BMJ Publishing Group Ltd ("BMJ") its licensees and where the relevant Journal is co-owned by BMJ to the co-owners of the Journal, to publish the Work in this journal and any other BMJ products and to exploit all rights, as set out in our licence.

The Submitting Author accepts and understands that any supply made under these terms is made by BMJ to the Submitting Author unless you are acting as an employee on behalf of your employer or a postgraduate student of an affiliated institution which is paying any applicable article publishing charge ("APC") for Open Access articles. Where the Submitting Author wishes to make the Work available on an Open Access basis (and intends to pay the relevant APC), the terms of reuse of such Open Access shall be governed by a Creative Commons licence – details of these licences and which Creative Commons licence will apply to this Work are set out in our licence referred to above.

Other than as permitted in any relevant BMJ Author's Self Archiving Policies, I confirm this Work has not been accepted for publication elsewhere, is not being considered for publication elsewhere and does not duplicate material already published. I confirm all authors consent to publication of this Work and authorise the granting of this licence.

For peer review only

Prehospital CPAP for Acute Respiratory Failure: the ACUTE pilot randomised controlled trial

Dr Gordon W Fuller[1]*, Dr Samuel Keating[2], Professor Steve Goodacre[1], Ms Esther Herbert[2], Professor Gavin D Perkins[3], Mr Andy Rosser[4], Ms Imogen Gunson[4], Mr Joshua Miller[4], Mr Matthew Ward[4], Mr Mike Bradburn[2], Dr Praveen Thokala[5], Professor Tim Harris[6], Ms Maggie Marsh[7], Dr Alex Scott[2], Professor Cindy Cooper[2]

Corresponding author*: Dr Gordon Ward Fuller

Work address: Centre for Urgent and Emergency Care Research, School of Health and Related Research, University of Sheffield, Regent Court, 30 Regent Street, Sheffield, S1 4DA, UK

Telephone: +44 (0) 7968 280641

E-mail: g.fuller@sheffield.ac.uk

1. Centre for Urgent and Emergency Care Research, School of Health and Related Research, University of Sheffield, Regent Court, 30 Regent Street, Sheffield, S1 4DA, UK

2. Clinical Trials and Research Unit, School of Health and Related Research, University of Sheffield, Regent Court, 30 Regent Street, Sheffield, S1 4DA, UK

3. Warwick Clinical Trials Unit, University of Warwick, Coventry, CV4 7AL, UK

4. West Midlands Ambulance Service, Trust Headquarters, Millennium Point, Waterfront Business Park, Waterfront Way, Brierley Hill, West Midlands, DY5 1LX, UK

5. Health Economics and Decision Science, School of Health and Related Research, University of Sheffield, Regent Court, 30 Regent Street, Sheffield, S1 4DA, UK

6. School of Medicine and Dentistry, Blizard Institute, Barts and The London School of Medicine and Dentistry, 4 Newark Street, London, E1 2AT, UK

For peer review only

7. Sheffield Emergency Care Forum, Clinical Research Office Sheffield, Royal Hallamshire Hospital, D Floor, Glossop Road, Sheffield, S10 2JF

Word count (excluding title page, abstract, references, figures and tables): 3,528 words

Key words: Continuous positive airway pressure (CPAP), acute respiratory failure, emergency medical services, pilot studies, randomised controlled trial

Contributions of authors:

The following drafted the manuscript: Gordon Fuller (Chief Investigator), Steve Goodacre (Co-chief Investigator), Sam Keating (Trial Manager), Esther Herbert (Statistician), Mike Bradburn (Co-applicant, statistician), Praveen Thokala (Co-applicant, health economist), Josh Miller (Research paramedic), Maggie Marsh (Co-applicant, PPI representative).

The following conceived of, or designed, the work: Gordon Fuller (Chief Investigator), Steve Goodacre (Co-chief Investigator), Sam Keating (Trial Manager), Praveen Thokala (Co-applicant, health economist), Maggie Marsh (PPI representative), Mike Bradburn (Co-applicant, statistician), Professor Gavin D Perkins (Co-applicant), Mr Matthew Ward (Co-applicant), Professor Tim Harris (Co-applicant), Ms Maggie Marsh (Co-applicant, PPI representative), Mr Alex Scott (Qualitative researcher), Professor Cindy Cooper Co-applicant (CTRU Director).

The following were involved in the acquisition of data for the work: Andy Rosser (Lead Research Paramedic), Matthew Ward (Co-applicant), Imogen Gunson (Research paramedic), Josh Miller (Research paramedic)

The following were involved in the analysis and the interpretation of data for the work: Gordon Fuller (Chief Investigator), Steve Goodacre (Co-chief Investigator), Sam Keating (Trial Manager), Esther Herbert (Statistician), Mike Bradburn (Co-applicant, statistician), Praveen Thokala (Co-applicant, health economist), Josh Miller (Research paramedic), Imogen Gunson (Research paramedic), Maggie Marsh (Co-applicant, PPI representative), Mr Alex Scott (Qualitative researcher), Thomas McKenzie (Medical student)

The following contributed to the management and conduct of the trial: Sam Keating (Trial Manager), Sarah Gonzalez (Trial support officer), Andy Rosser (Lead Research Paramedic), Matthew Ward (Co-applicant)

For peer review only

All authors revised the work critically for important intellectual content and were involved in the final approval of the version to be published.

All authors agree to be accountable for all aspects of the work in ensuring that questions related to the accuracy or integrity of any part of the work are appropriately investigated and resolved.

Data Sharing Statement:

All data requests should be submitted to the corresponding author for consideration. Access to anonymised data may be granted following review.

Competing interests: Professor Steve Goodacre is Deputy Director of the NIHR HTA Programme, Chair of the NIHR HTA Commissioning Board and member of the NIHR HTA Funding Strategy Group. Professor Gavin Perkins is a NIHR Senior Investigator and member of the Programme Grants for Applied Research Board. Professor Cindy Cooper is a member of the NIHR Clinical Trials Unit Standing Advisory Committee and of the UK Clinical Research Collaboration Registered Clinical Trials Unit Network Executive Group. All authors have completed the unified competing interest form at www.icmje.org/coi_disclosure.pdf (available on request from the corresponding author) and declare (1) no financial support for the submitted work from anyone other than their employer; (2) no financial relationships with commercial entities that might have an interest in the submitted work; (3) no spouses, partners, or children with relationships with commercial entities that might have an interest in the submitted work; and (4) no non-financial interests that may be relevant to the submitted work.

For peer review only

ABSTRACT

Objectives: To assess the feasibility of a large-scale pragmatic trial comparing prehospital continuous positive airway pressure (CPAP) and standard oxygen therapy in acute respiratory failure.

Design: Single centre, open label, individual patient randomised controlled, external pilot trial.

Setting: UK West Midlands Ambulance Service

Participants: Adults with respiratory distress and peripheral oxygen saturations below British Thoracic Society (BTS) target levels.

Interventions: Prehospital CPAP (O-Two system) versus standard oxygen therapy allocated in a 1:1 ratio using simple randomisation.

Primary and secondary outcome measures: Primary objectives were to assess feasibility and acceptability of trial procedures. The main clinical outcome was 30 day mortality.

Results: Between August 2017 and July 2018 77 patients were enrolled (target 120), including 7 cases with a diagnosis where CPAP could be ineffective or harmful. CPAP was fully delivered in 74% (target 75%). There were no major protocol violations/non-compliances. Full data were available for all key outcomes (targets ≥90%). 30 day mortality was 27.3%. Of deceased patients, 14/21 (68%) either did not have a respiratory condition or had ceiling of treatment decisions implemented excluding hospital non-invasive ventilation and critical care.

Conclusions: Recruitment rate was below target and feasibility was not demonstrated. Limited compliance with CPAP, and difficulty in identifying patients who could benefit from CPAP, indicate that prehospital CPAP is unlikely to materially reduce mortality.

Trial registration: ISRCTN12048261

Funding: National Institute for Health Research's Health Technology Assessment Programme (HTA Project: 15/08/40)

For peer review only

Strengths and limitations of this study

- Controversy has surrounded the use of prehospital CPAP in acute respiratory failure.
- This study investigated whether a large-scale definitive randomised controlled trial of prehospital CPAP in acute respiratory failure is justified.
- Pilot trial recruitment rate was below the target rate and feasibility was not demonstrated.
- Limited patient compliance with CPAP, and difficulty in identifying patients with the potential to benefit, indicated that prehospital CPAP is unlikely to materially reduce mortality
- These findings also argue against routine implementation of CPAP into ambulance services; but would not preclude a CPAP service provided by clinicians with extended training to allow selective targeting of treatment to an appropriate subgroup of patients.

For peer review only

BACKGROUND

Acute respiratory failure (ARF) is a common medical emergency caused by cardiac or respiratory diseases, including heart failure, pneumonia, chronic obstructive pulmonary disease (COPD) and asthma.[1] The incidence of ARF has been estimated at 80 cases per 100,000 per year.[1] Although varying according to the underlying cause, the overall risk of death is high, with estimates of 30 day mortality ranging between 12% and 20%.[2] ARF has substantial health services costs, with patients often requiring prolonged hospital stays, ventilatory support and critical care admissions.[3 4] ARF is responsible for over 3 million National Health Service (NHS) bed days and hospital costs of £9.6 million per year in England.[5 6]

Current prehospital management of ARF in the NHS uses a standard management approach of controlled oxygen therapy; supplemented by specific ancillary treatments directed at the underlying disease.[7] Continuous positive airways pressure (CPAP) is widely used in hospital to treat acute respiratory failure from a number of causes,[8] and it has been suggested that it may be more effective if delivered earlier i.e. en route to hospital.[5] The difficulties of prehospital diagnosis mean that prehospital CPAP is likely to be applied generally to all cases of ARF, rather than directed towards those due to a specific cause.

Existing research investigating prehospital CPAP is inconclusive. A recent evidence synthesis suggested that prehospital CPAP may reduce the risk of mortality in ARF compared to standard treatment, but noted that the primary studies were relatively small, heterogeneous, at risk of bias, and were provided by specialist services, so may not be applicable to routine prehospital practice.[5] These findings suggest that although prehospital CPAP is a promising and potentially efficacious therapy, a large pragmatic trial is needed to confirm effectiveness when implemented as part of routine practice across a clinically relevant population with ARF.

Prior to a large pragmatic trial and economic evaluation, it is first necessary to estimate the incidence of eligible patients to determine whether a trial would be feasible and cost-effective. It is also important to determine whether prehospital CPAP can be delivered successfully in the context of the NHS ambulance services. For these reasons a stand-alone feasibility study is necessary to estimate the incidence of eligible patients, test the feasibility and acceptability of potential definitive trial methods, and address important uncertainties, such as patient selection, delivery of the intervention and event rates.

For peer review only

METHODS

Study design, aims and objectives

The ACUTE (Ambulance CPAP: Use, Treatment effect and Economics) study was an open label, individual randomised, pragmatic, parallel group, external pilot trial. It aimed to determine the feasibility of a definitive trial evaluating the effect of prehospital CPAP (intervention arm) compared to standard oxygen therapy (control arm) on 30-day mortality from ARF. The feasibility and effectiveness outcomes are listed in Table 1. The trial was registered prior to commencement (ISRCTN12048261) and a protocol, with pre-planned outcomes and analysis plan, has been previously published.[9]

Setting and study population

Recruitment took place across four hubs covering 1.5 million people in an English ambulance service between August 2017 and July 2018. Adults with respiratory distress and peripheral oxygen saturation below British Thoracic Society (BTS) target levels (88% for patients with COPD, 94% for other conditions) despite supplemental oxygen (titrated low flow oxygen for COPD, or titrated high flow oxygen in other conditions) were eligible.[10] Patients with pre-existing lack of capacity; or with limited potential to benefit from, or contraindications to CPAP, were excluded. Inclusion and exclusion criteria are detailed in Table 2 and were based on clinician judgement at the scene of incident.

For peer review only

Table 1. ACUTE trial objectives and outcomes

Feasibility outcomes	Feasibility targets
1. Rate of eligible patients per 100,000 population per year	•Target 8 per 100,000 per year i.e. 120 across the 1.5 million population of the 4 hubs
2. Proportion recruited and allocated to treatment appropriately	•Proportion recruited in error and classified as major or minor non-compliances (target 0% and ≤10%).
3. Adherence to allocated treatment	•Adherence to the allocation schedule (target ≥90%)<br>•Adherence to treatment in the CPAP arm (target ≥75%)
4. Retention and data completeness up to 30 days	•Retention at 30 days (target ≥90%)<br>•Data completeness (target ≥90%)
**Clinical outcome measures:**	
1. Proportion surviving to 30 days (primary outcome for definitive trial)	
2. Proportion undergoing endotracheal intubation by 30 days	
3. Proportion admitted to critical care at any point up to 30 days	
4. Mean and median length of hospital stay	
5. Change in Visual Analogue Scale (VAS) dyspnoea score from presentation to immediately before ED arrival	
6. Mean change in quality of life, measured with EQ-5D-5L	
7. Key elements of post-discharge health-care resource use up to 30 days	

For peer review only

Table 2. ACUTE trial eligibility criteria

Inclusion criteria

ALL of:

- Adults ≥ 18 years
- Respiratory distress ,
- Hypoxia:
  - COPD: Sats <88% despite low flow oxygen
  - Other conditions: Sats <94% despite high flow oxygen.

Exclusion criteria

ANY of:

1. Hospital CPAP treatment available within 15 minutes of eligibility assessment.
2. Age < 18 years
3. Known to have terminal illness
4. Known pre-existing lack of capacity (confirmed by relatives, carers or documentary evidence, such as Lasting Power of Attorney)
5. Documented not for resuscitation status
6. Acutely incapacitated patients with known valid advanced directive declining non-invasive ventilation or participation in research
7. The patient has an oxygen alert card
8. Anticipated inability to apply CPAP (e.g. facial deformity)
9. Respiratory failure due to chest trauma
10. Contraindication to CPAP (suspected pneumothorax, respiratory arrest, epistaxis, vomiting, hypotension)
11. Previous enrolment in the ACUTE trial
12. Pregnancy
13. Patients unable to communicate with ambulance service clinicians

For peer review only

Randomisation

Enrolled patients were individually allocated to CPAP or standard oxygen therapy in a 1:1 ratio using simple randomisation. The randomisation sequence was computer generated by an independent statistician not directly involved in the conduct of the trial. The allocation schedule was held centrally on a password-protected, access-restricted network drive. The trial statistician did not have access to the randomisation sequence until after data lock.

Participant enrolment and consent

Ambulance service clinicians (paramedics and ambulance technicians) volunteering to participate in the ACUTE study and trained in trial procedures identified potential participants with ARF when attending emergency 999 ambulance calls. In patients judged to have capacity, verbal consent was obtained for participation. Eligible patients lacking mental capacity were enrolled in the trial without consent if determined to be in their best interests. In all cases, a research paramedic reviewed the participant in hospital as soon as possible after enrolment. For patients with mental capacity, the research paramedics sought written informed consent for further data collection and participation in the trial. Where a patient did not have capacity advice was sought from a Personal Consultee for further participation. When a Personal Consultee was unavailable, a Nominated Consultee was approached for a consent waiver.[11 12] In the contingency that consent was declined, prehospital data was retained and anonymised 30 day mortality data collected. If a patient died before approach for written consent, ethical approval allowed for collection of anonymised prehospital, hospital and 30 day mortality data.

Allocation concealment and blinding

CPAP devices and high concentration oxygen therapy masks were packaged in identically appearing, shrink wrapped, tamper proof sealed, trial equipment boxes measuring 170mm x 170mm x 70mm and weighing 0.52-0.54kg. Equipment boxes were assembled, numbered, and checked for indistinguishability in an audited process in accordance with the randomisation sequence at Sheffield Clinical Trials Research Unit by research assistants not directly involved in the conduct of the trial. Boxes were supplied to participating ambulance hubs, and held unordered by number, in a secure designated storage area. Boxes were signed in and out for each shift by participating clinicians. Research paramedics audited the location and condition of all boxes, and adherence to the allocation schedule on a weekly basis. Due to the physical differences between the CPAP device and standard oxygen mask it

For peer review only

was not possible to subsequently blind patients, ambulance service clinicians, hospital clinicians, or outcome assessors to the treatment arms.

Trial treatments

Immediately after enrolment, paramedics opened the trial equipment box and provide treatment according to whether a CPAP device or high concentration oxygen mask was supplied. Patients in the intervention arm were treated with the O-Two unit (O-Two Medical Technologies, Ontario, Canada), a lightweight, portable, open, single use, low flow CPAP system.[13] Patients in the control arm received standard oxygen therapy using nasal cannula, an air entrainment Venturi mask, a simple face mask, or a non-rebreathing reservoir face mask. The choice of flow rate and oxygen delivery device was determined by ambulance service clinicians according to the patient's condition and peripheral oxygen saturation levels.

Treatment in both arms was targeted to BTS guidelines for peripheral oxygen saturations.[10] Target peripheral oxygen saturations were 88-92% for patients with known/suspected COPD and 94-98% for patients with other suspected causes of acute respiratory failure. Ancillary condition-specific treatments were administered in both trial arms according to Joint Royal Colleges Ambulance Liaison Committee (JRCALC) Clinical Practice Guidelines.[7] Subsequent hospital management, including emergency department CPAP or non-invasive ventilation, was at the discretion of the hospital clinician.

Data collection and safety reporting

Electronic patient records and computer aided dispatch data were screened by research paramedics to identify potentially eligible, but unenrolled, patients. Routinely collected prehospital data (e.g. timings, vital signs) were collated from participants' electronic records by research paramedics. Additional trial specific information was collected using a trial form completed by recruiting ambulance service clinicians and contained within each equipment box. At 30 days, research paramedics reviewed the hospital records to collate details of subsequent progress, treatments, and vital status at 30 days. Quality of life and post-discharge resource use was assessed by questionnaire at 30 days following enrolment, either in person if still in hospital; or by telephone or post if discharged. Adverse health changes in participants were defined, monitored, recorded and reported according to UK Health Research Authority guidance.[14] Only adverse events related to trial interventions or procedures were recorded.

For peer review only

Sample size and statistical analyses

The baseline characteristics of enrolled patients are reported descriptively for the whole trial population, and separately per treatment arm. Feasibility outcomes are reported descriptively for the whole trial population, together with their 95% confidence interval. Summary estimates of relative effectiveness outcomes are presented, overall and stratified by treatment arm, without 95% confidence intervals or p-values. An intention to treat analysis was used, using either a full analysis set, or a complete case analysis for endpoints with missing outcome data.[15]

The ACUTE feasibility study aimed to recruit 120 patients over 12 months, the minimum number recommended to estimate binary parameters for the sample size calculation of the full trial.[16] Mortality under standard care was estimated at 12% and for the full trial a 5% absolute reduction was postulated (i.e. to 7%) in the intervention arm.[1 3 4] This sample size would allow estimation of mortality to within a standard error of 2.7% for use in the sample size calculation for any definitive trial; and feasibility outcomes to be estimated with a precision of <5%. Achieving this target would also indicate a recruitment rate sufficient to deliver the sample size for a definitive trial; and an incidence of ARF necessary to deliver a potentially cost-effective prehospital CPAP service.

All analyses were conducted in the R statistical package (R Foundation for Statistical Computing, Vienna, Austria) in accordance with a pre-specified statistical analysis plan and CONSORT principles. Following review of blinded outcome data during trial management group meetings an unexpectedly high overall mortality rate was noted. A post-hoc descriptive analysis of the deceased patients was therefore conducted.

Trial oversight, ethics and governance

The trial received ethical approval from the NHS Leeds East Research Ethics Committee (Reference: 16/YH/0406). The University of Sheffield provided sponsorship and monitoring oversight for the project. An independent Trial Steering Committee and Data Monitoring and Ethics Committee oversaw the safety, conduct and progress of the study. Funding was received from the National Institute for Health Research's Health Technology Assessment Programme (HTA Project: 15/08/40).

Public and Patient Involvement

The public and patients were fully involved in the ACUTE study from conception to dissemination. The research proposal was developed in partnership with a service user co-applicant and was reviewed in

For peer review only

terms of feasibility and relevance by the Sheffield Emergency Care Forum, the Sheffield Cardiovascular Patient Panel, and the Barnsley Patient Advisory Group. A bursary from the Yorkshire and Humber Research Design Service was used to consult with a group of respiratory patients about their views on the research proposal, consent procedures, and how best to involve patients throughout the project. Feedback from these groups led to a number of important changes, for example reducing the number of patient questionnaires. A service user advisory group was enlisted during the study set up period for collaboration throughout the project and helped develop patient facing research materials. A member of Sheffield Emergency Care Forum (MM) was a co-applicant who provided PPI advice on trial matters, attended TMG meetings, supported study management, and contributed to the study report and interpretation of results. Two further lay PPI representatives served separately on the TSC and DMEC. Asthma UK and PPI co-applicant MM helped develop the plain English summary. Findings from the ACUTE study were reported to our partner PPI groups and presented at a University of Sheffield research engagement event.

For peer review only

RESULTS

Recruitment and consent

Over the recruitment period, 364 patients with ARF meeting ACUTE trial eligibility criteria presented from the 1.5 million population of four participating ambulance service hubs. 161 of those patients (44.2%) were attended by ACUTE trained ambulance service clinicians and could potentially have been recruited. Of these 161, 77 (47.8%) were enrolled in the trial. Slightly more participants were randomised to the CPAP intervention arm (42 cases), than to the standard oxygen control arm (35 cases). Consent for further data collection was declined by 9 patients (11.7%) and 17 patients (22.1%) died prior to research paramedic approach for consent. Figure 1 presents the participant flow for the trial.

Baseline characteristics

The trial population was relatively elderly (median age 71 years), predominantly male (62.3%), and severely unwell (median VAS breathlessness score 9/10, median pulse 115 beats/minute, median respiratory rate 34 breaths/minute, and median initial peripheral oxygen saturations of 78.5%). The most common final hospital diagnoses were COPD (n=21/65, 32.3%) and lower respiratory tract infection (LRTI, n=28/65, 43.1%). A minority of cases (n=4/65, 6.2%) had a non-respiratory primary hospital diagnosis comprising abdominal aortic aneurysm, myocardial infarction, sepsis (not further specified), and liver failure (ascites). Pre-randomisation characteristics were similar across trial arms as summarised in Table 3.

For peer review only

[Figure 1 here]

For peer review only

Figure 1. CONSORT diagram of participants' flow through the ACUTE pilot trial

Table 1. Baseline characteristics of ACUTE trial participants by arm

Baseline Variable	Descriptive statistic	CPAP N = 42	Standard Oxygen Therapy N = 35	Total N = 77
Age	Median (IQR)	70.0 (60.5, 76.8)	73 (65.0, 77.0)	71 (62.0, 77.0)
Sex	Male	27 (64.3%)	21 (60.0%)	48 (62.3%)
Hospital ARF diagnosis*	n=	36	30	65
	Asthma	1 (2.8%)	1 (2.9%)	2 (3.0%)
	COPD	10 (27.8%)	11 (36.7%)	21 (31.8%)
	Heart Failure	4 (11.1%)	2 (6.7%)	6 (9.1%)
	LRTI	17 (47.2%)	11 (36.7%)	28 (42.4%)
	PE	1 (2.8%)	0 (0.0%)	1 (1.5%)
	Other	3 (8.3%)	4 (13.3%)	7 (10.8%)
Breathlessness at enrolment (VAS 0-10)	n=	41	35	76
	Median (IQR)	9.0 (8.0, 10.0)	9.0 (8.0, 9.5)	9.0 (8.0, 10.0)
First systolic blood pressure (mmHg)	n=	40	30	70
	Median (IQR)	136.0 (115.2, 150.5)	126.5 (112.0, 152.0)	134.5 (112.2, 152.0)
First Glasgow Coma Score	n=	42	35	77
	Median (IQR)	15.0 (14.0, 15.0)	15 (14.5, 15.0)	15 (14.0, 15.0)
First oxygen saturations (%)**	n=	41	35	76
	Median (IQR)	78 (74.0, 85.0)	82 (75.5, 86.0)	78.5 (74.8, 86.0)
First pulse rate (bpm)	n=	42	33	75
	Median (IQR)	117.0 (105.0, 125.8)	111 (92.0, 121.0)	115 (100.0, 124.0)
First Respiratory rate (breaths/min)	n=	42	35	77
	Median (IQR)	36.0 (30.5, 40.0)	32 (24.0, 40.0)	34 (28.0, 40.0)
Duration between arrival at scene and	Median (IQR)	43.00 (34.00, 49.75)	36.00 (32.50, 46.50)	40.00 (34.00, 49.00)

For peer review only

Baseline Variable	Descriptive statistic	CPAP<br><br>N = 42	Standard Oxygen Therapy<br>N = 35	Total<br><br>N = 77
departure to hospital (mins)				
Duration between leaving the scene and arriving at hospital (mins)	Median (IQR)	13.00 (9.00, 18.75)	15.00 (10.00, 20.50)	13.00 (10.00, 20.00)

*Consent was declined for data collection in 9 cases, clinical records were unavailable in 2 cases, and in 1 case there was no clear underlying diagnosis apparent in the notes.
IQR: Interquartile range; bpm: beats per minute; COPD: Chronic Obstructive Pulmonary Disease; LRTI: Lower Respiratory Tract Infection.

For peer review only

Feasibility outcomes

Feasibility outcomes, compared to the pre-specified target, are summarised in Table 4. The sample size of 77 enrolled patients resulted in a recruitment rate of 5.1 (95% CI 4.1-6.4) per 100,000 population per year; falling short of the feasibility target of 8 per 100,000 population per year (i.e. 120 patients).

All patients were recruited appropriately with no major or minor protocol non-compliances (targets of ≤10% and 0% respectively). There was full adherence to the allocation schedule (feasibility target ≥90%). Treatment with CPAP was attempted in all patients enrolled in the intervention arm. All patients received appropriate standard oxygen management in the control arm, although one patient was enrolled who used their own CPAP machine.

CPAP was fully delivered as planned (i.e. administered until hospital arrival, or discontinued due to patient improvement after successful treatment) in 73.8% (31/42, target 75%) of intervention arm patients. CPAP was commenced in 90.5% of patients (38/42), with two patients refusing to wear the mask, one patient spontaneously improving and a fourth patient having a cardiac arrest prior to commencement. Of patients commencing CPAP, 31 (81.6%) continued with CPAP until they arrived at hospital: six did not tolerate CPAP and the remaining patient was transferred to a standard oxygen non-rebreather mask due to non-improvement.

Full data were available for key outcomes, including all feasibility endpoints and vital status at 30 days, compared to the feasibility targets of ≥90% retention at 30 days and ≥90% data completeness. A small amount (<1%) of prehospital data describing baseline patient characteristics was not available due to missing values. A larger proportion of hospital data was missing secondary to lack of consent for collection (n=9, 11.7%), absence of local research approvals to access hospital data (n=2, 2.6%), or unclear or absent information in the hospital clinical records (differing across variables, ranging from n=0, 0%, for hospital length of stay to n=25, 32.5%, for emergency department management). Of patients alive at 30 days (n=56), 30 day follow up questionnaires examining quality of life and post-discharge health resource use, were fully completed by 40 patients (71.4%).

Table 4. Summary of feasibility results

For peer review only

Feasibility Outcome	Target	Result
Recruitment rate	8 per 100,000 population per year (i.e. 120 patients recruited	• 5.1 (95% CI 4.1-6.4) per 100,000 population per year<br>• 77 enrolled patients.
Major and minor non-compliances	0% and ≤10%	• 0% - major non-compliances<br>• 1.3% - minor non-compliance
Adherence to the allocation schedule	Target ≥90%	•100% adherence to allocation schedule
Adherence to treatment in the CPAP arm	Target ≥75%	• 74% -CPAP fully delivered as planned
Retention at 30 days	Target ≥90%	•100% - follow up for all feasibility endpoints and 30 day mortality
Data completeness	Target ≥90%	Data completeness for outcomes:<br>• 100% - feasibility outcomes<br>• 100% - 30 day mortality<br>• 81% - 30 day intubation<br>• 84% - admission to critical care<br>• 99% - clinician assessed breathlessness<br>• 86% - length of hospital stay<br>• 85% - baseline EQ-5D-5L*<br>• 71% - 30 day EQ-5D-5L*<br>• 73% - 30 day resource use*

*Of alive patients

For peer review only

Effectiveness and safety outcomes

Overall mortality of the study population was higher than expected with 27.3% (n=21/77) patients dying by 30 days. 28.6% (n=12/42) patients died by 30 days in the CPAP arm and 25.7% (n=9/35) in the standard oxygen arm (expected 12%). An unplanned descriptive analysis of the deceased patients was undertaken to explore the circumstances for the unexpectedly high mortality rate. Data were not available for two cases due to lack of research approvals to access data, and two patients died from non-cardiorespiratory conditions not amenable to NIV (ruptured abdominal aneurysm and liver failure). Of the remaining 17 cases, 6 patients (35.3%) received hospital NIV (n=5/17, 29.4%) or mechanical ventilation (n=1, 5.9%). The other 11 patients had hospital ceiling of treatment decisions excluding hospital NIV and critical care management (11/17, 64/7%). Other secondary effectiveness outcomes are summarised in Table 5, and were similar across trial arms.

In total, 39 expected related serious adverse events occurred in 34 patients (45.2% for the CPAP arm, 42.9% for the standard oxygen therapy arm). The majority were deaths (20/39, 51.2%) or re-admission to hospital within 30 days (13/39, 33.3%). There were no unexpected related SAEs. Two patients (1 intervention arm patient not receiving CPAP and 1 control arm patient) were categorised with related expected serious adverse events following diagnosis with pneumothoraces requiring intercostal drainage after hospital admission.

Table 5. Summary of effectiveness outcomes

For peer review only

Effectiveness outcome	CPAP<br>N=42	Standard Oxygen Therapy<br>N=35	Total<br>N=77
30 day mortality	n = 42<br>12<br>(28.6%)	n = 35<br>9<br>(25.7%)	n = 77<br>21<br>(27.3%)
Intubated	n = 33<br>2<br>(6.1%)	n = 29<br>1<br>(3.4%)	n = 62<br>3<br>(4.8%)
Admission to critical care	n = 35<br>4<br>(11.4%)	n = 30<br>2<br>(6.5%)	n = 65<br>6<br>(9.2%)
Median length of stay (days, IQR)	n = 22<br>10.0<br>(6.5, 12)	n = 22<br>7.0<br>(5, 9.8)	n = 44<br>8<br>(5.5, 11.2)
Change in patient reported breathlessness over prehospital interval (VAS, IQR)	n = 18<br>-3<br>(-4, -2)	n = 18<br>-2<br>(-4, -1)	n = 36<br>-2.5<br>(-4, -1)
Change in clinician assessed breathlessness over prehospital interval (VAS, IQR)	n = 41<br>-3<br>(-5, -1)	n = 35<br>-2<br>(-3.5, 1)	n = 76<br>-2<br>(-4, -1)
30 day EQ-5D-5L (IQR)	n = 22<br>0.82<br>(0.58, 0.95)	n = 18<br>0.73<br>(0.43, 0.89)	n = 40<br>0.76<br>(0.48, 0.92)
Median change in EQ-5D-5L (IQR)	n = 22<br>0.09<br>(-0.01, 0.16)	n = 18<br>0.10<br>(-0.06, 0.19)	n = 40<br>0.09<br>(-0.02, 0.18)

IQR: interquartile range; VAS: Visual analogue scale.

DISCUSSION

For peer review only

Summary of results

Over 12 months 77 patients were enrolled, below the recruitment target of 120 participants. CPAP was fully delivered as planned in 74% of intervention arm patients (target 75%). There were no major protocol violations/non-compliances. Full data were available for key outcomes, including all feasibility endpoints and vital status at 30 days. Mortality was higher than expected, with 27.3% of patients dying by 30 days. Of the deceased patients 68% either did not have a respiratory condition or had explicit or implicit ceiling of treatment decisions excluding hospital NIV or critical care. Two patients were diagnosed with a pneumothorax in the emergency department and were reported as expected related serious adverse events.

Interpretation

These findings suggest that pilot study methods could be used for a future definitive trial. Patients were appropriately recruited using a deferred consent model, there were no protocol non-compliances, adherence to the allocation schedule was complete, and full data were available for key outcomes. The ACUTE pilot trial demonstrated a recruitment rate that was below the target rate considered a priori necessary to deliver a definitive pragmatic trial. However, the majority of potentially eligible cases presented to non-trial trained staff and it is therefore possible that recruitment could be improved if a full trial mandated participation of all ambulance service personnel.

External pilot trials are not designed or powered to generate estimates of clinical effect that should be used for decision-making.[17-19] We therefore draw no conclusions from comparisons of outcomes between CPAP and standard care. However, a number of findings from the ACUTE pilot trial can be used to inform a judgement regarding whether it would be plausible for a large trial to detect an effect from CPAP on mortality and therefore whether a definitive trial might be worthwhile.

Firstly, it is apparent that prehospital identification of patients with the potential to benefit from CPAP may be challenging. A small, but significant minority of cases were ultimately diagnosed with conditions where CPAP could not conceivably be beneficial.[8 20] It is also concerning that pneumothoraces requiring intercostal drainage were detected in 2 cases. Although neither patient received prehospital CPAP, the potential for iatrogenic harm if these cases had been allocated to the intervention arm is conspicuous.

For peer review only

Secondly, delivery of CPAP was relatively limited, with only 74% of intervention arm patients continuing treatment to hospital as planned. Although CPAP may be efficacious, the potential to demonstrate effectiveness would be restricted by lack of treatment compliance.

Thirdly, a key rationale for the implementation of prehospital CPAP is that earlier instigation of treatment will improve outcomes over and above the availability of hospital NIV.[5] In the pilot trial, relatively short on-scene times and conveyance times were recorded (median of 40 minutes and 13 minutes respectively). The potential time advantage from prehospital administration of CPAP may be too small to produce meaningful benefit.

Finally, illness severity was much higher than anticipated. The increased overall 30 day mortality risk of 27.3% might initially appear to offer a definitive trial a greater opportunity to detect a clinically relevant survival benefit. However, many enrolled patients had apparent treatment limitation decisions for ward level hospital care only, indicating a study population with a high prevalence of end-stage cardiorespiratory disease, multiple severe comorbidities, or very poor pre-morbid performance status where CPAP treatment might be futile and overly burdensome.

In summary, although the higher than expected mortality rate might suggest increased potential to detect an absolute difference in mortality, the challenges of providing prehospital CPAP, and characteristics of the patients who would benefit from CPAP, suggest limited potential to improve survival. It therefore appears unlikely that a trial powered to detect a plausible effect size could be designed.

Limitations

The purpose of a feasibility study is to determine if a large-scale trial can be performed.[19] Therefore, challenges that might be interpreted as weaknesses when appraising a definitive RCT (such as low rates of recruitment and adherence) actually represent important learning points in the feasibility setting. Modification of eligibility criteria to specify the presence of a primary cardio-respiratory diagnosis as an inclusion criteria, and exclusion of patients with home CPAP machines, could select a more appropriate study population. The use of retrospective case note review for hospital data collection improved the efficiency of data collection, but is associated with well recognised limitations that could have resulted in inaccurate measurement of study endpoints.

For peer review only

Generalisability

Trial eligibility criteria were broad, excluding only patients where CPAP was contraindicated (e.g. vomiting), where entry would likely not be appropriate (e.g. Do not resuscitate status, pre-existing loss of capacity), or where verbal consent was not possible (e.g. language barrier). The clinical findings reported herein should therefore have strong generalisability to patients presenting with ARF in NHS ambulance services. External validity to other settings, with different demographics or prehospital systems, is less clear. Furthermore, a novel CPAP device was used. Although there are considerable advantages to the studied O-Two unit including small size, low cost and simplicity, there may also be limitations compared to other more complex prehospital CPAP systems e.g. FiO2 and CPAP level are jointly determined by the oxygen flow rate. Consequently, efficacy could differ with other methods of delivering prehospital CPAP.

Comparison to previous literature

A series of recent observational studies have also demonstrated that CPAP can be implemented by EMS.[21-25] These studies are consistent with the ACUTE experience of sub-maximal CPAP adherence and difficult prehospital diagnosis, including treatment of patients with pneumothoraces. A recent systematic review identified 10 trials and quasi-randomised studies comparing prehospital NIV (including CPAP) with standard oxygen therapy.[5] Network meta-analysis suggested that prehospital CPAP is an effective treatment for ARF, with evidence that it reduces mortality (odds ratio 0.41; 95% CrI 0.20 to 0.77) and intubation rate (0.32; 95% CrI 0.17 to 0.62) compared to standard care.2 However, some included studies were at risk of selection bias from lack of allocation concealment and information bias, secondary to unblinded outcome assessment. Furthermore, the meta-analysis findings have doubtful external validity to prehospital practice in many settings. Only one trial included undifferentiated respiratory failure patients, and the methods used to deliver prehospital CPAP (physician or paramedics with online physician support) are not routine in many prehospital care systems.[5]

Conclusions

Pilot trial recruitment rate was below the target rate and feasibility was not demonstrated. Limited compliance with CPAP, and a study population including patients who could not benefit from CPAP, suggest that a clinically significant effect size is not plausible. A definitive effectiveness trial of CPAP is

For peer review only

therefore not recommended. These findings also argue against routine implementation of CPAP into many ambulance services; but would not preclude a CPAP service provided by clinicians with extended training (e.g. prehospital physicians) where advanced clinical skills might allow selective targeting of treatment to an appropriate subgroup of patients.

For peer review only

REFERENCES

1. Creagh-Brown B. Respiratory failure. Medicine 2016;**44**(6):342-45.

2. Stefan MS, Shieh MS, Pekow PS, et al. Epidemiology and outcomes of acute respiratory failure in the United States, 2001 to 2009: a national survey. J. Hosp. Med. 2013;**8**(2):76-82.

3. Ray P, Birolleau S, Lefort Y, et al. Acute respiratory failure in the elderly: etiology, emergency diagnosis and prognosis. Crit. Care 2006;**10**(3):R82.

4. Delerme S, Ray P. Acute respiratory failure in the elderly: diagnosis and prognosis. Age Ageing 2008;**37**(3):251-57.

5. Pandor A, Thokala P, Goodacre S, et al. Pre-hospital non-invasive ventilation for acute respiratory failure: a systematic review and cost-effectiveness evaluation. Health Technol. Assess. 2015;**19**(42):v-vi, 1-102.

6. Health Do. Hospital Episode Statistics. Secondary Hospital Episode Statistics 2014. http://www.hesonline.nhs.uk

7. Fisher JD JRCALC, National Ambulance Service Medical Directors, and Association of Ambulance Chief Executives. *UK ambulance services clinical practice guidelines: 2013. Class Professional Publishing.*: Class Professional Publishing, 2013.

8. Non-invasive ventilation in acute respiratory failure. Thorax 2002;**57**(3):192-211.

9. Fuller GW, Goodacre S, Keating S, et al. The ACUTE (Ambulance CPAP: Use, Treatment effect and economics) feasibility study: a pilot randomised controlled trial of prehospital CPAP for acute respiratory failure. Pilot and feasibility studies 2018;**4**:86-86.

10. O'Driscoll BR, Howard LS, Earis J, et al. BTS guideline for oxygen use in adults in healthcare and emergency settings. Thorax 2017;**72**(Suppl 1):ii1-ii90.

For peer review only

11. Alonzi A, Pringle M. *Mental Capacity Act 2005*, 2007.

12. Britan GoG. Mental Capacity Act 2005. London, 2005.

13. O_two CPAP unit. Secondary O_two CPAP unit 2018. http://otwo.com/emergency-cpap/o_two-single-use-cpap/.

14. Health Do. Health Research Authority Secondary Health Research Authority 2019. https://www.hra.nhs.uk/.

15. Kruse RL, Alper BS, Reust C, et al. Intention-to-treat analysis: who is in? Who is out? J. Fam. Pract. 2002;**51**(11):969-71.

16. Teare MD, Dimairo M, Shephard N, et al. Sample size requirements to estimate key design parameters from external pilot randomised controlled trials: a simulation study. Trials 2014;**15**:264.

17. Arain M, Campbell MJ, Cooper CL, et al. What is a pilot or feasibility study? A review of current practice and editorial policy. BMC Med. Res. Methodol. 2010;**10**:67-67.

18. Leon AC, Davis LL, Kraemer HC. The role and interpretation of pilot studies in clinical research. J. Psychiatr. Res. 2011;**45**(5):626-29.

19. Thabane L, Ma J, Chu R, et al. A tutorial on pilot studies: the what, why and how. BMC Med. Res. Methodol. 2010;**10**:1-1 doi: 10.1186/1471-2288-10-1[published Online First: Epub Date]|.

20. Bello G, De Pascale G, Antonelli M. Noninvasive Ventilation. Clin. Chest Med. 2016;**37**(4):711-21.

21. Hensel M, Strunden MS, Tank S, et al. Prehospital non-invasive ventilation in acute respiratory failure is justified even if the distance to hospital is short. Am. J. Emerg. Med. 2019;**37**(4):651-56.

For peer review only

22. Jacob J, Arranz M, Sancho Ramoneda M, et al. [Noninvasive mechanical ventilation in emergency services in Catalonia: the VNICat registry cohort study]. Emergencias : revista de la Sociedad Espanola de Medicina de Emergencias 2017;**29**(1):33-38.

23. Luiz T, Kumpch M, Gruttner J, et al. Prehospital CPAP Therapy by Emergency Physicians in Patients with Acute Respiratory Failure due to Acute Cardiogenic Pulmonary Edema or Acutely Exacerbated COPD. In Vivo 2016;**30**(2):133-9.

24. Sahu N, Matthews P, Groner K, et al. Observational Study on Safety of Prehospital BLS CPAP in Dyspnea. Prehosp. Disaster Med. 2017;**32**(6):610-14.

25. Willmore A, Dionne R, Maloney J, et al. Effectiveness and safety of a prehospital program of continuous positive airway pressure (CPAP) in an urban setting. Cjem 2015;**17**(6):609-16.

For peer review only

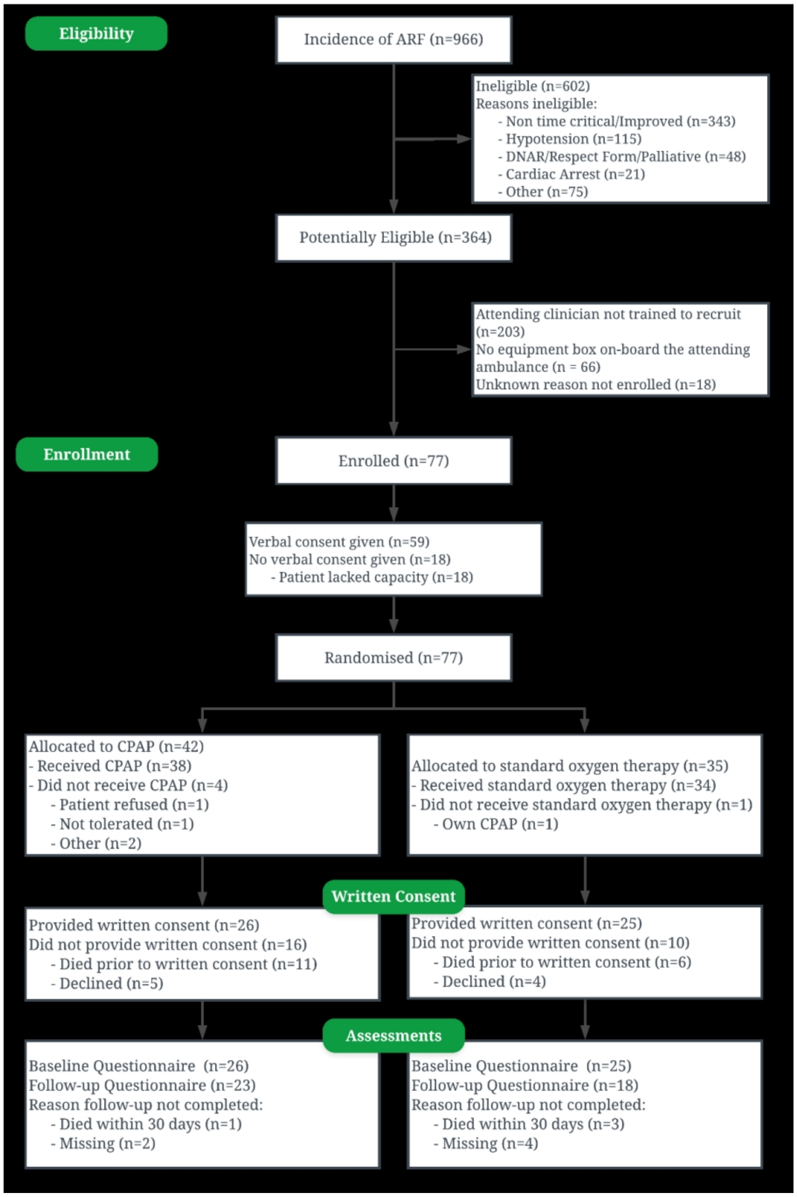
Eligibility
Incidence of ARF (n=966)
Ineligible (n=602)
Reasons ineligible:
- Non time critical/Improved (n=343)
- Hypotension (n=115)
- DNAR/Respect Form/Palliative (n=48)
- Cardiac Arrest (n=21)
- Other (n=75)
Potentially Eligible (n=364)
Attending clinician not trained to recruit (n=203)
No equipment box on-board the attending ambulance (n = 66)
Unknown reason not enrolled (n=18)
Enrollment
Enrolled (n=77)
Verbal consent given (n=59)
No verbal consent given (n=18)
- Patient lacked capacity (n=18)
Randomised (n=77)
Allocated to CPAP (n=42)
- Received CPAP (n=38)
- Did not receive CPAP (n=4)
- Patient refused (n=1)
- Not tolerated (n=1)
- Other (n=2)
Allocated to standard oxygen therapy (n=35)
- Received standard oxygen therapy (n=34)
- Did not receive standard oxygen therapy (n=1)
- Own CPAP (n=1)
Written Consent
Provided written consent (n=26)
Did not provide written consent (n=16)
- Died prior to written consent (n=11)
- Declined (n=5)
Provided written consent (n=25)
Did not provide written consent (n=10)
- Died prior to written consent (n=6)
- Declined (n=4)
Assessments
Baseline Questionnaire (n=26)
Follow-up Questionnaire (n=23)
Reason follow-up not completed:
- Died within 30 days (n=1)
- Missing (n=2)
Baseline Questionnaire (n=25)
Follow-up Questionnaire (n=18)
Reason follow-up not completed:
- Died within 30 days (n=3)
- Missing (n=4)

Fuller *et al. Pilot and Feasibility Studies* (2018) 4:86
https://doi.org/10.1186/s40814-018-0281-9

Pilot and Feasibility Studies

STUDY PROTOCOL Open Access

The ACUTE (Ambulance CPAP: Use, Treatment effect and economics) feasibility study: a pilot randomised controlled trial of prehospital CPAP for acute respiratory failure

Gordon W. Fuller[1*], Steve Goodacre[1], Samuel Keating[2], Gavin Perkins[3], Matthew Ward[4], Andy Rosser[4], Imogen Gunson[4], Joshua Miller[4], Mike Bradburn[2], Praveen Thokala[5], Tim Harris[6], Andrew Carson[4], Maggie Marsh[7] and Cindy Cooper[2]

Abstract

Background: Acute respiratory failure (ARF) is a common and life-threatening medical emergency. Standard prehospital management involves controlled oxygen therapy and disease-specific ancillary treatments. Continuous positive airway pressure (CPAP) is a potentially beneficial alternative treatment that could be delivered by emergency medical services. However, it is uncertain whether this treatment could work effectively in United Kingdom National Health Service (NHS) ambulance services and if it represents value for money.

Methods: An individual patient randomised controlled external pilot trial will be conducted comparing prehospital CPAP to standard oxygen therapy for ARF. Adults presenting to ambulance service clinicians will be eligible if they have respiratory distress with peripheral oxygen saturation below British Thoracic Society (BTS) target levels, despite titrated supplemental oxygen. Enrolled patients will be allocated (1:1 simple randomisation) to prehospital CPAP (O_two system) or standard oxygen therapy using identical sealed boxes. Feasibility outcomes will include incidence of recruited eligible patients, number of erroneously recruited patients and proportion of cases adhering to allocation schedule and treatment, followed up at 30 days and with complete data collection. Effectiveness outcomes will comprise survival at 30 days (definitive trial primary end point), endotracheal intubation, admission to critical care, length of hospital stay, visual analogue scale (VAS) dyspnoea score, EQ-5D-5L and health care resource use at 30 days. The cost-effectiveness of CPAP, and of conducting a definitive trial, will be evaluated by updating an existing economic model. The trial aims to recruit 120 patients over 12 months from four regional ambulance hubs within the West Midlands Ambulance Service (WMAS). This sample size will allow estimation of feasibility outcomes with a precision of < 5%. Feasibility and effectiveness outcomes will be reported descriptively for the whole trial population, and each trial arm, together with their 95% confidence intervals.

(Continued on next page)

* Correspondence: g.fuller@sheffield.ac.uk
[1]Centre for Urgent and Emergency Care Research, School of Health and Related Research, University of Sheffield, Regent Court, 30 Regent Street, Sheffield S1 4DA, UK
Full list of author information is available at the end of the article

© The Author(s). 2018 **Open Access** This article is distributed under the terms of the Creative Commons Attribution 4.0 International License (http://creativecommons.org/licenses/by/4.0/), which permits unrestricted use, distribution, and reproduction in any medium, provided you give appropriate credit to the original author(s) and the source, provide a link to the Creative Commons license, and indicate if changes were made. The Creative Commons Public Domain Dedication waiver (http://creativecommons.org/publicdomain/zero/1.0/) applies to the data made available in this article, unless otherwise stated.

For peer review only - http://bmjopen.bmj.com/site/about/guidelines.xhtml

(Continued from previous page)

Discussion: This study will determine if it is feasible, acceptable and cost-effective to undertake a full-scale trial comparing CPAP and standard oxygen treatment, delivered by ambulance service clinicians for ARF. This will inform NHS practice and prevent inappropriate prehospital CPAP adoption on the basis of limited evidence and at a potentially substantial cost.

Trial registration: ISRCTN12048261. Registered on 30 August 2017. http://www.isrctn.com/ISRCTN12048261

Keywords: Acute respiratory failure, Continuous positive airways pressure, Prehospital, Emergency medical services

Background

Acute respiratory failure (ARF) is a common medical emergency which occurs when disease of the heart or lungs lead to failure to maintain adequate blood oxygen levels and/or increased blood carbon dioxide levels [1]. It is caused by a number of common cardiac or respiratory diseases, including heart failure, pneumonia, and exacerbations of chronic obstructive pulmonary disease (COPD) and asthma [2]. There are approximately 3000 ARF cases in England per year, with a high 14% risk of death within 30 days [3]. ARF has substantial health services costs, with patients often requiring prolonged hospital stays, ventilatory support and critical care admissions [4]. ARF was responsible for over 3 million National Health Service (NHS) bed days in England in 2014 [5].

Current prehospital clinical practice guidelines recommend a standard management approach of oxygen therapy for the treatment of acute respiratory failure, supplemented by specific management options directed at the underlying disease [6–8]. Prehospital administration of continuous positive airway pressure (CPAP) may be a potentially beneficial alternative treatment strategy [9]. CPAP involves delivering oxygen-enriched air to the lungs at increased pressure through a close-fitting face mask and is widely used in hospital to treat ARF from a number of causes [10]. It has been suggested that CPAP may be more effective if delivered earlier, i.e. en route to hospital [11]. The difficulties of prehospital diagnosis mean that prehospital CPAP is likely to be applied generally to all cases of acute respiratory failure, rather than directed towards those due to a specific cause [12].

A recent evidence synthesis reported that prehospital CPAP reduced the risk of mortality and requirement for endotracheal intubation in ARF compared to standard treatment but noted that the primary studies were relatively small, heterogeneous, at risk of bias and may not be applicable to the NHS [3, 9]. A recent economic evaluation suggested that prehospital CPAP was more effective than standard care but was also more expensive, with an incremental cost-effectiveness ratio of £20,514/quality-adjusted life year (QALY) and a 49.5% probability of being cost-effective at the £20,000/QALY threshold. Expected value of perfect information (EVPI) analyses suggested that further research costing up to £22.5 million could represent value for money, while expected value of sample information (EVSI) analyses suggested that a randomised trial recruiting 1000 patients per arm would be cost-effective if research costs were less than £18.1 million. However, these cost-effectiveness results were predicated on the accuracy of published effectiveness data and were very sensitive to estimates for the incidence of acute respiratory failure.

Taken together, these findings suggest that although prehospital CPAP is a promising therapy, further research is needed to examine whether the reported clinical and cost-effectiveness are confirmed in the UK setting. Prior to a large pragmatic trial and economic evaluation comparing prehospital CPAP to standard care, it is first necessary to estimate the incidence of eligible patients and to assess whether a trial would be feasible and cost-effective. We also need to determine whether prehospital CPAP can be delivered in the context of the NHS ambulance service. Prehospital trials need to overcome a number of potential practical barriers if they are to deliver valid data. For these reasons, a stand-alone feasibility study is necessary to estimate the incidence of eligible patients and test the feasibility and acceptability of potential definitive trial methods.

Methods

Study design, aims and objectives

The ACUTE study is a stand-alone, randomised, parallel group, external pilot trial. A concurrent health economic evaluation will also be performed, updating an existing decision analytic model [3]. The study aims to determine the feasibility, acceptability and cost-effectiveness of a definitive trial to evaluate the clinical and cost-effectiveness of prehospital CPAP compared to standard oxygen therapy, for patients attended by ambulance service clinicians with ARF. The study design is summarised in Fig. 1 and the schedule of enrolment, interventions and assessments summarised in Table 1.

The primary objectives are to estimate the following feasibility outcomes:

1. The rate of eligible patients per 100,000 population per year

For peer review only - http://bmjopen.bmj.com/site/about/guidelines.xhtml

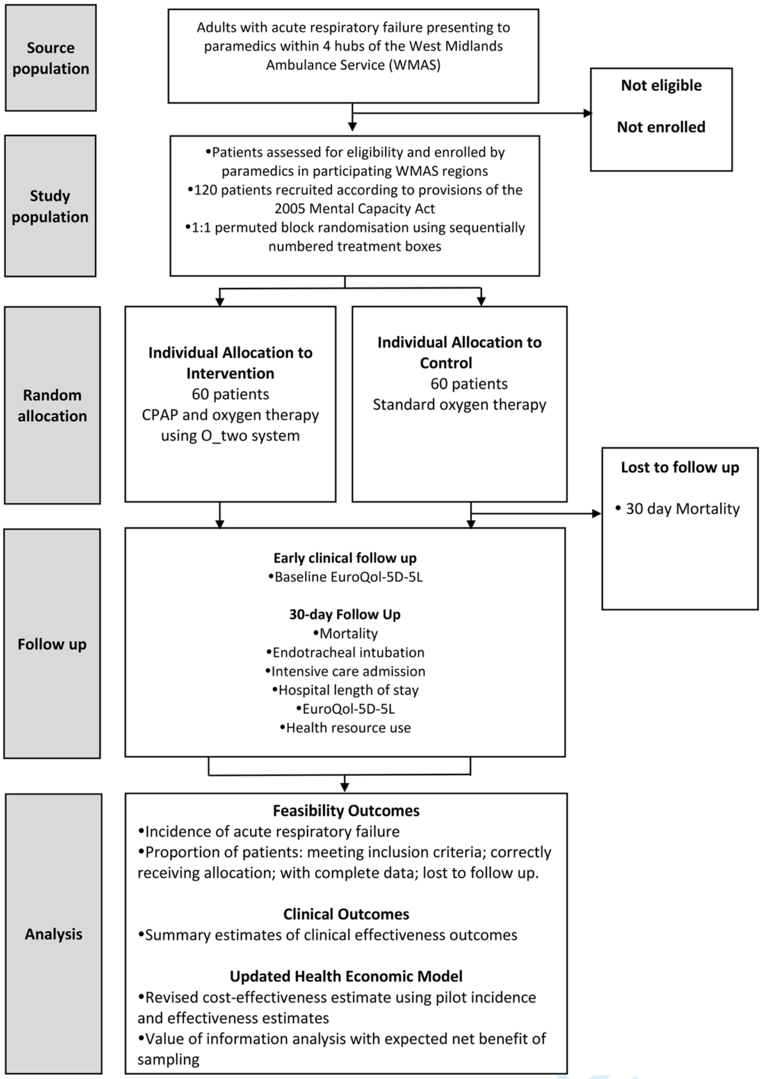

Fig. 1 Flowchart of participants through the ACUTE study [CONSORT diagram]

2. The proportion recruited and allocated to treatment appropriately
3. Adherence to allocated treatment
4. Retention and data completeness up to 30 days
5. Expected net benefit of sampling for a range of study sizes to identify the optimal definitive trial design

Secondary objectives are to estimate the following summary clinical outcome measures, across the whole trial population and per treatment group:

1. Proportion surviving to 30 days
2. Proportion undergoing endotracheal intubation by 30 days
3. Proportion admitted to critical care at any point up to 30 days
4. Mean and median length of hospital stay
5. Change in visual analogue scale (VAS) dyspnoea score from presentation to immediately before ED arrival
6. Mean change in quality of life, measured with EQ-5D-5L
7. Key elements of health care resource use up to 30 days

This clinical data will be used to update an existing network meta-analysis and economic model, [3, 9] to determine the cost-effectiveness of prehospital CPAP given current evidence. The summary clinical outcome measures will also inform the design of any future definitive trial.

Setting and study population

The study will take place in the West Midlands Ambulance Service (WMAS), which serves a mixed urban and rural population of 5.6 million. It employs approximately 4000

For peer review only - http://bmjopen.bmj.com/site/about/guidelines.xhtml

Table 1 Schedule of enrolment, interventions and assessments

	Study period						
	Enrolment	Allocation	Post-allocation				
Time point	0–5 min	5–10 min	10–60 min	1–5 h	1–29 days	30 days	30–37 days
Enrolment							
Eligibility screen	X						
Informed consent	X				X		
Allocation		X					
Interventions							
CPAP			X				
Standard oxygen therapy			X				
Assessments							
Patient characteristics	X	X	X				
ED physiology and treatments				X			
Hospital treatments					X		
Clinical outcomes						X	
Quality of life and health resource use							X

staff across five divisions and operates from 15 'super-hubs', each covering five to ten community ambulance stations. Recruitment will take place across four super-hubs in two divisions covering a population of 1.5 million. Included hubs were chosen to provide a representative mixture of urban, semi-urban and rural localities.

The study population will comprise adults transported to hospital by emergency ambulance with ARF. Inclusion and exclusion criteria will be based on ambulance service clinician judgement at the scene of incident. Acute respiratory failure will be defined as respiratory distress with peripheral oxygen saturation below British Thoracic Society (BTS) target levels (88% for patients with COPD, 94% for other conditions), despite supplemental oxygen (titrated low flow oxygen for COPD, or titrated high flow oxygen in other conditions) [13].

Potential participants will be excluded if any of the following criteria are met:

1. Hospital CPAP treatment available within 15 min of eligibility assessment
2. Age < 18 years
3. Known to have terminal illness
4. Known pre-existing lack of capacity (confirmed by relatives, carers or documentary evidence, such as lasting power of attorney)
5. Documented not for resuscitation status
6. Acutely incapacitated patients with known valid advanced directive declining non-invasive ventilation or participation in research
7. The patient has an oxygen alert card
8. Anticipated inability to apply CPAP (e.g. facial deformity)
9. Respiratory failure due to chest trauma
10. Contraindication to CPAP (suspected pneumothorax, respiratory arrest, epistaxis, vomiting, hypotension)
11. Previous enrolment in the ACUTE trial
12. Pregnancy
13. Patients unable to communicate with ambulance service clinicians

Randomisation, allocation concealment and participant enrolment

Patients presenting to ambulance service clinicians with ARF will be individually randomised to CPAP or standard oxygen therapy in a 1:1 ratio using simple, unrestricted randomisation. The randomisation sequence will be computer-generated by an independent statistician who is not directly involved in the conduct of the trial. The allocation sequence will be held centrally on a password-protected, access-restricted network drive. The trial statistician will not have access to the randomisation sequence until after data lock. Due to the physical differences between the CPAP device and standard oxygen mask, it is not possible to blind patients, ambulance service clinicians or hospital clinicians to the treatment arms. Outcome assessors will also not be blinded. However, both primary and secondary endpoints comprise 'hard' objective measurements, minimising the possibility of information bias.

CPAP devices (intervention arm) and high-concentration oxygen therapy masks (control arm) will be packaged in identical equipment boxes measuring approximately 170 mm × 170 mm × 70 mm. The boxes will be tamper-proof and equal in weight and appearance to maintain

For peer review only - http://bmjopen.bmj.com/site/about/guidelines.xhtml

allocation concealment. Boxes can only be opened after a patient is definitively enrolled in the trial. Once a box is opened when attending a patient, that patient will be included in the study as per allocated treatment. It will not be possible to re-seal the box. Equipment boxes will be assembled, numbered and sealed in accordance with the allocation sequence by researchers not directly involved in the conduct of the trial, under the supervision of the trial manager. Boxes will then be transferred to the central WMAS storage and distribution centre where they will be held in an access-restricted research store. Boxes will subsequently be supplied to participating ambulance hubs as required by the WMAS internal distribution team and held locally in a designated storage area. Ambulance service clinicians will then collect an equipment box at the beginning of each shift. At the end of the shift, boxes will be returned to the ambulance hub equipment store. Boxes will be signed in and out for each shift with ambulance service clinician, ambulance and equipment box details recorded in a distribution log. Research paramedics will monitor the condition, status and location of boxes on a weekly basis to ensure concealment of the allocation sequence until treatment assignment occurs.

Ambulance service clinicians (paramedics, ambulance technicians, paramedic practitioners or critical care paramedics) will identify patients with ARF when attending 999 ambulance calls. Patients meeting trial inclusion criteria will be approached for enrolment in the trial guided by a standardised script. If possible, verbal consent will be obtained for participation prior to enrolment. Patients lacking capacity will be enrolled according to a hierarchical consent process complying with the Mental Capacity Act (MCA) 2005 [14] described below. Immediately after inclusion, ambulance service clinicians will open the trial equipment box and provide treatment according to whether a CPAP device or high-concentration oxygen mask is supplied.

Consent

A hierarchical consent process will be used complying with the Mental Capacity Act (MCA) 2005 [14]. Verbal consent will be obtained for enrolment if the treating ambulance service clinician determines that the patient has capacity. Ambulance service clinicians will enrol patients without consent if the patient does not have capacity. In either case, a research paramedic will review the participant in hospital as soon as possible after enrolment, provide verbal and written information regarding the study and seek written informed consent as soon as the participant has capacity. If the patient does not regain capacity, we will seek advice from a personal consultee for enrolment in the trial [15]. When a personal consultee is unavailable, a nominated consultee will be approached for a consent waiver [15]. If patients (or their consultees) decline consent, all non-identifiable data up to the point of refusal will be retained. No further data collection will be conducted from this point onwards, except for anonymised 30-day mortality data.

Trial treatments

Patients in the intervention arm will be treated by CPAP with supplemental oxygen. Patients in the control arm will receive standard oxygen therapy. Treatment in both arms will be targeted to BTS guidelines for peripheral oxygen saturations [13]. Ancillary condition-specific treatments will be administered in both trial arms according to standard practice guidelines [8]. Ambulance service clinicians (paramedics, ambulance technicians, paramedic practitioners or critical care paramedics) will deliver trial treatments.

Intervention arm

CPAP is a form of non-invasive ventilation (NIV) where oxygen or air is supplied to the upper airways at increased pressure [16]. The ACUTE trial will use the O_two unit, a lightweight, open, single-use, low-flow CPAP system [17]. The device consists of tubing, which is connected to an oxygen source (either a portable oxygen cylinder or the usual ambulance oxygen flow regulator), and an in-line CPAP unit connecting to a close-fitting face mask. The CPAP unit entrains ambient air to increase local mask pressure, providing resistance for the patient to breathe against. The level of CPAP is varied by altering the incoming oxygen flow rate. Thus, the inspired concentration of oxygen varies according to the desired degree of CPAP, as the flow rate is altered. As an open system, with access to ambient air, the device allows unrestricted inspiratory flows and is unaffected by respiratory rate.

Treatment may be commenced at the site of initial clinical contact or after transfer to an ambulance. An appropriately sized mask will be used, with CPAP started at 5 cm H_2O and then incrementally increased by 1 cm H_2O every 2–5 min to a maximum of 15 cm H_2O according to BTS peripheral oxygen saturation targets [13] measured by standard pulse oximetry. Target peripheral oxygen saturations will be 88–92% for patients with known/suspected COPD and 94–98% for patients with other suspected causes of acute respiratory failure. If necessary, nebuliser treatments can be positioned between the face mask and the O_two CPAP unit. CPAP will be continued until arrival at hospital unless not tolerated (e.g. patient request, claustrophobia, anxiety, significant agitation); patient is unable to maintain own airway; decrease in systolic blood pressure to < 90 mmHg; vomiting; epistaxis; conscious level decreases and patient does not respond to voice; patient improvement; or suspected pneumothorax.

For peer review only

Control arm

Oxygen will be delivered at normal atmospheric pressure from a compressed gas tank (or portable oxygen cylinder), via a flow regulator, to the patient using nasal cannula, an air entrainment 'Venturi' mask, a simple face mask or a non-rebreathing reservoir face mask. The exact choice of flow rate and oxygen delivery device will be determined by ambulance service clinicians according to the patient's condition and peripheral oxygen saturation levels. Target oxygen saturations will be 88–92% for patients with known or suspected COPD and 94–98% for other suspected causes of acute respiratory failure.

On arrival at hospital emergency department, staff will be informed of the study and current treatment. Patient care will then be transferred from ambulance service clinicians to hospital clinicians according to normal practice. Care will subsequently continue according to hospital guidelines as implemented by the hospital clinician. In the intervention group, the hospital clinician will determine whether to continue NIV using the O_two unit, switch to an in-hospital system or discontinue NIV altogether. Patients in the control group will be able to receive in-hospital NIV if indicated, according to assessment by the hospital clinician.

The West Midlands Ambulance Service (WMAS), O_two representatives and research paramedics will provide training and support for all ambulance service clinicians based at the participating ambulance hubs. This will involve training in identification of eligible patients, application of the inclusion/exclusion criteria, providing appropriate information and seeking consent, randomisation, delivery of CPAP, monitoring for adverse events (AEs) and data collection. Training will specifically focus on study exclusion criteria, particularly the identification of clinical conditions, e.g. pneumothorax or vomiting, where administration of CPAP could be harmful. Training strategies will include online training modules, small group teaching, demonstration, hands-on familiarisation and scenario-based practice. Only once an ambulance service clinician has received this training and has been assessed to be competent will they be permitted to enrol patients into the trial. Research paramedics will provide ongoing support and training as necessary, including training of new ambulance service clinicians starting at ambulance hubs after recruitment to the study has begun.

Data collection

A recruitment form (case report form (CRF) A), contained within each equipment box, will be completed every time a patient is enrolled in the trial. This will record study number, basic demographic details, eligibility criteria, suspected prehospital diagnosis,, consent details and limited clinical and treatment data not routinely recorded, including a patient and ambulance service clinician completed VAS dyspnoea scale (1–10) recorded on initial assessment and immediately before ED arrival. Routinely collected baseline characteristics, EMS timings, details of treatments provided and vital signs (including peripheral oxygen saturations) en route to hospital will be extracted from the ambulance service patient report forms/electronic patient records into ACUTE case report form B (CRF B) by research paramedics. At 30 days, research paramedics will also review the hospital records to record details of subsequent progress, treatments provided (including time to receiving hospital NIV, if provided), length of hospital stay, use of critical care, any adverse events (AEs) and status at 30 days. Any related AEs or serious AEs will be recorded on the study case report form (CRF B).

Baseline quality of life assessments will be performed by research paramedics shortly after hospital admission, following confirmation of patient consent for participation in the trial. Patients, or their representatives, will be asked to estimate their current health status, using the EQ-5D-5L. This data will be recorded in the study case report form (CRF B). Quality of life and resource use will also be assessed remotely by questionnaire at 30 days following enrolment. Participants will be asked for their preferred method for data collection, either telephone or postal. Initial non-responders will be contacted again after a further 2 weeks by telephone or post. Key elements of health care resource use to be recorded will include hospital services and GP or community services. Participants will also be asked to report any AEs in the 30-day follow-up questionnaire. For patients' declining consent for follow-up, anonymised 30-day mortality will be recorded. The assessments and follow-up for the ACUTE trial are summarised in Table 2.

Data management

All data will be collected and retained in accordance with the UK Data Protection Act 1998 and University of Sheffield clinical trials and research unit (CTRU) standard operating procedures (SOPs). Trial data will be extracted from source documents and CRFs and entered onto a secure data management system. Patient identifiable data (names, date of birth and contact details) will only be collected and entered on the prospect database when written informed consent has been confirmed. Validation reports will be run regularly to check the study data for completeness, accuracy and consistency. Discrepancies will be generated, monitored and managed by research paramedics to resolution.

Sample size and statistical analyses

Participant recruitment and retention will be presented with a CONSORT flow diagram [18]. The following feasibility outcomes will then be reported descriptively

For peer review only

Table 2 Summary of data collection and trial documentation

What	Where	Who	How	When		
				Baseline	Hospital admission	30 days
Consent form						
•Verbal consent	Scene of incident	Paramedics	Verbal	X		
•Written informed consent	Hospital	Research paramedic	Paper		X	
Case report form A						
•Patient demographics	Scene of incident/emergency department	Paramedics	Paper	X		
•Patient characteristics			Telephone	X		
•Prehospital treatments<br>•Adverse events				X<br>X		
Case report form B						
•Patient demographics	Hospital	Research paramedic	Paper		X	
•Baseline quality of life					X	
•Inpatient treatments						X
•30-day mortality						X
•Intubation						X
•Critical care admission						X
•Length of stay<br>•Adverse events					X	X
Patient questionnaire						
•Quality of life	Home	Patient	Paper			X
•Resource use		Research paramedic	Telephone			X
•Adverse events						X
Paramedic questionnaire						
•Acceptability of CPAP	Home/work	Paramedics	Electronic	X		
HRA safety report form						
•Unexpected related serious adverse events	CTRU	Chief investigator	Electronic	X	X	X
SAE form						
•Other serious adverse events	CTRU	Chief investigator	Paper	X	X	X

CTRU clinical trials and research unit, *SAE* serious adverse event, *CPAP* continuous positive airways pressure

for the whole trial population, together with their 95% confidence interval (calculated using the Wilson score method) [19]:

1. Recruitment rate per 100,000 population per year (target 8, i.e. 120 across the 1.5 million population of the 4 WMAS hubs)
2. Proportion recruited in error and classified as minor or major non-compliances (target 0 and ≤ %)
3. Adherence to the allocation schedule (target ≥ 90%)
4. Adherence to treatment in the CPAP arm (target ≥ 75%)
5. Retention at 30 days (target ≥ 90%)
6. Data completeness (target ≥ 90%)

Summary estimates of effectiveness outcomes will also be reported, for the whole trial population and separately per treatment group, with 95% confidence intervals using an as-randomised analysis:

1. Proportion surviving to 30 days
2. Proportion undergoing endotracheal intubation by 30 days
3. Proportion admitted to critical care at any point up to 30 days
4. Mean and median length of hospital stay
5. Change in VAS dyspnoea score from initial presentation versus immediately before ED arrival
6. Mean EQ-5D-5L

For peer review only

7. Key elements of health care resource use up to 30 days

The ACUTE feasibility study aims to recruit $n = 120$ over 12 months. A minimum sample size of 120 was proposed by Teare et al. for pilot studies with dichotomous outcomes, based on the precision to which binary parameters are estimated for use in the sample size calculation of the full trial [20]. Mortality under standard care is estimated at 12%, and for the full trial, a 5% absolute reduction is postulated (i.e. to 7%) in the intervention arm. With $n = 120$, we will therefore be estimated to within a standard error of 2.7% and used in the sample size calculation for the eventual trial. Given the short follow-up period, loss to follow-up of < 5% at 30 days is envisaged. This sample size will allow estimation of feasibility outcomes with a precision of < 5%.

A previous evidence synthesis study produced estimates of the incidence of eligible cases ranging from 3.5 to 40.8 per 100,000 population per year [3, 9]. The lowest estimates were based upon actual patients treated with CPAP, in services with limited ability to deliver treatment for all eligible patients and are likely to be underestimates. The highest estimates were based upon audit data for in-hospital NIV use among emergency admissions and are likely to be overestimates. Assuming that there are 20 eligible cases per 100,000 population per year and 40% are recruited, 120 patients will be recruited from the study's source population of 1.5 million, over 1 year.

Health economics

A previously published meta-analysis and decision analysis model evaluating the cost-effectiveness of prehospital CPAP for ARF will be updated [3, 9, 21]. The decision analysis model simulates the management, outcomes and costs of a hypothetical cohort of patients transported to hospital by emergency ambulance with ARF. Effectiveness is estimated in terms of short-term mortality, using odds ratios from the meta-analysis, and valued as quality-adjusted life years (QALYs) [22]. Costs are estimated from a health service perspective and include all costs related to delivering prehospital CPAP and subsequent treatment of acute respiratory failure. The cost of providing prehospital CPAP is estimated by dividing the total cost of establishing and running the service across an ambulance service by the total number of patients treated.

The ACUTE pilot trial offers an ideal opportunity to estimate the incidence of patients eligible for prehospital CPAP and to update the model with an estimate that is representative and applicable to the NHS. A literature search will also be conducted for new randomised controlled trials comparing prehospital CPAP to standard care; if any are found, the meta-analysis will be updated along with effectiveness data from the ACUTE study. The outputs of the model will be updated estimates of the cost-effectiveness of prehospital CPAP, expressed as the incremental cost per QALY gained by CPAP compared to standard care and the probability of CPAP being cost-effective at £20,000/QALY and £30,000/QALY thresholds for willingness to pay. Expected value of sample information (EVSI) and expected net benefit of sampling (ENBS) for a range of future randomised trial sample sizes will also be calculated. Extensive sensitivity analyses will be performed to explore decision uncertainty including examination of future scenarios where CPAP technology changes in cost or efficacy.

Trial oversight, ethics and governance

The trial has been reviewed and approved by the NHS Leeds East Research Ethics Committee. The University of Sheffield is providing sponsorship and monitoring oversight for the project, and the trial will be conducted in line with the relevant sponsor SOPs. The Sheffield CTRU is responsible for trial management, oversight of data collection, statistical analysis and the health economics analysis. A Trial Management Group (TMG) comprising the applicants and relevant members of the CTRU team will provide ongoing trial support, have responsibility for interpreting the data and writing and reviewing the final report. An independent Trial Steering Committee (TSC) and Data Monitoring and Ethics Committee (DMEC) have been established to oversee the safety, conduct and progress of the study.

Discussion

The ACUTE study will determine the feasibility, acceptability and cost-effectiveness of a definitive trial to evaluate the clinical and cost-effectiveness of prehospital CPAP, compared to standard oxygen therapy, for patients attended by ambulance service clinicians with ARF. Recruitment of at least 120 participants to the pilot trial over 12 months will demonstrate that a definitive trial is feasible and cost-effective. It will also allow estimation of adherence, attrition, data completeness and event rates with sufficient precision to ensure validity of the definitive trial protocol. A sample size of 1518 is projected for the full trial (based on 5% absolute effect size, 88% baseline 30-day survival, 90% power, two-sided significance of 5%, 5% attrition at 30 days).

Important design issues during the development of the ACUTE protocol were the choice of CPAP device, method of randomisation/allocation concealment and consent processes. There is a wide array of possible methods for administering prehospital CPAP [16]. A single-use disposable unit was chosen over a CPAP machine on the basis of cheaper cost, simplicity of use and

For peer review only

portability. The O_two unit offers several advantages over competing devices. It has low oxygen flow rates, allowing prolonged use without depleting ambulance oxygen stores. It may also be used with portable oxygen cylinders, offering the possibility to commence treatment in a patient's home. As an open system, with access to ambient air, the device allows unrestricted inspiratory flows and is unaffected by respiratory rate. A potential disadvantage is that the level of CPAP is varied by altering the incoming oxygen flow rate. Thus, the inspired concentration of oxygen cannot be varied independently from the degree of CPAP. The prehospital time interval, and changes in patient physiology (peripheral oxygen saturations, respiratory rate, and dyspnoea score), will be recorded to evaluate any transport delays or prehospital deterioration following introduction of a new EMS treatment. The O_two CPAP device has also been used in Australian and Canadian emergency medical services with an excellent safety record. A range of healthcare practitioners have successfully provided CPAP in these settings (including entry-level technicians, paramedics and critical care paramedics); however, it is possible that effectiveness could vary according to clinician skill and experience.

Cluster randomisation has been used extensively in previous prehospital trials but is limited by a high risk of post-randomisation selection and other biases [23]. Individual randomisation is preferred to maximise internal validity [24] but is challenged by the time-pressured and confused prehospital environment. Central telephone randomisation has not been found to be feasible, and sequentially numbered envelopes have well known limitations. We therefore implemented randomisation using identical trial equipment boxes, packaged according to the randomisation schedule. Packaging was designed to be identical in weight, feel and appearance across trial arms. Research paramedics will closely monitor equipment boxes during the trial to detect any attempts to subvert the randomisation sequence.

Emergency trials will often need to recruit unwell, incapacitated patients. As the most severe ARF patients are most likely to benefit from CPAP, it is not possible to exclude such patients from the trial. In accordance with the Declaration of Helsinki, UK MCA 2005 and Good Clinical Practice guidelines, a hierarchical consent process was designed [14, 25, 26]. Ambulance service clinicians assess capacity as a core skill and will obtain verbal consent for participation if patients can retain, weigh, use and communicate information. In common with other prehospital trials, patients without capacity can be enrolled without consent. At the earliest opportunity after admission to hospital, formal written informed consent will be confirmed with patients or their consultees. These consent procedures were developed in accordance with best practice and after consultation with patient and public representatives, prior to review and approval by an independent research ethics committee.

Trial status

The current protocol is version 2 (16 February 2017). The current protocol version is available from the study website (www.sheffield.ac.uk/acute). The trial opened to recruitment in August 2017. Recruitment is anticipated to run until 31 July 2018 with trial completion by 31 December 2018. As of December 2017, 25% of the study population was recruited.

Abbreviations

ACUTE: Ambulance CPAP: Use, Treatment effect and economics; AE: Adverse event; ARF: Acute respiratory failure; BTS: British Thoracic Society; CONSORT: Consolidated Standards of Reporting Trials; COPD: Chronic obstructive pulmonary disease; CPAP: Continuous positive airway pressure; CRF: Case report form; CTRU: Clinical trials and research unit; DMEC: Data monitoring committee; ED: Emergency department; EVPI: Estimated value of perfect information; GCP: Good clinical practice; GP: General practitioner; H_2O: Water; HRA: Health Research Authority; HTA: Health technology assessment; ICER: Incremental cost-effectiveness ratio; MCA: Mental Capacity Act; NHS: National Health Service; NIV: Non-invasive ventilation; PI: Principal investigator; QALY: Quality-adjusted life year; ScHARR: School of Health and Related Research; SOP: Standard operating procedure; TMG: Trial Management Group; TSC: Trial Steering Committee; UK: United Kingdom; VAS: Visual analogue scale; WMAS: West Midlands Ambulance Service

Acknowledgements

We thank the Sheffield Clinical Trials and Research Unit staff for their involvement. In addition, we would like to thank all recruiting sites, in particular the principal investigators, co-investigators, research paramedics and WMAS clinicians.

Funding

ACUTE is funded by the National Institute for Health Research's HTA Programme (HTA Project: 15/08/40). The views and opinions expressed therein are those of the authors and do not necessarily reflect those of the HTA, NIHR, NHS or the Department of Health.

Availability of data and materials

The datasets generated and/or analysed during the current study are available from the corresponding author on reasonable request.

Authors' contributions

GF and SG conceived the study. GF, SG, SK, GP, MW, AR, AC, MB, GP, CC, MM and TH participated in its design and coordination. GF and SK drafted the manuscript. MB and EH designed the statistical analysis plan. PT developed the protocol for the health economics analysis. IG and JM provided trial training and will perform data collection. All authors read, revised and approved the final manuscript.

Ethics approval and consent to participate

The ACUTE trial was given approval by the Health Research Authority (HRA) and the NHS Leeds East Research Ethics Committee, for all centres involved in the trial (Reference: 16/YH/0406).

Competing interests

SG is the Chair of the NIHR Health Technology Assessment Clinical Evaluation and Trials Board.

Publisher's Note

Springer Nature remains neutral with regard to jurisdictional claims in published maps and institutional affiliations.

For peer review only

Author details
[1]Centre for Urgent and Emergency Care Research, School of Health and Related Research, University of Sheffield, Regent Court, 30 Regent Street, Sheffield S1 4DA, UK. [2]Clinical Trials and Research Unit, School of Health and Related Research, University of Sheffield, Regent Court, 30 Regent Street, Sheffield S1 4DA, UK. [3]Warwick Clinical Trials Unit, University of Warwick, Coventry CV4 7AL, UK. [4]West Midlands Ambulance Service, Trust Headquarters, Millennium Point, Waterfront Business Park, Waterfront Way, Brierley Hill, West Midlands DY5 1LX, UK. [5]Health Economics and Decision Science, School of Health and Related Research, University of Sheffield, Regent Court, 30 Regent Street, Sheffield S1 4DA, UK. [6]Blizard Institute, Barts and The London School of Medicine and Dentistry, 4 Newark Street, London E1 2AT, UK. [7]Sheffield Emergency Care Forum, Clinical Research Office Sheffield, Royal Hallamshire Hospital, D Floor, Glossop Road, Sheffield S10 2JF, UK.

Received: 23 January 2018 Accepted: 26 April 2018
Published online: 18 June 2018

For peer review only

References

1. Greene KE, Peters JI. Pathophysiology of acute respiratory failure. Clin Chest Med. 1994;15(1):1–12.
2. Chapman GW. Pathophysiology and treatment of acute respiratory failure. J Natl Med Assoc. 1984;76(2):201–6.
3. Pandor A, Thokala P, Goodacre S, Poku E, Stevens JW, Ren S, et al. Pre-hospital non-invasive ventilation for acute respiratory failure: a systematic review and cost-effectiveness evaluation. Health Technol Assess. 2015;19(42): v–vi. 1-102
4. Ray P, Birolleau S, Lefort Y, Becquemin MH, Beigelman C, Isnard R, et al. Acute respiratory failure in the elderly: etiology, emergency diagnosis and prognosis. Crit Care. 2006;10(3):R82.
5. Health and Social Care Information Centre. Hospital Episode Statistics: Admitted patient care - 2014- 15. Available at: https://files.digital.nhs.uk/publicationimport/pub19xxx/pub19124/hosp-epis-stat-admi-summrep-2014-15-rep.pdf . Accessed 3 May 2018.
6. Ponikowski P, Voors AA, Anker SD, Bueno H, Cleland JG, Coats AJ, et al. 2016 ESC Guidelines for the diagnosis and treatment of acute and chronic heart failure: the task force for the diagnosis and treatment of acute and chronic heart failure of the European Society of Cardiology (ESC). Developed with the special contribution of the Heart Failure Association (HFA) of the ESC. Eur J Heart Fail. 2016;18(8):891–975.
7. Excellence NIfHaC. Chronic obstructive pulmonary disease in over 16s: diagnosis and management clinical guideline [CG101]. London: National Institute for Health and Care Excellence; 2010.
8. Joint Royal Colleges Ambulance Liaison Committee. UK Ambulance Services Clinical Practice Guidelines 2013. Bristol: Class Professional Publishing; 2013.
9. Goodacre S, Stevens JW, Pandor A, Poku E, Ren S, Cantrell A, et al. Prehospital noninvasive ventilation for acute respiratory failure: systematic review, network meta-analysis, and individual patient data meta-analysis. Acad Emerg Med Off J Soc Acad Emerg Med. 2014;21(9):960–70.
10. Hess DR. Noninvasive ventilation for acute respiratory failure. Respir Care. 2013;58(6):950–72.
11. Masip J, Roque M, Sanchez B, Fernandez R, Subirana M, Exposito JA. Noninvasive ventilation in acute cardiogenic pulmonary edema: systematic review and meta-analysis. JAMA. 2005;294(24):3124–30.
12. Christie A, Costa-Scorse B, Nicholls M, Jones P, Howie G. Accuracy of working diagnosis by paramedics for patients presenting with dyspnoea. Emerg Med Australas. 2016 Oct;28(5):525–30.
13. O'Driscoll BR, Howard LS, Earis J, Mak V. BTS guideline for oxygen use in adults in healthcare and emergency settings. Thorax. 2017;72 (Suppl 1):ii1–ii90.
14. Johnston C, Liddle J. The Mental Capacity Act 2005: a new framework for healthcare decision making. J Med Ethics. 2007;33(2):94–7.
15. Coats TJ, Ng G, Shakur H. Consent in emergency research. Emerg Med J. 2006;23(6):489–90.
16. Mas A, Masip J. Noninvasive ventilation in acute respiratory failure. Int J Chron Obstruct Pulmon Dis. 2014;9:837–52.
17. O_Two. O-Two Single-Use Open Circuit CPAP Delivery System. Availble at: http://otwo.com/wpcontent/uploads/Disposable-Open-CPAP-System-Rev-6-10_16.pdf. Accessed 3 May 2018.
18. Schulz KF, Altman DG, Moher D. CONSORT 2010 statement: updated guidelines for reporting parallel group randomised trials. BMJ (Clinical research ed). 2010;340:c332.
19. Newcombe RG. Interval estimation for the difference between independent proportions: comparison of eleven methods. Stat Med. 1998;17(8):873–90.
20. Teare MD, Dimairo M, Shephard N, Hayman A, Whitehead A, Walters SJ. Sample size requirements to estimate key design parameters from external pilot randomised controlled trials: a simulation study. Trials. 2014;15:264.
21. Research. NIoH. Guide to the methods of technology appraisal. London: NICE; 2013.
22. Weinstein MC, Torrance G, McGuire A. QALYs: the basics. Value Health. 2009; 12(Suppl 1):S5–9.
23. Hayes RJ, Moulton LH. Cluster randomised trials, vol. xxii. Boca Raton: CRC Press; 2009. p. 315.
24. Akobeng AK. Understanding randomised controlled trials. Arch Dis Child. 2005;90(8):840–4.
25. World Medical Association Declaration of Helsinki: ethical principles for medical research involving human subjects. JAMA. 2013;310(20):2191–4.
26. ICH harmonized tripartite guideline: guideline for good clinical practice. J Postgrad Med. 2001;47(1):45–50.

Ready to submit your research? Choose BMC and benefit from:

- fast, convenient online submission
- thorough peer review by experienced researchers in your field
- rapid publication on acceptance
- support for research data, including large and complex data types
- gold Open Access which fosters wider collaboration and increased citations
- maximum visibility for your research: over 100M website views per year

At BMC, research is always in progress.

Learn more biomedcentral.com/submissions

BMC

CONSORT 2010 checklist of information to include when reporting a pilot or feasibility randomized trial in a journal or conference abstract

Item	Description	Reported on line number
Title	Identification of study as randomised pilot or feasibility trial	Title page
Authors *	Contact details for the corresponding author	Title page
Trial design	Description of pilot trial design (eg, parallel, cluster)	5
Methods		
Participants	Eligibility criteria for participants and the settings where the pilot trial was conducted	7
Interventions	Interventions intended for each group	9
Objective	Specific objectives of the pilot trial	3
Outcome	Prespecified assessment or measurement to address the pilot trial objectives**	11
Randomization	How participants were allocated to interventions	5,9
Blinding (masking)	Whether or not participants, care givers, and those assessing the outcomes were blinded to group assignment	5
Results		
Numbers randomized	Number of participants screened and randomised to each group for the pilot trial objectives**	13
Recruitment	Trial status†	N/A
Numbers analysed	Number of participants analysed in each group for the pilot objectives**	13-18
Outcome	Results for the pilot objectives, including any expressions of uncertainty**	13-18
Harms	Important adverse events or side effects	13-18
Conclusions	General interpretation of the results of pilot trial and their implications for the future definitive trial	19
Trial registration	Registration number for pilot trial and name of trial register	22
Funding	Source of funding for pilot trial	23

Citation: Eldridge SM, Chan CL, Campbell MJ, Bond CM, Hopewell S, Thabane L, et al. CONSORT 2010 statement: extension to randomised pilot and feasibility trials. BMJ. 2016;355.

**this item is specific to conference abstracts*

***Space permitting, list all pilot trial objectives and give the results for each. Otherwise, report those that are a priori agreed as the most important to the decision to proceed with the future definitive RCT.*

†For conference abstracts.

For peer review only

CONSORT 2010 checklist of information to include when reporting a randomised trial*

Section/Topic	Item No	Checklist item	Reported on page No
**Title and abstract**			
	1a	Identification as a randomised trial in the title	P 1
	1b	Structured summary of trial design, methods, results, and conclusions (for specific guidance see CONSORT for abstracts)	P 3
**Introduction**			
Background and objectives	2a	Scientific background and explanation of rationale	P 5
	2b	Specific objectives or hypotheses	P 5
**Methods**			
Trial design	3a	Description of trial design (such as parallel, factorial) including allocation ratio	P 6
	3b	Important changes to methods after trial commencement (such as eligibility criteria), with reasons	P 11
Participants	4a	Eligibility criteria for participants	P 8
	4b	Settings and locations where the data were collected	P 6
Interventions	5	The interventions for each group with sufficient details to allow replication, including how and when they were actually administered	P 10
Outcomes	6a	Completely defined pre-specified primary and secondary outcome measures, including how and when they were assessed	P 7
	6b	Any changes to trial outcomes after the trial commenced, with reasons	NA
Sample size	7a	How sample size was determined	P 11
	7b	When applicable, explanation of any interim analyses and stopping guidelines	NA
Randomisation:			
Sequence generation	8a	Method used to generate the random allocation sequence	P 9
	8b	Type of randomisation; details of any restriction (such as blocking and block size)	P 9
Allocation concealment mechanism	9	Mechanism used to implement the random allocation sequence (such as sequentially numbered containers), describing any steps taken to conceal the sequence until interventions were assigned	P 9
Implementation	10	Who generated the random allocation sequence, who enrolled participants, and who assigned participants to interventions	P 6, 9
Blinding	11a	If done, who was blinded after assignment to interventions (for example, participants, care providers, those	P 9

For peer review only

For peer review only - http://bmjopen.bmj.com/site/about/guidelines.xhtml

		assessing outcomes) and how	
	11b	If relevant, description of the similarity of interventions	NA
Statistical methods	12a	Statistical methods used to compare groups for primary and secondary outcomes	P 11
	12b	Methods for additional analyses, such as subgroup analyses and adjusted analyses	NA
**Results**			
Participant flow (a diagram is strongly recommended)	13a	For each group, the numbers of participants who were randomly assigned, received intended treatment, and were analysed for the primary outcome	P 12, 13
	13b	For each group, losses and exclusions after randomisation, together with reasons	P 12, 13
Recruitment	14a	Dates defining the periods of recruitment and follow-up	P 12
	14b	Why the trial ended or was stopped	NA
Baseline data	15	A table showing baseline demographic and clinical characteristics for each group	P 14
Numbers analysed	16	For each group, number of participants (denominator) included in each analysis and whether the analysis was by original assigned groups	P 16, 18, 19
Outcomes and estimation	17a	For each primary and secondary outcome, results for each group, and the estimated effect size and its precision (such as 95% confidence interval)	NA
	17b	For binary outcomes, presentation of both absolute and relative effect sizes is recommended	NA
Ancillary analyses	18	Results of any other analyses performed, including subgroup analyses and adjusted analyses, distinguishing pre-specified from exploratory	P 18
Harms	19	All important harms or unintended effects in each group (for specific guidance see CONSORT for harms)	P 18
**Discussion**			
Limitations	20	Trial limitations, addressing sources of potential bias, imprecision, and, if relevant, multiplicity of analyses	P 21
Generalisability	21	Generalisability (external validity, applicability) of the trial findings	P 22
Interpretation	22	Interpretation consistent with results, balancing benefits and harms, and considering other relevant evidence	P 20, 21
**Other information**			
Registration	23	Registration number and name of trial registry	P 5
Protocol	24	Where the full trial protocol can be accessed, if available	P 5
Funding	25	Sources of funding and other support (such as supply of drugs), role of funders	P 11

*We strongly recommend reading this statement in conjunction with the CONSORT 2010 Explanation and Elaboration for important clarifications on all the items. If relevant, we also recommend reading CONSORT extensions for cluster randomised trials, non-inferiority and equivalence trials, non-pharmacological treatments, herbal interventions, and pragmatic trials. Additional extensions are forthcoming: for those and for up to date references relevant to this checklist, see www.consort-statement.org.

For peer review only

BMJ Open

Is a Definitive Trial of Prehospital Continuous Positive Airway Pressure versus standard oxygen therapy for Acute Respiratory Failure Indicated? The ACUTE pilot randomised controlled trial

Journal:	*BMJ Open*
Manuscript ID	bmjopen-2019-035915.R1
Article Type:	Original research
Date Submitted by the Author:	23-Apr-2020
Complete List of Authors:	Fuller, Gordon; The University of Sheffield, School of Health and Related Research<br>Keating, Sam; The University of Sheffield, School of Health and Related Research<br>Goodacre, Steve; University of Sheffield, School of Health and Related Research<br>Herbert, Esther; The University of Sheffield, School of Health and Related Research<br>Perkins, Gavin; University of Warwick, Clinical Trials Unit<br>Rosser, Andy; West Midlands Ambulance Service NHS Foundation Trust<br>Gunson, Imogen; West Midlands Ambulance Service NHS Foundation Trust<br>Miller, Josh; West Midlands Ambulance Service NHS Foundation Trust<br>ward, matthew; West Midlands Ambulance Service NHS Foundation Trust<br>Bradburn, Mike; University of Sheffield, School of Health and Related Research<br>Thokala, Praveen; The University of Sheffield, School of Health and Related Research<br>Harris, Tim; Bart's Health/NHS, Emergency Medicine<br>Marsh, Maggie; Sheffield Emergency Care Forum<br>Scott, Alex; The University of Sheffield, School of Health and Related Research<br>Cooper, Cindy; University of Sheffield, Clinical Trials Research Unit
**Primary Subject Heading**:	Emergency medicine
Secondary Subject Heading:	Cardiovascular medicine, Respiratory medicine
Keywords:	Adult thoracic medicine < THORACIC MEDICINE, Adult intensive & critical care < INTENSIVE & CRITICAL CARE, ACCIDENT & EMERGENCY MEDICINE

I, the Submitting Author has the right to grant and does grant on behalf of all authors of the Work (as defined in the below author licence), an exclusive licence and/or a non-exclusive licence for contributions from authors who are: i) UK Crown employees; ii) where BMJ has agreed a CC-BY licence shall apply, and/or iii) in accordance with the terms applicable for US Federal Government officers or employees acting as part of their official duties; on a worldwide, perpetual, irrevocable, royalty-free basis to BMJ Publishing Group Ltd ("BMJ") its licensees and where the relevant Journal is co-owned by BMJ to the co-owners of the Journal, to publish the Work in this journal and any other BMJ products and to exploit all rights, as set out in our licence.

The Submitting Author accepts and understands that any supply made under these terms is made by BMJ to the Submitting Author unless you are acting as an employee on behalf of your employer or a postgraduate student of an affiliated institution which is paying any applicable article publishing charge ("APC") for Open Access articles. Where the Submitting Author wishes to make the Work available on an Open Access basis (and intends to pay the relevant APC), the terms of reuse of such Open Access shall be governed by a Creative Commons licence – details of these licences and which Creative Commons licence will apply to this Work are set out in our licence referred to above.

Other than as permitted in any relevant BMJ Author's Self Archiving Policies, I confirm this Work has not been accepted for publication elsewhere, is not being considered for publication elsewhere and does not duplicate material already published. I confirm all authors consent to publication of this Work and authorise the granting of this licence.

For peer review only

Is a Definitive Trial of Prehospital Continuous Positive Airway Pressure versus standard oxygen therapy for Acute Respiratory Failure Indicated? The ACUTE pilot randomised controlled trial

Dr Gordon W Fuller[1]*, Dr Samuel Keating[2], Professor Steve Goodacre[1], Ms Esther Herbert[2], Professor Gavin D Perkins[3], Mr Andy Rosser[4], Ms Imogen Gunson[4], Mr Joshua Miller[4], Mr Matthew Ward[4], Mr Mike Bradburn[2], Dr Praveen Thokala[5], Professor Tim Harris[6], Ms Maggie Marsh[7], Dr Alex Scott[2], Professor Cindy Cooper[2]

Corresponding author*: Dr Gordon Ward Fuller

Work address: Centre for Urgent and Emergency Care Research, School of Health and Related Research, University of Sheffield, Regent Court, 30 Regent Street, Sheffield, S1 4DA, UK

Telephone: +44 (0) 7968 280641

E-mail: g.fuller@sheffield.ac.uk

1. Centre for Urgent and Emergency Care Research, School of Health and Related Research, University of Sheffield, Regent Court, 30 Regent Street, Sheffield, S1 4DA, UK

2. Clinical Trials and Research Unit, School of Health and Related Research, University of Sheffield, Regent Court, 30 Regent Street, Sheffield, S1 4DA, UK

3. Warwick Clinical Trials Unit, University of Warwick, Coventry, CV4 7AL, UK

4. West Midlands Ambulance Service, Trust Headquarters, Millennium Point, Waterfront Business Park, Waterfront Way, Brierley Hill, West Midlands, DY5 1LX, UK

5. Health Economics and Decision Science, School of Health and Related Research, University of Sheffield, Regent Court, 30 Regent Street, Sheffield, S1 4DA, UK

For peer review only

6. School of Medicine and Dentistry, Blizard Institute, Barts and The London School of Medicine and Dentistry, 4 Newark Street, London, E1 2AT, UK

7. Sheffield Emergency Care Forum, Clinical Research Office Sheffield, Royal Hallamshire Hospital, D Floor, Glossop Road, Sheffield, S10 2JF

Word count (excluding title page, abstract, references, figures and tables): 3,528 words

Key words: Continuous positive airway pressure (CPAP), acute respiratory failure, emergency medical services, pilot studies, randomised controlled trial

Contributions of authors:

The following drafted the manuscript: Gordon Fuller (Chief Investigator), Steve Goodacre (Co-chief Investigator), Sam Keating (Trial Manager), Esther Herbert (Statistician), Mike Bradburn (Co-applicant, statistician), Praveen Thokala (Co-applicant, health economist), Josh Miller (Research paramedic), Maggie Marsh (Co-applicant, PPI representative).

The following conceived of, or designed, the work: GF, SG, SM, PT, MM, MB, Gavin D Perkins (Co-applicant), Matthew Ward (Co-applicant), Tim Harris (Co-applicant), MM, Alex Scott (Qualitative researcher), Cindy Cooper Co-applicant (CTRU Director).

The following were involved in the acquisition of data for the work: Andy Rosser (Lead Research Paramedic), Matthew Ward (Co-applicant), Imogen Gunson (Research paramedic), Josh Miller (Research paramedic)

The following were involved in the analysis and the interpretation of data for the work: GF, SG, SK, EH, MB, PT, JM, IG, MM, AS

The following contributed to the management and conduct of the trial: SK, Sarah Gonzalez (Trial support officer), AR, MW

All authors revised the work critically for important intellectual content and were involved in the final approval of the version to be published.

All authors agree to be accountable for all aspects of the work in ensuring that questions related to the accuracy or integrity of any part of the work are appropriately investigated and resolved.

For peer review only

Data Sharing Statement:

All data requests should be submitted to the corresponding author for consideration. Access to anonymised data may be granted following review.

Competing interests: Professor Steve Goodacre is Deputy Director of the NIHR HTA Programme, Chair of the NIHR HTA Commissioning Board and member of the NIHR HTA Funding Strategy Group. Professor Gavin Perkins is a NIHR Senior Investigator and member of the Programme Grants for Applied Research Board. Professor Cindy Cooper is a member of the NIHR Clinical Trials Unit Standing Advisory Committee and of the UK Clinical Research Collaboration Registered Clinical Trials Unit Network Executive Group. All authors have completed the unified competing interest form at www.icmje.org/coi_disclosure.pdf (available on request from the corresponding author) and declare (1) no financial support for the submitted work from anyone other than their employer; (2) no financial relationships with commercial entities that might have an interest in the submitted work; (3) no spouses, partners, or children with relationships with commercial entities that might have an interest in the submitted work; and (4) no non-financial interests that may be relevant to the submitted work.

For peer review only

ABSTRACT

Background

Acute respiratory failure (ARF) is a life-threatening emergency. Prehospital continuous positive airway pressure (CPAP) is a potentially beneficial treatment, however it is uncertain whether this could improve outcomes in NHS ambulance services. This study aimed to determine the feasibility of a large-scale definitive multicentre trial of prehospital CPAP in ARF.

Methods

A single centre, open label, individual patient randomised, controlled, external pilot trial was conducted. Adults with respiratory distress and peripheral oxygen saturations below British Thoracic Society (BTS) target levels were recruited from a single UK Ambulance Service. Patients were randomised to prehospital CPAP (O-Two system) versus standard oxygen therapy in a 1:1 ratio using simple randomisation. Primary objectives were to assess feasibility and acceptability of trial procedures. The primary clinical outcome was 30 day mortality.

Results

Between August 2017 and July 2018 77 patients were enrolled (target 120), including 7 cases with a diagnosis where CPAP could be ineffective or harmful. CPAP was fully delivered in 74% (target 75%). There were no major protocol violations/non-compliances. Full data were available for all key outcomes (targets ≥90%). Overall 30 day mortality was 27.3%. Of these deceased patients, 14/21 (68%) either did not have a respiratory condition or had ceiling of treatment decisions implemented excluding hospital non-invasive ventilation and critical care.

Conclusions

Recruitment rate was below target and feasibility was not demonstrated. Limited compliance with CPAP, and difficulty in identifying patients who could benefit from CPAP, indicate that prehospital CPAP is unlikely to materially reduce mortality. A definitive effectiveness trial of CPAP is therefore not recommended.

For peer review only

Strengths and limitations of this study

- This study provides important information regarding the feasibility of a large-scale definitive randomised controlled trial investigating prehospital CPAP in acute respiratory failure.
- Expert recommendations for pilot randomised controlled trials were followed.
- A pragmatic study design was used to maximise generalisability to real-life prehospital practice.
- A novel allocation concealment method using identical sealed boxes was used.
- Patients and clinicians were not blinded to the intervention.

For peer review only

BACKGROUND

Acute respiratory failure (ARF) is a common medical emergency caused by cardiac or respiratory diseases, including heart failure, pneumonia, chronic obstructive pulmonary disease (COPD) and asthma.[1] The incidence of ARF has been estimated at 80 cases per 100,000 per year in the United States.[1] Although varying according to the underlying cause, the overall risk of death is high, with estimates of 30 day mortality ranging between 12% and 20%.[2] ARF has substantial health services costs, with patients often requiring prolonged hospital stays, ventilatory support and critical care admissions.[3, 4] ARF is responsible for over 3 million National Health Service (NHS) bed days and hospital costs of £9.6 million per year in England.[5,6]

Current prehospital management of ARF in the NHS uses a standard management approach of controlled oxygen therapy; supplemented by specific ancillary treatments directed at the underlying disease.[7] This therapy is usually delivered by ambulance service personal without physician support. Continuous positive airways pressure (CPAP) is widely used in hospital to treat acute respiratory failure due to COPD, pneumonia, decompensated obstructive sleep apnoea, cardiogenic pulmonary oedema, and chest wall trauma;[8] and it has been suggested that it may be more effective if delivered earlier i.e. en route to hospital.[5] The difficulties of prehospital diagnosis mean that prehospital CPAP is likely to be applied generally to all cases of ARF, rather than directed towards those due to a specific cause.

Existing research investigating prehospital CPAP is inconclusive. A recent evidence synthesis suggested that prehospital CPAP may reduce the risk of mortality in ARF compared to standard treatment, but noted that the primary studies were relatively small, heterogeneous, at risk of bias, and were provided by specialist services, so may not be applicable to routine prehospital practice.[5] These findings suggest that although prehospital CPAP is a promising and potentially efficacious therapy, a large pragmatic trial is needed to confirm effectiveness when implemented as part of routine practice across a clinically relevant population with ARF.

Prior to a large pragmatic trial and economic evaluation, it is first necessary to estimate the incidence of eligible patients to determine whether a trial would be feasible and cost-effective. It is also important to determine whether prehospital CPAP can be delivered successfully in the context of the NHS ambulance services. For these reasons a stand-alone feasibility study is necessary to estimate the incidence of eligible patients, test the feasibility and acceptability of potential definitive trial methods, and address important uncertainties, such as patient selection, delivery of the intervention and event rates.

For peer review only

METHODS

Study design, aims and objectives

The ACUTE (Ambulance CPAP: Use, Treatment effect and Economics) study was an open label, individual randomised, pragmatic, parallel group, external pilot trial. It aimed to determine the feasibility of a definitive trial evaluating the effect of prehospital CPAP (intervention arm) compared to standard oxygen therapy (control arm) on 30-day mortality from ARF. The feasibility and effectiveness outcomes are listed in Table 1. The trial was registered prior to commencement with the International Standard Randomised Controlled Trials Number Registry (ISRCTN12048261) and a protocol, with pre-planned outcomes and analysis plan, has been previously published.[9] Health economic results will be reported separately.

Setting and study population

Recruitment took place across four hubs covering 1.5 million people in an English ambulance service between 1st August 2017 and August 2018 (last follow up competed 13th August 2018). Adults with respiratory distress and peripheral oxygen saturation below British Thoracic Society (BTS) target levels (88% for patients with COPD, 94% for other conditions) despite supplemental oxygen (titrated low flow oxygen for COPD, or titrated high flow oxygen in other conditions) were eligible.[10] Patients with pre-existing lack of capacity; or with limited potential to benefit from, or contraindications to CPAP, were excluded. Inclusion and exclusion criteria are detailed in Table 2 and were based on clinician judgement at the scene of incident.

For peer review only

Table 1. ACUTE trial objectives and outcomes

Feasibility outcomes	Feasibility targets
1. Rate of eligible patients per 100,000 population per year	•Target 8 per 100,000 per year i.e. 120 across the 1.5 million population of the 4 hubs
2. Proportion recruited and allocated to treatment appropriately	•Proportion recruited in error and classified as major or minor non-compliances (target 0% and ≤10%).
3. Adherence to allocated treatment	•Adherence to the allocation schedule (target ≥90%)<br>•Adherence to treatment in the CPAP arm (target ≥75%)
4. Retention and data completeness up to 30 days	•Retention at 30 days (target ≥90%)<br>•Data completeness (target ≥90%)

Clinical outcome measures:

1. Proportion surviving to 30 days (primary outcome for definitive trial)
2. Proportion undergoing endotracheal intubation by 30 days
3. Proportion admitted to critical care at any point up to 30 days
4. Mean and median length of hospital stay
5. Change in Visual Analogue Scale (VAS) dyspnoea score from presentation to immediately before ED arrival
6. Mean change in quality of life, measured with EQ-5D-5L[6]
7. Key elements of post-discharge health-care resource use up to 30 days

For peer review only

Table 2. ACUTE trial eligibility criteria

Inclusion criteria

ALL of:

- Adults ≥ 18 years
- Respiratory distress ,
- Hypoxia:
    - COPD: Sats <88% despite low flow oxygen
    - Other conditions: Sats <94% despite high flow oxygen.

Exclusion criteria

ANY of:

1. Hospital CPAP treatment available within 15 minutes of eligibility assessment.
2. Age < 18 years
3. Known to have terminal illness
4. Known pre-existing lack of capacity (confirmed by relatives, carers or documentary evidence, such as Lasting Power of Attorney)
5. Documented not for resuscitation status
6. Acutely incapacitated patients with known valid advanced directive declining non-invasive ventilation or participation in research
7. The patient has an oxygen alert card*
8. Anticipated inability to apply CPAP (e.g. facial deformity)
9. Respiratory failure due to chest trauma
10. Contraindication to CPAP (suspected pneumothorax, respiratory arrest, epistaxis, vomiting, hypotension)
11. Previous enrolment in the ACUTE trial
12. Pregnancy
13. Patients unable to communicate with ambulance service clinicians

*Patient-held card warning against use of high flow oxygen. Provided to patients with a history of previous hypercapnic acidosis with a PaO2 >10.0 kPa—indicating that oxygen may have worsened the hypercapnia.

For peer review only

Randomisation

Enrolled patients were individually allocated to CPAP or standard oxygen therapy in a 1:1 ratio using simple randomisation. The randomisation sequence was computer generated by an independent statistician not directly involved in the conduct of the trial. The allocation schedule was held centrally on a password-protected, access-restricted network drive. The trial statistician did not have access to the randomisation sequence until after data lock.

Participant enrolment and consent

Ambulance service clinicians (paramedics and ambulance technicians) volunteering to participate in the ACUTE study and trained in trial procedures identified potential participants with ARF when attending emergency 999 ambulance calls. In patients judged to have capacity, verbal consent was obtained for participation. Eligible patients lacking mental capacity were enrolled in the trial without consent if determined to be in their best interests. In all cases, a research paramedic reviewed the participant in hospital as soon as possible after enrolment, obtaining consent for further data collection and participation in the trial according to the provisions of the Mental Capacity Act 2005. [11,12] In the contingency that consent was declined, prehospital data was retained and anonymised 30 day mortality data collected. If a patient died before approach for written consent, ethical approval allowed for collection of anonymised prehospital, hospital and 30 day mortality data.

Allocation concealment and blinding

CPAP devices and high concentration oxygen therapy masks were packaged in identically appearing, numbered, shrink wrapped, tamper proof sealed, trial equipment boxes. Equipment boxes were independently assembled in an audited process in accordance with the randomisation sequence at Sheffield Clinical Trials Research Unit. Boxes were supplied to participating ambulance hubs, and held unordered by number, in a secure designated storage area. Boxes were subsequently signed in and out for each shift by participating clinicians. Research paramedics audited the location and condition of all boxes, and adherence to the allocation schedule on a weekly basis. Due to the physical differences between the CPAP device and standard oxygen mask it was not possible to subsequently blind patients, ambulance service clinicians, hospital clinicians, or outcome assessors to the treatment arms.[9]

For peer review only

Trial treatments

Immediately after enrolment, paramedics opened the trial equipment box and provide treatment according to whether a CPAP device or high concentration oxygen mask was supplied. Patients in the intervention arm were treated with the O-Two unit (O-Two Medical Technologies, Ontario, Canada; supplied by SP Services(UK), Telford, UK), a lightweight, portable, open, single use, low flow CPAP system.[7] CPAP was started at 5 cm H2O and then incrementally increased by 1 cm H2O every 2–5 min to a maximum of 15 cm H2O. Patients in the control arm received standard oxygen therapy using nasal cannula, an air entrainment Venturi mask, a simple face mask, or a non-rebreathing reservoir face mask. The choice of flow rate and oxygen delivery device was determined by ambulance service clinicians according to the patient's condition and peripheral oxygen saturation levels.

Treatment in both arms was targeted to BTS guidelines for peripheral oxygen saturations.[8] Target peripheral oxygen saturations were 88-92% for patients with known/suspected COPD and 94-98% for patients with other suspected causes of acute respiratory failure. Ancillary condition-specific treatments were administered in both trial arms according to Joint Royal Colleges Ambulance Liaison Committee (JRCALC) Clinical Practice Guidelines.[7] Subsequent hospital management, including emergency department CPAP or non-invasive ventilation, was at the discretion of the hospital clinician.

Data collection and safety reporting

Electronic patient records and computer aided dispatch data were screened by research paramedics to identify potentially eligible, but unenrolled, patients. Routinely collected prehospital data (e.g. timings, vital signs) were collated from participants' electronic records by research paramedics. Additional trial specific information was collected using a trial form completed by recruiting ambulance service clinicians and contained within each equipment box. At 30 days, research paramedics reviewed the hospital records to collate details of subsequent progress, treatments, and vital status at 30 days. Quality of life and post-discharge resource use was assessed by questionnaire at 30 days following enrolment, either in person if still in hospital; or by telephone or post if discharged. Adverse health changes in participants were defined, monitored, recorded and reported according to UK Health Research Authority guidance.[13]

For peer review only

Sample size and statistical analyses

The baseline characteristics of enrolled patients are reported descriptively for the whole trial population, and separately per treatment arm. Feasibility outcomes are reported descriptively for the whole trial population, together with their 95% confidence interval. Summary estimates of relative and absolute effectiveness outcomes are presented, overall and stratified by treatment arm, with 95% confidence intervals. An intention to treat analysis was used, using either a full analysis set, or a complete case analysis for endpoints with missing outcome data.[14]

The ACUTE feasibility study aimed to recruit 120 patients over 12 months, the minimum number recommended to estimate binary parameters for the sample size calculation of the full trial.[15] Mortality under standard care was estimated at 12% and for the full trial a 5% absolute reduction was postulated (i.e. to 7%) in the intervention arm.[1, 3, 4] This sample size would allow estimation of mortality to within a standard error of 2.7% for use in the sample size calculation for any definitive trial; and feasibility outcomes to be estimated with a precision of <5%. Achieving this target would also indicate a recruitment rate sufficient to deliver the sample size for a definitive trial; and an incidence of ARF necessary to deliver a potentially cost-effective prehospital CPAP service.

All analyses were conducted in the R statistical package (R Foundation for Statistical Computing, Vienna, Austria) in accordance with a pre-specified statistical analysis plan and CONSORT principles. Following review of blinded outcome data during trial management group meetings an unexpectedly high overall mortality rate was noted. A post-hoc descriptive analysis of the deceased patients was therefore conducted.

Trial oversight, ethics and governance

The trial received ethical approval from the NHS Leeds East Research Ethics Committee (Reference: 16/YH/0406). A protocol amendment from permuted block to simple randomisation was approved prior to recruitment (16th February 2017, REC reference: 16/YH/0406). This work was supported by the National Institute for Health Research's Health Technology Assessment Programme, grant number HTA15/08/40. The funder, and CPAP manufacturer and supplier, were not involved in study design, conduct or publication.

For peer review only

Public and Patient Involvement

The public and patients were fully involved in the ACUTE study from conception to dissemination. The research proposal was developed in partnership with a service user co-applicant and patient advisory groups. A service user advisory group was enlisted for collaboration throughout the project; and a lay co-applicant provided advice on trial matters, attended TMG meetings, supported study management, and contributed to the study report and interpretation of results.

For peer review only

RESULTS

Recruitment and consent

Over the recruitment period, 364 patients with ARF meeting ACUTE trial eligibility criteria presented from the 1.5 million population of four participating ambulance service hubs. 161 of those patients (44.2%) were attended by ACUTE trained ambulance service clinicians and could potentially have been recruited. Of these 161, 77 (47.8%) were enrolled in the trial. Slightly more participants were randomised to the CPAP intervention arm (42 cases), than to the standard oxygen control arm (35 cases). Consent for further data collection was declined by 9 patients (11.7%) and 17 patients (22.1%) died prior to research paramedic approach for consent. Figure 1 presents the participant flow for the trial.

Baseline characteristics

The trial population was relatively elderly (median age 71 years), predominantly male (62.3%), and severely unwell (median VAS breathlessness score 9/10, median pulse 115 beats/minute, median respiratory rate 34 breaths/minute, and median initial peripheral oxygen saturations of 78.5%). The most common final hospital diagnoses were COPD (n=21/65, 32.3%) and lower respiratory tract infection (LRTI, n=28/65, 43.1%). A minority of cases (n=4/65, 6.2%) had a non-respiratory primary hospital diagnosis comprising abdominal aortic aneurysm, myocardial infarction, sepsis (not further specified), and liver failure (ascites). Pre-randomisation characteristics were similar across trial arms as summarised in Table 3.

For peer review only

[Figure 1 here]

For peer review only

Figure 1. CONSORT diagram of participants' flow through the ACUTE pilot trial

Table 1. Baseline characteristics of ACUTE trial participants by arm

Baseline Variable	Descriptive statistic	CPAP<br>N = 42	Standard Oxygen Therapy<br>N = 35	Total<br>N = 77
Age	Median (IQR)	70.0 (60.5, 76.8)	73 (65.0, 77.0)	71 (62.0, 77.0)
Sex	Male	27 (64.3%)	21 (60.0%)	48 (62.3%)
Hospital ARF diagnosis*	n=	36	30	65
	Asthma	1 (2.8%)	1 (2.9%)	2 (3.0%)
	COPD	10 (27.8%)	11 (36.7%)	21 (31.8%)
	Heart Failure	4 (11.1%)	2 (6.7%)	6 (9.1%)
	LRTI	17 (47.2%)	11 (36.7%)	28 (42.4%)
	PE	1 (2.8%)	0 (0.0%)	1 (1.5%)
	Other	3 (8.3%)	4 (13.3%)	7 (10.8%)
Breathlessness at enrolment (VAS 0-10)	n=	41	35	76
	Median (IQR)	9.0 (8.0, 10.0)	9.0 (8.0, 9.5)	9.0 (8.0, 10.0)
First systolic blood pressure (mmHg)	n=	40	30	70
	Median (IQR)	136.0 (115.2, 150.5)	126.5 (112.0, 152.0)	134.5 (112.2, 152.0)
First Glasgow Coma Score	n=	42	35	77
	Median (IQR)	15.0 (14.0, 15.0)	15 (14.5, 15.0)	15 (14.0, 15.0)
First oxygen saturations (%)	n=	41	35	76
	Median (IQR)	78 (74.0, 85.0)	82 (75.5, 86.0)	78.5 (74.8, 86.0)
First pulse rate (bpm)	n=	42	33	75
	Median (IQR)	117.0 (105.0, 125.8)	111 (92.0, 121.0)	115 (100.0, 124.0)
First Respiratory rate (breaths/min)	n=	42	35	77
	Median (IQR)	36.0 (30.5, 40.0)	32 (24.0, 40.0)	34 (28.0, 40.0)
Duration between arrival at scene and	Median (IQR)	43.00 (34.00, 49.75)	36.00 (32.50, 46.50)	40.00 (34.00, 49.00)

For peer review only

Baseline Variable	Descriptive statistic	CPAP<br><br>N = 42	Standard Oxygen Therapy<br>N = 35	Total<br><br>N = 77
departure to hospital (mins)				
Duration between leaving the scene and arriving at hospital (mins)	Median (IQR)	13.00 (9.00, 18.75)	15.00 (10.00, 20.50)	13.00 (10.00, 20.00)

*Consent was declined for data collection in 9 cases, clinical records were unavailable in 2 cases, and in 1 case there was no clear underlying diagnosis apparent in the notes.
IQR: Interquartile range; bpm: beats per minute; COPD: Chronic Obstructive Pulmonary Disease; LRTI: Lower Respiratory Tract Infection.

For peer review only

Feasibility outcomes

Feasibility outcomes, compared to the pre-specified target, are summarised in Table 4. The sample size of 77 enrolled patients resulted in a recruitment rate of 5.1 (95% CI 4.1-6.4) per 100,000 population per year; falling short of the feasibility target of 8 per 100,000 population per year (i.e. 120 patients).

All patients were recruited appropriately with no major or minor protocol non-compliances (targets of ≤10% and 0% respectively). There was full adherence to the allocation schedule (feasibility target ≥90%). Treatment with CPAP was attempted in all patients enrolled in the intervention arm. All patients received appropriate standard oxygen management in the control arm, although one patient was enrolled who used their own CPAP machine.

CPAP was fully delivered as planned (i.e. administered until hospital arrival, or discontinued due to patient improvement after successful treatment) in 73.8% (31/42, target 75%) of intervention arm patients. CPAP was commenced in 90.5% of patients (38/42), with two patients refusing to wear the mask, one patient spontaneously improving and a fourth patient having a cardiac arrest prior to commencement. Of patients commencing CPAP, 31 (81.6%) continued with CPAP until they arrived at hospital: six did not tolerate CPAP and the remaining patient (with a final diagnosis of pulmonary fibrosis) was transferred to a standard oxygen non-rebreather mask due to non-improvement.

Full data were available for key outcomes, including all feasibility endpoints and vital status at 30 days, compared to the feasibility targets of ≥90% retention at 30 days and ≥90% data completeness. A small amount (<1%) of prehospital data describing baseline patient characteristics was not available due to missing values. A larger proportion of hospital data was missing secondary to lack of consent for collection (n=9, 11.7%), absence of local research approvals to access hospital data (n=2, 2.6%), or unclear or absent information in the hospital clinical records (differing across variables, ranging from n=0, 0%, for hospital length of stay to n=25, 32.5%, for emergency department management). Of patients alive at 30 days (n=56), 30 day follow up questionnaires examining quality of life and post-discharge health resource use, were fully completed by 40 patients (71.4%).

For peer review only

Table 4. Summary of feasibility results

Feasibility Outcome	Target	Result
Recruitment rate	8 per 100,000 population per year (i.e. 120 patients recruited	• 5.1 (95% CI 4.1-6.4) per 100,000 population per year<br>• 77 enrolled patients.
Major and minor non-compliances	0% and ≤10%	• 0% - major non-compliances<br>• 1.3% - minor non-compliance
Adherence to the allocation schedule	Target ≥90%	•100% adherence to allocation schedule
Adherence to treatment in the CPAP arm	Target ≥75%	• 74% -CPAP fully delivered as planned
Retention at 30 days	Target ≥90%	•100% - follow up for all feasibility endpoints and 30 day mortality
Data completeness	Target ≥90%	Data completeness for outcomes:<br>• 100% - feasibility outcomes<br>• 100% - 30 day mortality<br>• 81% - 30 day intubation<br>• 84% - admission to critical care<br>• 99% - clinician assessed breathlessness<br>• 86% - length of hospital stay<br>• 85% - baseline EQ-5D-5L*<br>• 71% - 30 day EQ-5D-5L*<br>• 73% - 30 day resource use*

*Of alive patients

For peer review only

Effectiveness and safety outcomes

Overall mortality of the study population was higher than expected with 27.3% (n=21/77) patients dying by 30 days. 28.6% (n=12/42) patients died by 30 days in the CPAP arm and 25.7% (n=9/35) in the standard oxygen arm (expected 12%). The absolute risk difference of mortality in CPAP group compared to standard oxygen therapy was 2.9% (95% CI -19.7-25.4%). An unplanned descriptive analysis of the deceased patients was undertaken to explore the circumstances for the unexpectedly high mortality rate. Data were not available for two cases due to lack of research approvals to access data, and two patients died from non-cardiorespiratory conditions not amenable to NIV (ruptured abdominal aneurysm and liver failure). Of the remaining 17 cases, 6 patients (35.3%) received hospital NIV (n=5/17, 29.4%) or mechanical ventilation (n=1, 5.9%). The other 11 patients had hospital ceiling of treatment decisions excluding hospital NIV and critical care management (11/17, 64/7%). Other secondary effectiveness outcomes are summarised in Table 5, and were similar across trial arms. Post-discharge resource use is summarized in supplementary file 1.

In total, 39 expected related serious adverse events (SAE) occurred in 34 patients (45.2% for the CPAP arm, 42.9% for the standard oxygen therapy arm). The majority were deaths (20/39, 51.2%) or re-admission to hospital within 30 days (13/39, 33.3%). There were no unexpected related SAEs. Two patients (1 intervention arm patient not receiving CPAP and 1 control arm patient) were categorised with related expected serious adverse events following diagnosis with pneumothoraces requiring intercostal drainage after hospital admission. There were no significant SAEs attributable to CPAP therapy. Adverse events are detailed in supplementary file 2.

For peer review only

Table 5. Summary of effectiveness outcomes

Effectiveness outcome	CPAP<br>N=42	Standard Oxygen Therapy<br>N=35	Total<br>N=77	Absolute risk difference (95% CI)
30 day mortality	n = 42<br>12<br>(28.6%)	n = 35<br>9<br>(25.7%)	n = 77<br>21<br>(27.3%)	2.9%<br>(-19.7 - 25.4)
Intubated	n = 33<br>2<br>(6.1%)	n = 29<br>1<br>(3.4%)	n = 62<br>3<br>(4.8%)	2.6%<br>(-10.5 - 15.7)
Admission to critical care	n = 35<br>4<br>(11.4%)	n = 30<br>2<br>(6.5%)	n = 65<br>6<br>(9.2%)	4.8%<br>(-12.1 – 21.7)
				Median of the difference (95% CI)
Median length of stay (days, IQR)	n = 22<br>10.0<br>(6.5, 12)	n = 22<br>7.0<br>(5, 9.8)	n = 44<br>8<br>(5.5, 11.2)	3.00<br>(-0.00, 6.00)
Change in patient reported breathlessness over prehospital interval (VAS, IQR)	n = 18<br>-3<br>(-4, -2)	n = 18<br>-2<br>(-4, -1)	n = 36<br>-2.5<br>(-4, -1)	-2.00<br>(-3.00, 0.00)
Change in clinician assessed breathlessness over prehospital interval (VAS, IQR)	n = 41<br>-3<br>(-5, -1)	n = 35<br>-2<br>(-3.5, 1)	n = 76<br>-2<br>(-4, -1)	-1<br>(-2, 0)
30 day EQ-5D-5L (IQR)	n = 22<br>0.82<br>(0.58, 0.95)	n = 18<br>0.73<br>(0.43, 0.89)	n = 40<br>0.76<br>(0.48, 0.92)	0.08<br>(-0.6, 0.26)
Median change in EQ-5D-5L (IQR)	n = 22<br>0.09<br>(-0.01, 0.16)	n = 18<br>0.10<br>(-0.06, 0.19)	n = 40<br>0.09<br>(-0.02, 0.18)	0.0 (-0.12, 0.16)

IQR: interquartile range; VAS: Visual analogue scale; EQ-5D-5L: EuroQOL-5D-5L Value Sets

For peer review only

DISCUSSION

Summary of results

Over 12 months 77 patients were enrolled, below the recruitment target of 120 participants. CPAP was fully delivered as planned in 74% of intervention arm patients (target 75%). There were no major protocol violations/non-compliances. Full data were available for key outcomes, including all feasibility endpoints and vital status at 30 days. Mortality was higher than expected, with 27.3% of patients dying by 30 days. Of the deceased patients 68% either did not have a respiratory condition or had explicit or implicit ceiling of treatment decisions excluding hospital NIV or critical care. Two patients, neither receiving CPAP, were diagnosed with a pneumothorax in the emergency department and were reported as expected related serious adverse events.

Interpretation

These findings suggest that pilot study methods could be used for a future definitive trial. Patients were appropriately recruited using a deferred consent model, there were no protocol non-compliances, adherence to the allocation schedule was complete, and full data were available for key outcomes. The ACUTE pilot trial demonstrated a recruitment rate that was below the target rate considered a priori necessary to deliver a definitive pragmatic trial. However, the majority of potentially eligible cases presented to non-trial trained staff and it is therefore possible that recruitment could be improved if a full trial mandated participation of all ambulance service personnel.

External pilot trials are not designed or powered to generate estimates of clinical effect that should be used for decision-making.[16-18] We therefore draw no conclusions from comparisons of outcomes between CPAP and standard care. However, a number of findings from the ACUTE pilot trial can be used to inform a judgement regarding whether it would be plausible for a large trial to detect an effect from CPAP on mortality and therefore whether a definitive trial might be worthwhile.

Firstly, it is apparent that prehospital identification of patients with the potential to benefit from CPAP may be challenging. A small, but significant minority of cases were ultimately diagnosed with conditions where CPAP could not conceivably be beneficial.[8, 19] It is also concerning that pneumothoraces requiring intercostal drainage were detected in 2 cases. Although neither patient received prehospital CPAP, the potential for iatrogenic harm if these cases had been allocated to the intervention arm is conspicuous.

For peer review only

Secondly, delivery of CPAP was relatively limited, with only 74% of intervention arm patients continuing treatment to hospital as planned. Although CPAP may be efficacious, the potential to demonstrate effectiveness would be restricted by lack of treatment compliance.

Thirdly, a key rationale for the implementation of prehospital CPAP is that earlier instigation of treatment will improve outcomes over and above the availability of hospital NIV.[5] In the pilot trial, relatively short on-scene times and conveyance times were recorded (median of 40 minutes and 13 minutes respectively). The potential time advantage from prehospital administration of CPAP may be too small to produce meaningful benefit.

Finally, illness severity was much higher than anticipated. The increased overall 30 day mortality risk of 27.3% might initially appear to offer a definitive trial a greater opportunity to detect a clinically relevant survival benefit. However, many enrolled patients had apparent treatment limitation decisions for ward level hospital care only, indicating a study population with a high prevalence of end-stage cardiorespiratory disease, multiple severe comorbidities, or very poor pre-morbid performance status where CPAP treatment might be futile and overly burdensome.

In summary, although the higher than expected mortality rate might suggest increased potential to detect an absolute difference in mortality, the challenges of providing prehospital CPAP, and characteristics of the patients who would benefit from CPAP, suggest limited potential to improve survival. It therefore appears unlikely that a trial powered to detect a plausible effect size could be designed.

Limitations

The purpose of a feasibility study is to determine if a large-scale trial can be performed.[18] Therefore, challenges that might be interpreted as weaknesses when appraising a definitive RCT (such as low rates of recruitment and adherence) actually represent important learning points in the feasibility setting. Modification of eligibility criteria to specify the presence of a primary cardio-respiratory diagnosis as an inclusion criteria, and exclusion of patients with home CPAP machines, could select a more appropriate study population. The use of retrospective case note review for hospital data collection improved the efficiency of data collection, but is associated with well recognised limitations that could have resulted in inaccurate measurement of study endpoints.

Generalisability

For peer review only

Trial eligibility criteria were broad, excluding only patients where CPAP was contraindicated (e.g. vomiting), where entry would likely not be appropriate (e.g. Do not resuscitate status, pre-existing loss of capacity), or where verbal consent was not possible (e.g. language barrier). The clinical findings reported herein should therefore have strong generalisability to patients presenting with ARF in NHS ambulance services. External validity to other settings, with different demographics or prehospital systems, is less clear. For example, international emergency medical services may have shorter on-scene times or prehospital physicians with ultrasound skills to detect pneumothorax before CPAP application. Furthermore, a novel CPAP device was used. Although there are considerable advantages to the studied O-Two unit including small size, low cost and simplicity, there may also be limitations compared to other more complex prehospital CPAP systems e.g. FiO2 and CPAP level are jointly determined by the oxygen flow rate. Consequently, efficacy could differ with other methods of delivering prehospital CPAP.

Comparison to previous literature

A series of recent observational studies have also demonstrated that CPAP can be implemented by EMS.[20-24] These studies are consistent with the ACUTE experience of sub-maximal CPAP adherence and difficult prehospital diagnosis, including treatment of patients with pneumothoraces. A recent systematic review identified 10 trials and quasi-randomised studies comparing prehospital NIV (including CPAP) with standard oxygen therapy.[5] Network meta-analysis suggested that prehospital CPAP is an effective treatment for ARF, with evidence that it reduces mortality (odds ratio 0.41; 95% CrI 0.20 to 0.77) and intubation rate (0.32; 95% CrI 0.17 to 0.62) compared to standard care.2 However, some included studies were at risk of selection bias from lack of allocation concealment and information bias, secondary to unblinded outcome assessment. Furthermore, the meta-analysis findings have doubtful external validity to prehospital practice in many settings. Only one trial included undifferentiated respiratory failure patients, and the methods used to deliver prehospital CPAP (physician or paramedics with online physician support) are not routine in many prehospital care systems.[5]

Conclusions

Pilot trial recruitment rate was below the target rate and feasibility was not demonstrated. Limited compliance with CPAP, and a study population including patients who could not benefit from CPAP, suggest that a clinically significant effect size is not plausible. A definitive effectiveness trial of CPAP is therefore not recommended. These findings also argue against routine implementation of CPAP into

For peer review only - http://bmjopen.bmj.com/site/about/guidelines.xhtml

many ambulance services; but would not preclude a CPAP service provided by clinicians with extended training (e.g. prehospital physicians) where advanced clinical skills might allow selective targeting of treatment to an appropriate subgroup of patients.

For peer review only

REFERENCES

1. Creagh-Brown B. Respiratory failure. Medicine 2016;**44**(6):342-45

2. Stefan MS, Shieh MS, Pekow PS, et al. Epidemiology and outcomes of acute respiratory failure in the United States, 2001 to 2009: a national survey. J. Hosp. Med. 2013;**8**(2):76-82

3. Ray P, Birolleau S, Lefort Y, et al. Acute respiratory failure in the elderly: etiology, emergency diagnosis and prognosis. Crit. Care 2006;**10**(3):R82

4. Delerme S, Ray P. Acute respiratory failure in the elderly: diagnosis and prognosis. Age Ageing 2008;**37**(3):251-57

5. Pandor A, Thokala P, Goodacre S, et al. Pre-hospital non-invasive ventilation for acute respiratory failure: a systematic review and cost-effectiveness evaluation. Health Technol. Assess. 2015;**19**(42):v-vi, 1-102

6. Health Do. Hospital Episode Statistics. Secondary Hospital Episode Statistics 2014. http://www.hesonline.nhs.uk

7. Fisher JD JRCALC, National Ambulance Service Medical Directors, and Association of Ambulance Chief Executives. *UK ambulance services clinical practice guidelines: 2013. Class Professional Publishing.*: Class Professional Publishing, 2013.

8. Non-invasive ventilation in acute respiratory failure. Thorax 2002;**57**(3):192-211

For peer review only

9. Fuller GW, Goodacre S, Keating S, et al. The ACUTE (Ambulance CPAP: Use, Treatment effect and economics) feasibility study: a pilot randomised controlled trial of prehospital CPAP for acute respiratory failure. Pilot and feasibility studies 2018;**4**:86-86

10. O'Driscoll BR, Howard LS, Earis J, et al. BTS guideline for oxygen use in adults in healthcare and emergency settings. Thorax 2017;**72**(Suppl 1):ii1-ii90

11. Herdman M, Gudex C, Lloyd A, et al. Development and preliminary testing of the new five-level version of EQ-5D (EQ-5D-5L). Qual. Life Res. 2011;**20**(10):1727-36

12. O_two CPAP unit. Secondary O_two CPAP unit 2018. http://otwo.com/emergency-cpap/o_two-single-use-cpap/.

13. Health Do. Health Research Authority Secondary Health Research Authority 2019. https://www.hra.nhs.uk/.

14. Kruse RL, Alper BS, Reust C, et al. Intention-to-treat analysis: who is in? Who is out? J. Fam. Pract. 2002;**51**(11):969-71

15. Teare MD, Dimairo M, Shephard N, et al. Sample size requirements to estimate key design parameters from external pilot randomised controlled trials: a simulation study. Trials 2014;**15**:264

For peer review only

16. Arain M, Campbell MJ, Cooper CL, et al. What is a pilot or feasibility study? A review of current practice and editorial policy. BMC Med. Res. Methodol. 2010;**10**:67-67

17. Leon AC, Davis LL, Kraemer HC. The role and interpretation of pilot studies in clinical research. J. Psychiatr. Res. 2011;**45**(5):626-29

18. Thabane L, Ma J, Chu R, et al. A tutorial on pilot studies: the what, why and how. BMC Med. Res. Methodol. 2010;**10**:1-1

19. Bello G, De Pascale G, Antonelli M. Noninvasive Ventilation. Clin. Chest Med. 2016;**37**(4):711-21

20. Hensel M, Strunden MS, Tank S, et al. Prehospital non-invasive ventilation in acute respiratory failure is justified even if the distance to hospital is short. Am. J. Emerg. Med. 2019;**37**(4):651-56

21. Jacob J, Arranz M, Sancho Ramoneda M, et al. [Noninvasive mechanical ventilation in emergency services in Catalonia: the VNICat registry cohort study]. Emergencias : revista de la Sociedad Espanola de Medicina de Emergencias 2017;**29**(1):33-38

22. Luiz T, Kumpch M, Gruttner J, et al. Prehospital CPAP Therapy by Emergency Physicians in Patients with Acute Respiratory Failure due to Acute Cardiogenic Pulmonary Edema or Acutely Exacerbated COPD. In Vivo 2016;**30**(2):133-9

23. Sahu N, Matthews P, Groner K, et al. Observational Study on Safety of Prehospital BLS CPAP in Dyspnea. Prehosp. Disaster Med. 2017;**32**(6):610-14

For peer review only

24. Willmore A, Dionne R, Maloney J, et al. Effectiveness and safety of a prehospital program of continuous positive airway pressure (CPAP) in an urban setting. Cjem 2015;**17**(6):609-16

For peer review only

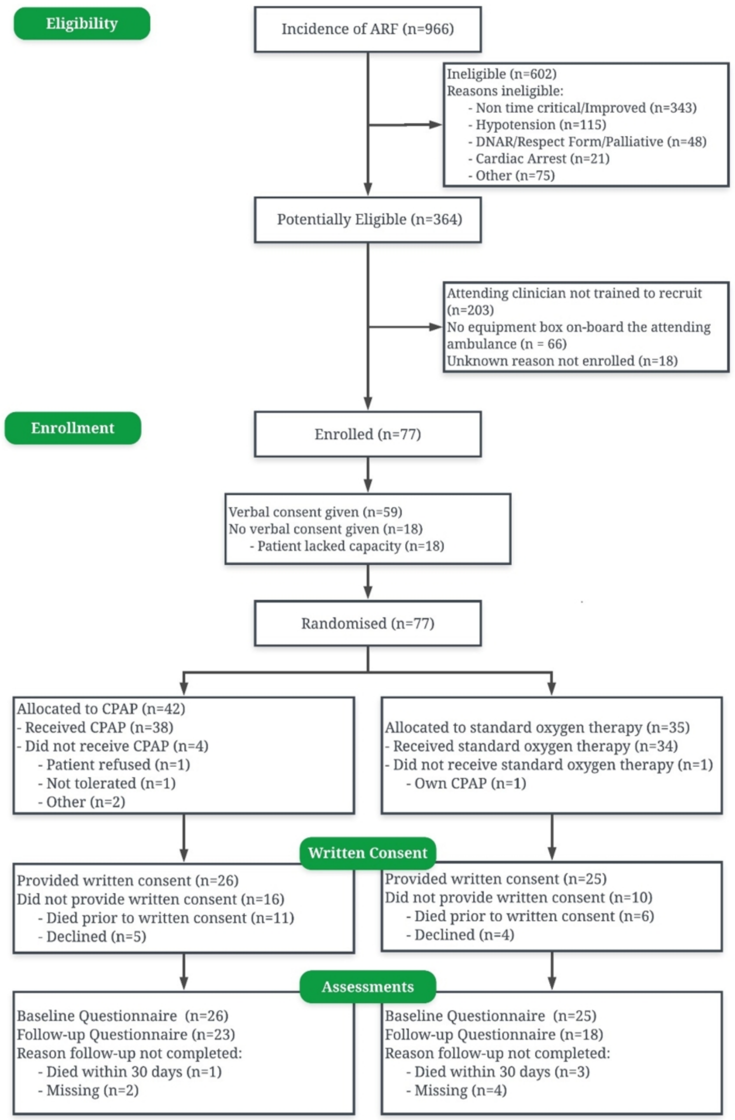

Figure 1. CONSORT diagram of participants' flow through the ACUTE pilot trial

224x313mm (300 x 300 DPI)

ACUTE pilot trial post-discharge healthcare resource use

Table showing post-discharge healthcare resource use

		CPAP	Standard Oxygen Therapy	Total
		**N = 42**	**N = 35**	**N = 77**
Discharged after index admission	n=	36	30	66
		22 (61.1%)	22 (73.3%)	44 (66.7%)
Readmission for any reason	n=	22	22	44
		5 (22.7%)	6 (27.3%)	11 (25.0%)
Healthcare provider consulted	n=	23	18	41
		14 (60.9%)	14 (77.8%)	28 (68.3%)
Healthcare provider seen	GP	7 (50.0%)	8 (57.1%)	15 (53.6%)
	Practice or district nurse	7 (50.0%)	5 (35.7%)	12 (42.9%)
	Emergency Department	4 (28.6%)	3 (21.4%)	7 (25.0%)
	Physiotherapist (out of hospital)	2 (14.3%)	3 (21.4%)	5 (17.9%)
	Other*	3 (21.4%)	7 (50.0%)	10 (35.7%)

*Other healthcare providers seen were: Oxygen supplier, care workers, community respiratory team, dentist, heart failure nurse, occupational therapy, outpatient appointment, outpatient x-ray, pharmacist, social worker. Some participants saw multiple other healthcare providers.

For peer review only

ACUTE pilot trial safety reporting procedures and adverse events

Flow chart detailing safety reporting procedures for the ACUTE study

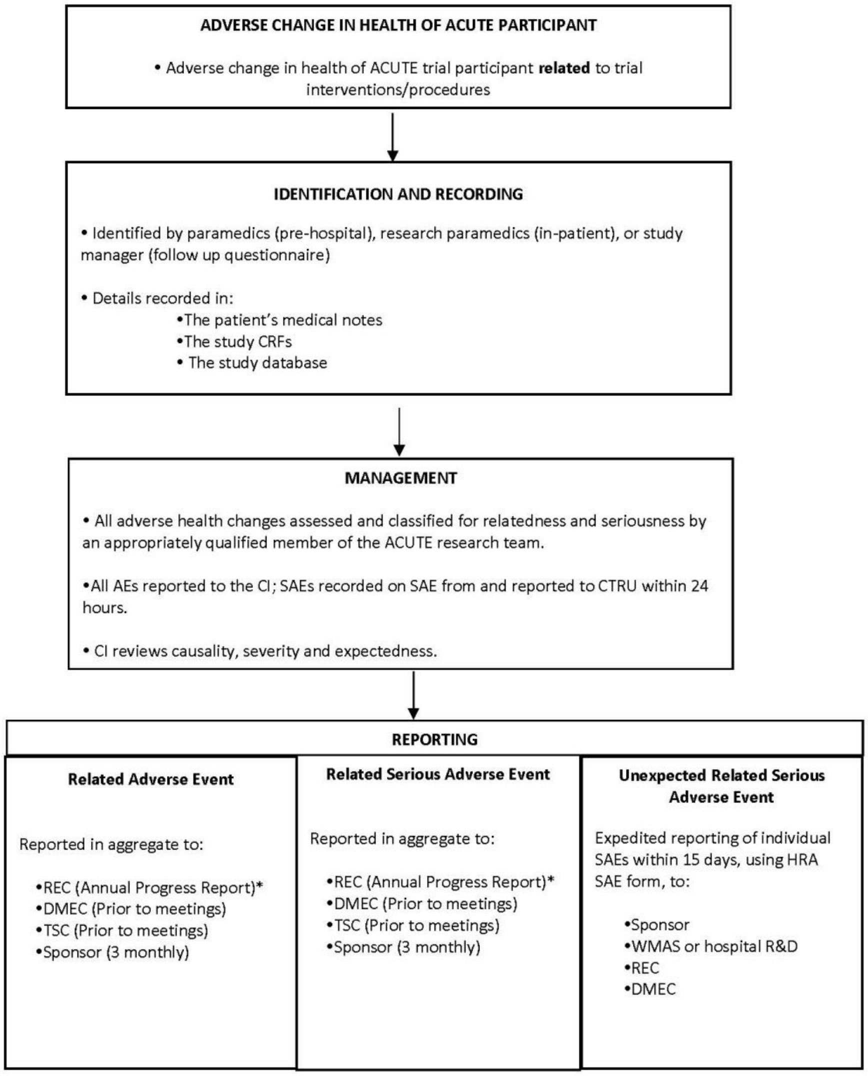

*Related AEs/SAEs resulting in treatment failure

Table of classification of adverse events.

Adverse Health Change Terminology*	Definition
**Seriousness**	
**Adverse Event (AE)**	An adverse change in health that occurs while a patient is taking part in a study
**Serious Adverse Event (SAE)**	Any adverse event occurring while a patient is taking part in a study, that results in:<br><br>• Death<br>• Life Threatening Illness<br>• Hospitalisation<br>• Prolongation of Hospitalisation<br>• Disability or Incapacity<br>• Congenital abnormality or birth defect<br>• Other adverse health change resulting in inability to perform routine activities or significant medical event
**Causality**	
**Unrelated**	An adverse change in health that occurs while a patient is taking part in a study which is not caused by or related to trial treatments.
**Related**	An adverse change in health that occurs while a patient is taking part in a study, which is caused by or related to trial treatments. An AE or SAE is considered related if the relationship between the event and trial treatments is:<br><br>• Possible - There is some evidence to suggest a causal relationship. However, the influence of other factors may have contributed to the event (e.g. the participant's clinical condition, other concomitant treatments).<br>• Probable - There is evidence to suggest a causal relationship and the influence of other factors is unlikely, or<br>• Definite - There is clear evidence to suggest a causal relationship and other possible contributing factors can be ruled out.
**Expectedness**	
**Unexpected**	Any adverse health change that is **NOT** consistent with the known and expected adverse events of trial treatments i.e. it is not listed in the protocol or related documents/literature as an expected occurrence.
**Expected**	Any adverse health change that **IS** consistent with the known and expected adverse events of trial treatments
**Severity***	
**Mild**	An adverse health change that does not interfere with routine activities
**Moderate**	An adverse health change that interferes with routine activities
**Severe**	An adverse health change that makes it impossible to perform routine activities

*The term 'severity', is used to describe the intensity, and should not be confused with 'serious' which is based on participant/event outcome or action criteria. For example, a headache may be severe but not serious, while a minor stroke is serious but may not be severe.

For peer review only

Table summary of the number of AEs and SAEs as well as the proportion of patients experiencing at least one AE or SAE for the ITT population

	CPAP	Standard Oxygen Therapy	Total
Patients	42	35	77
Number of AEs	27	20	47
Number of individuals with an AE	23 (54.8%)	17 (48.6%)	40 (51.9%)
Number of SAEs	22	17	39
Number of individuals with a SAE	19 (45.2%)	15 (42.9%)	34 (44.2%)

ACUTE pilot trial adverse events

Site	AE summary	AE category	Start date	End date	Serious?	Expected SAE?
Stoke	Headache	Other	19/08/2017	19/08/2017	No	No
Stoke	Arrested prior to receiving intervention	Cardiorespiratory arrest	13/09/2017	13/09/2017	Yes	No
Lichfield	Respiratory arrest	Other	17/09/2017	17/09/2017	Yes	No
Lichfield	Died prior to approach	Cardiorespiratory arrest	18/09/2017	18/09/2017	Yes	No
Stoke	Fractured NOF	Other	18/10/2017		Yes	No
Erdington	Social Care readmission	Other	23/10/2017	08/12/2017	Yes	No
Lichfield	CVA	Other	28/10/2017	15/12/2017	Yes	No
Stafford	Death	Cardiorespiratory arrest	29/10/2017	29/10/2017	Yes	Yes
Stafford	Death	Cardiorespiratory arrest	29/10/2017	29/10/2017	Yes	Yes
Stoke	Pt died	Cardiorespiratory arrest	16/11/2017	16/11/2017	Yes	No
Stoke	Re-admission within 30 days	Other	30/11/2017	30/11/2017	No	Yes
Stoke	Claustrophobia	Claustrophobia	12/12/2017	12/12/2017	No	Yes
Erdington	Distress at mask	Other	15/12/2017	15/12/2017	No	Yes

For peer review only

Erdington	Died within 30 days	Cardiorespiratory arrest	21/12/2017	21/12/2017	Yes	No
Stafford	Pt died	Cardiorespiratory arrest	29/12/2017	29/12/2017	Yes	Yes
Stoke	Mild claustrophobia	Claustrophobia	05/01/2018	05/01/2018	No	Yes
Erdington	Readmission to respiratory ward with pneumonia	Other	05/01/2018	15/01/2018	Yes	Yes
Stafford	Re-admission	Other	10/01/2018		Yes	No
Stoke	Death	Cardiorespiratory arrest	14/01/2018	14/01/2018	Yes	Yes
Stoke	Pt died	Cardiorespiratory arrest	17/01/2018	17/01/2018	Yes	Yes
Stafford	Pt died on day of recruitment	Cardiorespiratory arrest	23/01/2018	23/01/2018	Yes	Yes
Stafford	Died ahead of 30 day follow up	Cardiorespiratory arrest	27/01/2018	27/01/2018	Yes	Yes
Stafford	Died on elderly care ward	Cardiorespiratory arrest	28/01/2018	28/01/2018	Yes	Yes
Stoke	Agitation and distress with mask	Progressive respiratory distress	28/01/2018	28/01/2018	No	Yes
Erdington	Death	Cardiorespiratory arrest	06/02/2018	06/02/2018	Yes	Yes
Stafford	Patient died	Cardiorespiratory arrest	20/02/2018	20/02/2018	Yes	Yes
Lichfield	Died prior to 30 day	Cardiorespiratory arrest	03/03/2018	03/03/2018	Yes	Yes
Stafford	Pt re-admitted to hospital during 30-day period after recruitment	Other	08/03/2018	15/03/2018	Yes	Yes
Lichfield	Died prior to approach	Cardiorespiratory arrest	09/03/2018	09/03/2018	Yes	Yes
Erdington	Died following discharge prior to approach	Cardiorespiratory arrest	10/03/2018	10/03/2018	Yes	Yes
Lichfield	Died prior to approach	Cardiorespiratory arrest	17/03/2018	17/03/2018	Yes	Yes

For peer review only

Stoke	Pneumothorax	Tension pneumothorax	28/03/2018	28/03/2018	Yes	Yes
Stoke	Patient got worse	Vomiting	28/03/2018	28/03/2018	No	Yes
Stafford	Pt re-admitted, then died	Other	12/04/2018	15/04/2018	Yes	Yes
Lichfield	Mask wouldn't inflate	Other	17/04/2018	17/04/2018	No	No
Lichfield	Cardiac arrest	Cardiorespiratory arrest	08/05/2018	08/05/2018	Yes	Yes
Lichfield	Death	Cardiorespiratory arrest	31/05/2018	31/05/2018	Yes	Yes
Stafford	Died prior to approach	Cardiorespiratory arrest	03/06/2018	03/06/2018	Yes	Yes
Lichfield	Readmissions within 30 days	Other	30/06/2018	01/07/2018	Yes	Yes
Lichfield	Readmission within 30 days	Other	02/07/2018	06/07/2018	Yes	Yes
Lichfield	Claustrophobia and Progressive Respiratory Distress	Other	05/07/2018	05/07/2018	No	Yes
Lichfield	Pneumothorax	Other	05/07/2018	27/07/2018	Yes	Yes
Stafford	Re-admission	Other	06/07/2018		Yes	Yes
Lichfield	Readmission	Other	07/07/2018	27/07/2018	Yes	Yes
Stoke	Pt re-admitted during 30-day reference period	Other	25/07/2018	03/08/2018	Yes	Yes
Lichfield	Readmission	Other	28/07/2018		Yes	Yes
Stoke	Death	Cardiorespiratory arrest	22/08/2018	22/08/2018	Yes	Yes

For peer review only

BMJ Open

Is a Definitive Trial of Prehospital Continuous Positive Airway Pressure versus standard oxygen therapy for Acute Respiratory Failure Indicated? The ACUTE pilot randomised controlled trial

Journal:	*BMJ Open*
Manuscript ID	bmjopen-2019-035915.R2
Article Type:	Original research
Date Submitted by the Author:	27-May-2020

Complete List of Authors:	Fuller, Gordon; The University of Sheffield, School of Health and Related Research<br>Keating, Sam; The University of Sheffield, School of Health and Related Research<br>Goodacre, Steve; University of Sheffield, School of Health and Related Research<br>Herbert, Esther; The University of Sheffield, School of Health and Related Research<br>Perkins, Gavin; University of Warwick, Clinical Trials Unit<br>Rosser, Andy; West Midlands Ambulance Service NHS Foundation Trust<br>Gunson, Imogen; West Midlands Ambulance Service NHS Foundation Trust<br>Miller, Josh; West Midlands Ambulance Service NHS Foundation Trust<br>ward, matthew; West Midlands Ambulance Service NHS Foundation Trust<br>Bradburn, Mike; University of Sheffield, School of Health and Related Research<br>Thokala, Praveen; The University of Sheffield, School of Health and Related Research<br>Harris, Tim; Bart's Health/NHS, Emergency Medicine<br>Marsh, Maggie; Sheffield Emergency Care Forum<br>Scott, Alex; The University of Sheffield, School of Health and Related Research<br>Cooper, Cindy; University of Sheffield, Clinical Trials Research Unit

**Primary Subject Heading**:	Emergency medicine
Secondary Subject Heading:	Cardiovascular medicine, Respiratory medicine
Keywords:	Adult thoracic medicine < THORACIC MEDICINE, Adult intensive & critical care < INTENSIVE & CRITICAL CARE, ACCIDENT & EMERGENCY MEDICINE

I, the Submitting Author has the right to grant and does grant on behalf of all authors of the Work (as defined in the below author licence), an exclusive licence and/or a non-exclusive licence for contributions from authors who are: i) UK Crown employees; ii) where BMJ has agreed a CC-BY licence shall apply, and/or iii) in accordance with the terms applicable for US Federal Government officers or employees acting as part of their official duties; on a worldwide, perpetual, irrevocable, royalty-free basis to BMJ Publishing Group Ltd ("BMJ") its licensees and where the relevant Journal is co-owned by BMJ to the co-owners of the Journal, to publish the Work in this journal and any other BMJ products and to exploit all rights, as set out in our licence.

The Submitting Author accepts and understands that any supply made under these terms is made by BMJ to the Submitting Author unless you are acting as an employee on behalf of your employer or a postgraduate student of an affiliated institution which is paying any applicable article publishing charge ("APC") for Open Access articles. Where the Submitting Author wishes to make the Work available on an Open Access basis (and intends to pay the relevant APC), the terms of reuse of such Open Access shall be governed by a Creative Commons licence – details of these licences and which Creative Commons licence will apply to this Work are set out in our licence referred to above.

Other than as permitted in any relevant BMJ Author's Self Archiving Policies, I confirm this Work has not been accepted for publication elsewhere, is not being considered for publication elsewhere and does not duplicate material already published. I confirm all authors consent to publication of this Work and authorise the granting of this licence.

For peer review only

Is a Definitive Trial of Prehospital Continuous Positive Airway Pressure versus standard oxygen therapy for Acute Respiratory Failure Indicated? The ACUTE pilot randomised controlled trial

Dr Gordon W Fuller[1]*, Dr Samuel Keating[2], Professor Steve Goodacre[1], Ms Esther Herbert[2], Professor Gavin D Perkins[3], Mr Andy Rosser[4], Ms Imogen Gunson[4], Mr Joshua Miller[4], Mr Matthew Ward[4], Mr Mike Bradburn[2], Dr Praveen Thokala[5], Professor Tim Harris[6], Ms Maggie Marsh[7], Dr Alex Scott[2], Professor Cindy Cooper[2]

Corresponding author*: Dr Gordon Ward Fuller

Work address: Centre for Urgent and Emergency Care Research, School of Health and Related Research, University of Sheffield, Regent Court, 30 Regent Street, Sheffield, S1 4DA, UK

Telephone: +44 (0) 7968 280641

E-mail: g.fuller@sheffield.ac.uk

1. Centre for Urgent and Emergency Care Research, School of Health and Related Research, University of Sheffield, Regent Court, 30 Regent Street, Sheffield, S1 4DA, UK

2. Clinical Trials and Research Unit, School of Health and Related Research, University of Sheffield, Regent Court, 30 Regent Street, Sheffield, S1 4DA, UK

3. Warwick Clinical Trials Unit, University of Warwick, Coventry, CV4 7AL, UK

4. West Midlands Ambulance Service, Trust Headquarters, Millennium Point, Waterfront Business Park, Waterfront Way, Brierley Hill, West Midlands, DY5 1LX, UK

5. Health Economics and Decision Science, School of Health and Related Research, University of Sheffield, Regent Court, 30 Regent Street, Sheffield, S1 4DA, UK

For peer review only

6. School of Medicine and Dentistry, Blizard Institute, Barts and The London School of Medicine and Dentistry, 4 Newark Street, London, E1 2AT, UK

7. Sheffield Emergency Care Forum, Clinical Research Office Sheffield, Royal Hallamshire Hospital, D Floor, Glossop Road, Sheffield, S10 2JF

Word count (excluding title page, abstract, references, figures and tables): 3,528 words

Key words: Continuous positive airway pressure (CPAP), acute respiratory failure, emergency medical services, pilot studies, randomised controlled trial

Contributions of authors:

The following drafted the manuscript: Gordon Fuller (Chief Investigator), Steve Goodacre (Co-chief Investigator), Sam Keating (Trial Manager), Esther Herbert (Statistician), Mike Bradburn (Co-applicant, statistician), Praveen Thokala (Co-applicant, health economist), Josh Miller (Research paramedic), Maggie Marsh (Co-applicant, PPI representative).

The following conceived of, or designed, the work: GF, SG, SM, PT, MM, MB, Gavin D Perkins (Co-applicant), Matthew Ward (Co-applicant), Tim Harris (Co-applicant), MM, Alex Scott (Qualitative researcher), Cindy Cooper Co-applicant (CTRU Director).

The following were involved in the acquisition of data for the work: Andy Rosser (Lead Research Paramedic), Matthew Ward (Co-applicant), Imogen Gunson (Research paramedic), Josh Miller (Research paramedic)

The following were involved in the analysis and the interpretation of data for the work: GF, SG, SK, EH, MB, PT, JM, IG, MM, AS

The following contributed to the management and conduct of the trial: SK, Sarah Gonzalez (Trial support officer), AR, MW

All authors revised the work critically for important intellectual content and were involved in the final approval of the version to be published.

All authors agree to be accountable for all aspects of the work in ensuring that questions related to the accuracy or integrity of any part of the work are appropriately investigated and resolved.

For peer review only

Data Sharing Statement:

All data requests should be submitted to the corresponding author for consideration. Access to anonymised data may be granted following review.

Competing interests: Professor Steve Goodacre is Deputy Director of the NIHR HTA Programme, Chair of the NIHR HTA Commissioning Board and member of the NIHR HTA Funding Strategy Group. Professor Gavin Perkins is a NIHR Senior Investigator and member of the Programme Grants for Applied Research Board. Professor Cindy Cooper is a member of the NIHR Clinical Trials Unit Standing Advisory Committee and of the UK Clinical Research Collaboration Registered Clinical Trials Unit Network Executive Group. All authors have completed the unified competing interest form at www.icmje.org/coi_disclosure.pdf (available on request from the corresponding author) and declare (1) no financial support for the submitted work from anyone other than their employer; (2) no financial relationships with commercial entities that might have an interest in the submitted work; (3) no spouses, partners, or children with relationships with commercial entities that might have an interest in the submitted work; and (4) no non-financial interests that may be relevant to the submitted work.

Funding statement: This work was supported by the National Institute for Health Research's Health Technology Assessment Programme, grant number HTA15/08/40. The funder, and CPAP manufacturer and supplier, were not involved in study design, conduct or publication.

For peer review only

ABSTRACT

Background

Acute respiratory failure (ARF) is a life-threatening emergency. Prehospital continuous positive airway pressure (CPAP) is a potentially beneficial treatment, however it is uncertain whether this could improve outcomes in NHS ambulance services. This study aimed to determine the feasibility of a large-scale definitive multicentre trial of prehospital CPAP in ARF.

Methods

A single centre, open label, individual patient randomised, controlled, external pilot trial was conducted. Adults with respiratory distress and peripheral oxygen saturations below British Thoracic Society (BTS) target levels despite controlled oxygen treatment were recruited from a single UK Ambulance Service. Patients were randomised to prehospital CPAP (O-Two system) versus standard oxygen therapy in a 1:1 ratio using simple randomisation. Primary objectives were to assess feasibility and acceptability of trial procedures. The primary clinical outcome was 30 day mortality.

Results

Between August 2017 and July 2018 77 patients were enrolled (target 120), including 7 cases with a diagnosis where CPAP could be ineffective or harmful. CPAP was fully delivered in 74% (target 75%). There were no major protocol violations. Full data were available for all key outcomes (targets ≥90%). Overall 30 day mortality was 27.3%. Of these deceased patients, 14/21 (68%) either did not have a respiratory condition or had ceiling of treatment decisions implemented excluding hospital non-invasive ventilation and critical care.

Conclusions

Recruitment rate was below target and feasibility was not demonstrated. Limited compliance with CPAP, and difficulty in identifying patients who could benefit from CPAP, indicate that prehospital CPAP is unlikely to materially reduce mortality. A definitive effectiveness trial of CPAP is therefore not recommended.

For peer review only

Strengths and limitations of this study

- This study provides important information regarding the feasibility of a large-scale definitive randomised controlled trial investigating prehospital CPAP in acute respiratory failure.
- Expert recommendations for pilot randomised controlled trials were followed.
- A pragmatic study design was used to maximise generalisability to real-life prehospital practice.
- A novel allocation concealment method using identical sealed boxes was used.
- Patients and clinicians were not blinded to the intervention.

For peer review only

BACKGROUND

Acute respiratory failure (ARF) is a common medical emergency caused by cardiac or respiratory diseases, including heart failure, pneumonia, chronic obstructive pulmonary disease (COPD) and asthma.[1] The incidence of ARF has been estimated at 80 cases per 100,000 per year in the United States.[1] Although varying according to the underlying cause, the overall risk of death is high, with estimates of 30 day mortality ranging between 12% and 20%.[2] ARF has substantial health services costs, with patients often requiring prolonged hospital stays, ventilatory support and critical care admissions.[3, 4] ARF is responsible for over 3 million National Health Service (NHS) bed days and hospital costs of £9.6 million per year in England.[5,6]

Current prehospital management of ARF in the NHS uses a standard management approach of controlled oxygen therapy; supplemented by specific ancillary treatments directed at the underlying disease.[7] This therapy is usually delivered by ambulance service personnel without physician support. Continuous positive airways pressure (CPAP) is widely used in hospital to treat acute respiratory failure due to COPD, pneumonia, decompensated obstructive sleep apnoea, cardiogenic pulmonary oedema, and chest wall trauma;[8] and it has been suggested that it may be more effective if delivered earlier i.e. en route to hospital.[5] The difficulties of prehospital diagnosis mean that prehospital CPAP is likely to be applied generally to all cases of ARF, rather than directed towards those due to a specific cause.

Existing research investigating prehospital CPAP is inconclusive. A recent evidence synthesis suggested that prehospital CPAP may reduce the risk of mortality in ARF compared to standard treatment, but noted that the primary studies were relatively small, heterogeneous, at risk of bias, and were provided by specialist services, so may not be applicable to routine prehospital practice.[5] These findings suggest that although prehospital CPAP is a promising and potentially efficacious therapy, a large pragmatic trial is needed to confirm effectiveness when implemented as part of routine practice across a clinically relevant population with ARF.

Prior to a large pragmatic trial and economic evaluation, it is first necessary to estimate the incidence of eligible patients to determine whether a trial would be feasible and cost-effective. It is also important to determine whether prehospital CPAP can be delivered successfully in the context of the NHS ambulance services. For these reasons a stand-alone feasibility study is necessary to estimate the incidence of eligible patients, test the feasibility and acceptability of potential definitive trial methods, and address important uncertainties, such as patient selection, delivery of the intervention and event rates.

For peer review only

METHODS

Study design, aims and objectives

The ACUTE (Ambulance CPAP: Use, Treatment effect and Economics) study was an open label, individual randomised, pragmatic, parallel group, external pilot trial. It aimed to determine the feasibility of a definitive trial evaluating the effect of prehospital CPAP (intervention arm) compared to standard oxygen therapy (control arm) on 30-day mortality from ARF. The feasibility and effectiveness outcomes are listed in Table 1. The trial was registered prior to commencement with the International Standard Randomised Controlled Trials Number Registry (ISRCTN12048261) and a protocol, with pre-planned outcomes and analysis plan, has been previously published.[9] Health economic results will be reported separately.

Setting and study population

Recruitment took place across four hubs covering 1.5 million people in an English ambulance service between 1st August 2017 and August 2018 (last follow up completed 13th August 2018). Adults with respiratory distress and peripheral oxygen saturation below British Thoracic Society (BTS) target levels (88% for patients with COPD, 94% for other conditions) despite supplemental oxygen (titrated low flow oxygen for COPD, or titrated high flow oxygen in other conditions) were eligible.[10] Patients with pre-existing lack of capacity; or with limited potential to benefit from, or contraindications to CPAP, were excluded. Inclusion and exclusion criteria are detailed in Table 2 and were based on clinician judgement at the scene of incident.

For peer review only

Table 1. ACUTE trial objectives and outcomes

Feasibility outcomes	Feasibility targets
1. Rate of eligible patients per 100,000 population per year	•Target 8 per 100,000 per year i.e. 120 across the 1.5 million population of the 4 hubs
2. Proportion recruited and allocated to treatment appropriately	•Proportion recruited in error and classified as major or minor non-compliances (target 0% and ≤10%).
3. Adherence to allocated treatment	•Adherence to the allocation schedule (target ≥90%)<br>•Adherence to treatment in the CPAP arm (target ≥75%)
4. Retention and data completeness up to 30 days	•Retention at 30 days (target ≥90%)<br>•Data completeness (target ≥90%)
**Clinical outcome measures:**	
1. Proportion surviving to 30 days (primary outcome for definitive trial)	
2. Proportion undergoing endotracheal intubation by 30 days	
3. Proportion admitted to critical care at any point up to 30 days	
4. Mean and median length of hospital stay	
5. Change in Visual Analogue Scale (VAS) dyspnoea score from presentation to immediately before ED arrival	
6. Mean change in quality of life, measured with EQ-5D-5L[6]	
7. Key elements of post-discharge health-care resource use up to 30 days	

For peer review only

Table 2. ACUTE trial eligibility criteria

Inclusion criteria

ALL of:

- Adults ≥ 18 years
- Respiratory distress ,
- Hypoxia:
  - COPD: Sats <88% despite low flow oxygen
  - Other conditions: Sats <94% despite high flow oxygen.

Exclusion criteria

ANY of:

1. Hospital CPAP treatment available within 15 minutes of eligibility assessment.
2. Age < 18 years
3. Known to have terminal illness
4. Known pre-existing lack of capacity (confirmed by relatives, carers or documentary evidence, such as Lasting Power of Attorney)
5. Documented not for resuscitation status
6. Acutely incapacitated patients with known valid advanced directive declining non-invasive ventilation or participation in research
7. The patient has an oxygen alert card*
8. Anticipated inability to apply CPAP (e.g. facial deformity)
9. Respiratory failure due to chest trauma
10. Contraindication to CPAP (suspected pneumothorax, respiratory arrest, epistaxis, vomiting, hypotension)
11. Previous enrolment in the ACUTE trial
12. Pregnancy
13. Patients unable to communicate with ambulance service clinicians

*Patient-held card warning against use of high flow oxygen. Provided to patients with a history of previous hypercapnic acidosis with a PaO_2 >10.0 kPa—indicating that oxygen may have worsened the hypercapnia.

For peer review only

Randomisation

Enrolled patients were individually allocated to CPAP or standard oxygen therapy in a 1:1 ratio using simple randomisation. The randomisation sequence was computer generated by an independent statistician not directly involved in the conduct of the trial. The allocation schedule was held centrally on a password-protected, access-restricted network drive. The trial statistician did not have access to the randomisation sequence until after data lock.

Participant enrolment and consent

Ambulance service clinicians (paramedics and ambulance technicians) volunteering to participate in the ACUTE study and trained in trial procedures identified potential participants with ARF when attending emergency 999 ambulance calls. In patients judged to have capacity, verbal consent was obtained for participation. Eligible patients lacking mental capacity were enrolled in the trial without consent if determined to be in their best interests. In all cases, a research paramedic reviewed the participant in hospital as soon as possible after enrolment, obtaining consent for further data collection and participation in the trial according to the provisions of the Mental Capacity Act 2005. [11,12] In the contingency that consent was declined, prehospital data was retained and anonymised 30 day mortality data collected. If a patient died before approach for written consent, ethical approval allowed for collection of anonymised prehospital, hospital and 30 day mortality data.

Allocation concealment and blinding

CPAP devices and high concentration oxygen therapy masks were packaged in identically appearing, numbered, shrink wrapped, tamper proof sealed, trial equipment boxes. Equipment boxes were independently assembled in an audited process in accordance with the randomisation sequence at Sheffield Clinical Trials Research Unit. Boxes were supplied to participating ambulance hubs, and held unordered by number, in a secure designated storage area. Boxes were subsequently signed in and out for each shift by participating clinicians. Research paramedics audited the location and condition of all boxes, and adherence to the allocation schedule on a weekly basis. Due to the physical differences between the CPAP device and standard oxygen mask it was not possible to subsequently blind patients, ambulance service clinicians, hospital clinicians, or outcome assessors to the treatment arms.[9]

For peer review only

Trial treatments

Immediately after enrolment, paramedics opened the trial equipment box and provided treatment according to whether a CPAP device or high concentration oxygen mask was supplied. Patients in the intervention arm were treated with the O-Two unit (O-Two Medical Technologies, Ontario, Canada; supplied by SP Services(UK), Telford, UK), a lightweight, portable, open, single use, low flow CPAP system.[7] CPAP was started at 5 cm H2O and then incrementally increased by 1 cm H2O every 2–5 min to a maximum of 15 cm H2O. Patients in the control arm received standard oxygen therapy using nasal cannula, an air entrainment Venturi mask, a simple face mask, or a non-rebreathing reservoir face mask. The choice of flow rate and oxygen delivery device was titrated by ambulance service clinicians according to the patient's condition, and peripheral oxygen saturation levels.

Treatment in both arms was targeted to BTS guidelines for peripheral oxygen saturations.[8] Target peripheral oxygen saturations were 88-92% for patients with known/suspected COPD and 94-98% for patients with other suspected causes of acute respiratory failure. Ancillary condition-specific treatments were administered in both trial arms according to Joint Royal Colleges Ambulance Liaison Committee (JRCALC) Clinical Practice Guidelines.[7] Subsequent hospital management, including emergency department CPAP or non-invasive ventilation, was at the discretion of the hospital clinician.

Data collection and safety reporting

Electronic patient records and computer aided dispatch data were screened by research paramedics to identify potentially eligible, but unenrolled, patients. Routinely collected prehospital data (e.g. timings, vital signs) were collated from participants' electronic records by research paramedics. Additional trial specific information was collected using a trial form completed by recruiting ambulance service clinicians and contained within each equipment box. At 30 days, research paramedics reviewed the hospital records to collate details of subsequent progress, treatments, and vital status at 30 days. Quality of life and post-discharge resource use was assessed by questionnaire at 30 days following enrolment, either in person if still in hospital; or by telephone or post if discharged. Adverse health changes in participants were defined, monitored, recorded and reported according to UK Health Research Authority guidance.[13]

For peer review only

Sample size and statistical analyses

The baseline characteristics of enrolled patients are reported descriptively for the whole trial population, and separately per treatment arm. Feasibility outcomes are reported descriptively for the whole trial population, together with their 95% confidence interval. Summary estimates of relative and absolute effectiveness outcomes are presented, overall and stratified by treatment arm, with 95% confidence intervals. An intention to treat analysis was used, using either a full analysis set, or a complete case analysis for endpoints with missing outcome data.[14]

The ACUTE feasibility study aimed to recruit 120 patients over 12 months, the minimum number recommended to estimate binary parameters for the sample size calculation of the full trial.[15] Mortality under standard care was estimated at 12% and for the full trial a 5% absolute reduction was postulated (i.e. to 7%) in the intervention arm.[1, 3, 4] This sample size would allow estimation of mortality to within a standard error of 2.7% for use in the sample size calculation for any definitive trial; and feasibility outcomes to be estimated with a precision of <5%. Achieving this target would also indicate a recruitment rate sufficient to deliver the sample size for a definitive trial; and an incidence of ARF necessary to deliver a potentially cost-effective prehospital CPAP service.

All analyses were conducted in the R statistical package (R Foundation for Statistical Computing, Vienna, Austria) in accordance with a pre-specified statistical analysis plan and CONSORT principles. Following review of blinded outcome data during trial management group meetings an unexpectedly high overall mortality rate was noted. A post-hoc descriptive analysis of the deceased patients was therefore conducted.

Trial oversight, ethics and governance

The trial received ethical approval from the NHS Leeds East Research Ethics Committee (Reference: 16/YH/0406). A protocol amendment from permuted block to simple randomisation was approved prior to recruitment (16th February 2017, REC reference: 16/YH/0406).

Funding statement

This work was supported by the National Institute for Health Research's Health Technology Assessment Programme, grant number HTA15/08/40. The funder, and CPAP manufacturer and supplier, were not involved in study design, conduct or publication.

For peer review only

Public and Patient Involvement

The public and patients were fully involved in the ACUTE study from conception to dissemination. The research proposal was developed in partnership with a service user co-applicant and patient advisory groups. A service user advisory group was enlisted for collaboration throughout the project; and a lay co-applicant provided advice on trial matters, attended TMG meetings, supported study management, and contributed to the study report and interpretation of results.

For peer review only

RESULTS

Recruitment and consent

Over the recruitment period, 364 patients with ARF meeting ACUTE trial eligibility criteria presented from the 1.5 million population of four participating ambulance service hubs. 161 of those patients (44.2%) were attended by ACUTE trained ambulance service clinicians and could potentially have been recruited. Of these 161, 77 (47.8%) were enrolled in the trial. Slightly more participants were randomised to the CPAP intervention arm (42 cases), than to the standard oxygen control arm (35 cases). Consent for further data collection was declined by 9 patients (11.7%) and 17 patients (22.1%) died prior to research paramedic approach for consent. Figure 1 presents the participant flow for the trial.

Baseline characteristics

The trial population was relatively elderly (median age 71 years), predominantly male (62.3%), and severely unwell (median VAS breathlessness score 9/10, median pulse 115 beats/minute, median respiratory rate 34 breaths/minute, and median initial peripheral oxygen saturations of 78.5%). The most common final hospital diagnoses were COPD (n=21/65, 32.3%) and lower respiratory tract infection (LRTI, n=28/65, 43.1%). A minority of cases (n=4/65, 6.2%) had a non-respiratory primary hospital diagnosis comprising abdominal aortic aneurysm, myocardial infarction, sepsis (not further specified), and liver failure (ascites). Pre-randomisation characteristics were similar across trial arms as summarised in Table 3.

For peer review only

[Figure 1 here]

For peer review only

Figure 1. CONSORT diagram of participants' flow through the ACUTE pilot trial

Table 3. Baseline characteristics of ACUTE trial participants by arm

Baseline Variable	Descriptive statistic	CPAP<br>N = 42	Standard Oxygen Therapy<br>N = 35	Total<br>N = 77
Age	Median (IQR)	70.0 (60.5, 76.8)	73.0 (65.0, 77.0)	71.0 (62.0, 77.0)
Sex	Male	27 (64.3%)	21 (60.0%)	48 (62.3%)
Hospital ARF diagnosis*	n=	36	30	66
	Asthma	1 (2.8%)	1 (2.9%)	2 (3.0%)
	COPD	10 (27.8%)	11 (36.7%)	21 (31.8%)
	Heart Failure	4 (11.1%)	2 (6.7%)	6 (9.1%)
	LRTI	17 (47.2%)	11 (36.7%)	28 (42.4%)
	Pulmonary embolism	1 (2.8%)	0 (0.0%)	1 (1.5%)
	Other	3 (8.3%)	4 (13.3%)	7 (10.8%)
Breathlessness at enrolment (VAS 0-10)	n=	41	35	76
	Median (IQR)	9.0 (8.0, 10.0)	9.0 (8.0, 9.5)	9.0 (8.0, 10.0)
First systolic blood pressure (mmHg)	n=	40	30	70
	Median (IQR)	136.0 (115.2, 150.5)	126.5 (112.0, 152.0)	134.5 (112.2, 152.0)
First Glasgow Coma Score	n=	42	35	77
	Median (IQR)	15.0 (14.0, 15.0)	15 (14.5, 15.0)	15 (14.0, 15.0)
First oxygen saturations (%)	n=	41	35	76
	Median (IQR)	78 (74.0, 85.0)	82 (75.5, 86.0)	78.5 (74.8, 86.0)
First pulse rate (bpm)	n=	42	33	75
	Median (IQR)	117.0 (105.0, 125.8)	111 (92.0, 121.0)	115 (100.0, 124.0)
First Respiratory rate (breaths/min)	n=	42	35	77
	Median (IQR)	36.0 (30.5, 40.0)	32 (24.0, 40.0)	34 (28.0, 40.0)
Duration between arrival at scene and	Median (IQR)	43.0 (34.0, 49.8)	36.0 (32.5, 46.5)	40.00 (34.0, 49.0)

For peer review only

Baseline Variable	Descriptive statistic	CPAP<br><br>N = 42	Standard Oxygen Therapy<br>N = 35	Total<br><br>N = 77
departure to hospital (mins)				
Duration between leaving the scene and arriving at hospital (mins)	Median (IQR)	13.0 (9.00, 18.75)	15.0 (10.0, 20.5)	13.0 (10.0, 20.0)

*Consent was declined for data collection in 9 cases, clinical records were unavailable in 2 cases, and in 1 case there was no clear underlying diagnosis apparent in the notes.
IQR: Interquartile range; bpm: beats per minute; COPD: Chronic Obstructive Pulmonary Disease; LRTI: Lower Respiratory Tract Infection.

For peer review only

Feasibility outcomes

Feasibility outcomes, compared to the pre-specified target, are summarised in Table 4. The sample size of 77 enrolled patients resulted in a recruitment rate of 5.1 (95% CI 4.1-6.4) per 100,000 population per year; falling short of the feasibility target of 8 per 100,000 population per year (i.e. 120 patients).

All patients were recruited appropriately with no major or minor protocol non-compliances (targets of ≤10% and 0% respectively). There was full adherence to the allocation schedule (feasibility target ≥90%). Treatment with CPAP was attempted in all patients enrolled in the intervention arm. All patients received appropriate standard oxygen management in the control arm, although one patient was enrolled who used their own CPAP machine.

CPAP was fully delivered as planned (i.e. administered until hospital arrival, or discontinued due to patient improvement after successful treatment) in 73.8% (31/42, target 75%) of intervention arm patients. CPAP was commenced in 90.5% of patients (38/42), with two patients refusing to wear the mask, one patient spontaneously improving and a fourth patient having a cardiac arrest prior to commencement. Of patients commencing CPAP, 31 (81.6%) continued with CPAP until they arrived at hospital: six did not tolerate CPAP and the remaining patient (with a final diagnosis of pulmonary fibrosis) was transferred to a standard oxygen non-rebreather mask due to non-improvement.

Full data were available for key outcomes, including all feasibility endpoints and vital status at 30 days, compared to the feasibility targets of ≥90% retention at 30 days and ≥90% data completeness. A small amount (<1%) of prehospital data describing baseline patient characteristics was not available due to missing values. A larger proportion of hospital data was missing secondary to lack of consent for collection (n=9, 11.7%), absence of local research approvals to access hospital data (n=2, 2.6%), or unclear or absent information in the hospital clinical records (differing across variables, ranging from n=0, 0%, for hospital length of stay to n=25, 32.5%, for emergency department management). Of patients alive at 30 days (n=56), 30 day follow up questionnaires examining quality of life and post-discharge health resource use, were fully completed by 40 patients (71.4%).

For peer review only

Table 4. Summary of feasibility results

Feasibility Outcome	Target	Result
Recruitment rate	8 per 100,000 population per year (i.e. 120 patients recruited	• 5.1 (95% CI 4.1-6.4) per 100,000 population per year<br>• 77 enrolled patients.
Major and minor non-compliances	0% and ≤10%	• 0% - major non-compliances<br>• 1.3% - minor non-compliance
Adherence to the allocation schedule	Target ≥90%	•100% adherence to allocation schedule
Adherence to treatment in the CPAP arm	Target ≥75%	• 74% -CPAP fully delivered as planned
Retention at 30 days	Target ≥90%	•100% - follow up for all feasibility endpoints and 30 day mortality
Data completeness	Target ≥90%	Data completeness for outcomes:<br>• 100% - feasibility outcomes<br>• 100% - 30 day mortality<br>• 81% - 30 day intubation<br>• 84% - admission to critical care<br>• 99% - clinician assessed breathlessness<br>• 86% - length of hospital stay<br>• 85% - baseline EQ-5D-5L*<br>• 71% - 30 day EQ-5D-5L*<br>• 73% - 30 day resource use*

*Of alive patients

For peer review only

Effectiveness and safety outcomes

Overall mortality of the study population was higher than expected with 27.3% (n=21/77) patients dying by 30 days. 28.6% (n=12/42) patients died by 30 days in the CPAP arm and 25.7% (n=9/35) in the standard oxygen arm (expected 12%). The absolute risk difference of mortality in CPAP group compared to standard oxygen therapy was 2.9% (95% CI -19.7-25.4%). An unplanned descriptive analysis of the deceased patients was undertaken to explore the circumstances for the unexpectedly high mortality rate. Data were not available for two cases due to lack of research approvals to access data, and two patients died from non-cardiorespiratory conditions not amenable to NIV (ruptured abdominal aneurysm and liver failure). Of the remaining 17 cases, 6 patients (35.3%) received hospital NIV (n=5/17, 29.4%) or mechanical ventilation (n=1, 5.9%). The other 11 patients had hospital ceiling of treatment decisions excluding hospital NIV and critical care management (11/17, 64/7%). Other secondary effectiveness outcomes are summarised in Table 5, and were similar across trial arms. Post-discharge resource use is summarized in supplementary file 1.

In total, 39 expected related serious adverse events (SAE) occurred in 34 patients (45.2% for the CPAP arm, 42.9% for the standard oxygen therapy arm). The majority were deaths (20/39, 51.2%) or re-admission to hospital within 30 days (13/39, 33.3%). There were no unexpected related SAEs. Two patients (1 intervention arm patient not receiving CPAP and 1 control arm patient) were categorised with related expected serious adverse events following diagnosis with pneumothoraces requiring intercostal drainage after hospital admission. There were no significant SAEs attributable to CPAP therapy. Adverse events are detailed in supplementary file 2.

For peer review only

Table 5. Summary of effectiveness outcomes

Effectiveness outcome	CPAP N=42	Standard Oxygen Therapy N=35	Total N=77	Absolute risk difference (95% CI)
30 day mortality	n = 42<br>12<br>(28.6%)	n = 35<br>9<br>(25.7%)	n = 77<br>21<br>(27.3%)	2.9%<br>(-19.7 - 25.4)
Intubated	n = 33<br>2<br>(6.1%)	n = 29<br>1<br>(3.4%)	n = 62<br>3<br>(4.8%)	2.6%<br>(-10.5 - 15.7)
Admission to critical care	n = 35<br>4<br>(11.4%)	n = 30<br>2<br>(6.5%)	n = 65<br>6<br>(9.2%)	4.8%<br>(-12.1 – 21.7)
				Median of the difference (95% CI)
Median length of stay (days, IQR)	n = 22<br>10.0<br>(6.5, 12)	n = 22<br>7.0<br>(5, 9.8)	n = 44<br>8<br>(5.5, 11.2)	3.00<br>(-0.00, 6.00)
Change in patient reported breathlessness over prehospital interval (VAS, IQR)	n = 18<br>-3<br>(-4, -2)	n = 18<br>-2<br>(-4, -1)	n = 36<br>-2.5<br>(-4, -1)	-2.00<br>(-3.00, 0.00)
Change in clinician assessed breathlessness over prehospital interval (VAS, IQR)	n = 41<br>-3<br>(-5, -1)	n = 35<br>-2<br>(-3.5, 1)	n = 76<br>-2<br>(-4, -1)	-1<br>(-2, 0)
30 day EQ-5D-5L (IQR)	n = 22<br>0.82<br>(0.58, 0.95)	n = 18<br>0.73<br>(0.43, 0.89)	n = 40<br>0.76<br>(0.48, 0.92)	0.08<br>(-0.6, 0.26)
Median change in EQ-5D-5L (IQR)	n = 22<br>0.09<br>(-0.01, 0.16)	n = 18<br>0.10<br>(-0.06, 0.19)	n = 40<br>0.09<br>(-0.02, 0.18)	0.0 (-0.12, 0.16)

IQR: interquartile range; VAS: Visual analogue scale; EQ-5D-5L: EuroQOL-5D-5L Value Sets

DISCUSSION

For peer review only

For peer review only - http://bmjopen.bmj.com/site/about/guidelines.xhtml

Summary of results

Over 12 months 77 patients were enrolled, below the recruitment target of 120 participants. CPAP was fully delivered as planned in 74% of intervention arm patients (target 75%). There were no major protocol violations. Full data were available for key outcomes, including all feasibility endpoints and vital status at 30 days. Mortality was higher than expected, with 27.3% of patients dying by 30 days. Of the deceased patients 68% either did not have a respiratory condition or had explicit or implicit ceiling of treatment decisions excluding hospital NIV or critical care. Two patients, neither receiving CPAP, were diagnosed with a pneumothorax in the emergency department and were reported as expected related serious adverse events.

Interpretation

These findings suggest that pilot study methods could be used for a future definitive trial. Patients were appropriately recruited using a deferred consent model, there were no protocol violations, adherence to the allocation schedule was complete, and full data were available for key outcomes. The ACUTE pilot trial demonstrated a recruitment rate that was below the target rate considered a priori necessary to deliver a definitive pragmatic trial. However, the majority of potentially eligible cases presented to non-trial trained staff and it is therefore possible that recruitment could be improved if a full trial mandated participation of all ambulance service personnel.

External pilot trials are not designed or powered to generate estimates of clinical effect that should be used for decision-making.[16-18] We therefore draw no conclusions from comparisons of outcomes between CPAP and standard care. However, a number of findings from the ACUTE pilot trial can be used to inform a judgement regarding whether it would be plausible for a large trial to detect an effect from CPAP on mortality and therefore whether a definitive trial might be worthwhile.

Firstly, it is apparent that prehospital identification of patients with the potential to benefit from CPAP may be challenging. A small, but significant minority of cases were ultimately diagnosed with conditions where CPAP could not conceivably be beneficial.[8, 19] It is also concerning that pneumothoraces requiring intercostal drainage were detected in 2 cases. Although neither patient received prehospital CPAP, the potential for iatrogenic harm is conspicuous.

Secondly, delivery of CPAP was relatively limited, with 74% of intervention arm patients continuing treatment to hospital as planned. Although CPAP may be efficacious, the potential to demonstrate effectiveness would be restricted by lack of treatment compliance.

For peer review only

Thirdly, a key rationale for the implementation of prehospital CPAP is that earlier instigation of treatment will improve outcomes over and above the availability of hospital NIV.[5] In the pilot trial, relatively short on-scene times and conveyance times were recorded (median of 40 minutes and 13 minutes respectively). The potential time advantage from prehospital administration of CPAP may be too small to produce meaningful benefit.

Finally, illness severity was much higher than anticipated. The increased overall 30 day mortality risk of 27.3% might initially appear to offer a definitive trial a greater opportunity to detect a clinically relevant survival benefit. However, many enrolled patients had apparent treatment limitation decisions for ward level hospital care only, indicating a study population with a high prevalence of end-stage cardiorespiratory disease, multiple severe comorbidities, or very poor pre-morbid performance status where CPAP treatment might be futile and overly burdensome. However, it should be noted that CPAP could also affect important patient centred outcomes such as symptom relief, which might be detected by differences in dyspnoea VAS scores.

In summary, although the higher than expected mortality rate might suggest increased potential to detect an absolute difference in mortality, the challenges of providing prehospital CPAP, and characteristics of the patients who would benefit from CPAP, suggest limited potential to improve survival. It therefore appears unlikely that a trial powered to detect a plausible effect size could be designed.

Limitations

The purpose of a feasibility study is to determine if a large-scale trial can be performed.[18] Therefore, challenges that might be interpreted as weaknesses when appraising a definitive RCT (such as low rates of recruitment and adherence) actually represent important learning points in the feasibility setting. Modification of eligibility criteria to specify the presence of a primary cardio-respiratory diagnosis as an inclusion criteria, and exclusion of patients with home CPAP machines, could select a more appropriate study population. The use of retrospective case note review for hospital data collection improved the efficiency of data collection, but is associated with well recognised limitations that could have resulted in inaccurate measurement of study endpoints. Furthermore, collection of more detailed data on NIV treatment in the emergency department would be beneficial in any future trial.

Generalisability

For peer review only

Trial eligibility criteria were broad, excluding only patients where CPAP was contraindicated (e.g. vomiting), where entry would likely not be appropriate (e.g. Do not resuscitate status, pre-existing loss of capacity), or where verbal consent was not possible (e.g. language barrier). The clinical findings reported herein should therefore have strong generalisability to patients presenting with ARF in NHS ambulance services. External validity to other settings, with different demographics or prehospital systems, is less clear. For example, international emergency medical services may have shorter on-scene times, use CPAP in less severe ARF, or use prehospital physicians with ultrasound skills to detect pneumothorax before CPAP application. Furthermore, a novel CPAP device was used. Although there are considerable advantages to the studied O-Two unit including small size, low cost and simplicity, there may also be limitations compared to other more complex prehospital CPAP systems e.g. FiO2 and CPAP level are jointly determined by the oxygen flow rate. Consequently, efficacy could differ with other methods of delivering prehospital CPAP.

Comparison to previous literature

A series of recent observational studies have also demonstrated that CPAP can be implemented by EMS.[20-24] These studies are consistent with the ACUTE experience of sub-maximal CPAP adherence and difficult prehospital diagnosis, including treatment of patients with pneumothoraces. A recent systematic review identified 10 trials and quasi-randomised studies comparing prehospital NIV (including CPAP) with standard oxygen therapy.[5] Network meta-analysis suggested that prehospital CPAP is an effective treatment for ARF, with evidence that it reduces mortality (odds ratio 0.41; 95% CrI 0.20 to 0.77) and intubation rate (0.32; 95% CrI 0.17 to 0.62) compared to standard care.2 However, some included studies were at risk of selection bias from lack of allocation concealment and information bias, secondary to unblinded outcome assessment. Furthermore, the meta-analysis findings have doubtful external validity to prehospital practice in many settings. Only one trial included undifferentiated respiratory failure patients, and the methods used to deliver prehospital CPAP (physician or paramedics with online physician support) are not routine in many prehospital care systems, reflected in higher levels of CPAP compliance.[5]

Conclusions

Pilot trial recruitment rate was below the target rate. Limited compliance with CPAP, and a study population including patients who could not benefit from CPAP, suggest that a clinically significant effect size is not plausible. A definitive effectiveness trial of CPAP is therefore not recommended. These

For peer review only - http://bmjopen.bmj.com/site/about/guidelines.xhtml

findings also argue against routine implementation of CPAP into many ambulance services; but would not preclude a CPAP service provided by clinicians with extended training (e.g. prehospital physicians) where advanced clinical skills might allow selective targeting of treatment to an appropriate subgroup of patients.

For peer review only

REFERENCES

1. Creagh-Brown B. Respiratory failure. Medicine 2016;**44**(6):342-45

2. Stefan MS, Shieh MS, Pekow PS, et al. Epidemiology and outcomes of acute respiratory failure in the United States, 2001 to 2009: a national survey. J. Hosp. Med. 2013;**8**(2):76-82

3. Ray P, Birolleau S, Lefort Y, et al. Acute respiratory failure in the elderly: etiology, emergency diagnosis and prognosis. Crit. Care 2006;**10**(3):R82

4. Delerme S, Ray P. Acute respiratory failure in the elderly: diagnosis and prognosis. Age Ageing 2008;**37**(3):251-57

5. Pandor A, Thokala P, Goodacre S, et al. Pre-hospital non-invasive ventilation for acute respiratory failure: a systematic review and cost-effectiveness evaluation. Health Technol. Assess. 2015;**19**(42):v-vi, 1-102

6. Health Do. Hospital Episode Statistics. Secondary Hospital Episode Statistics 2014. http://www.hesonline.nhs.uk

7. Fisher JD JRCALC, National Ambulance Service Medical Directors, and Association of Ambulance Chief Executives. *UK ambulance services clinical practice guidelines: 2013. Class Professional Publishing.*: Class Professional Publishing, 2013.

8. Non-invasive ventilation in acute respiratory failure. Thorax 2002;**57**(3):192-211

For peer review only

9. Fuller GW, Goodacre S, Keating S, et al. The ACUTE (Ambulance CPAP: Use, Treatment effect and economics) feasibility study: a pilot randomised controlled trial of prehospital CPAP for acute respiratory failure. Pilot and feasibility studies 2018;**4**:86-86

10. O'Driscoll BR, Howard LS, Earis J, et al. BTS guideline for oxygen use in adults in healthcare and emergency settings. Thorax 2017;**72**(Suppl 1):ii1-ii90

11. Herdman M, Gudex C, Lloyd A, et al. Development and preliminary testing of the new five-level version of EQ-5D (EQ-5D-5L). Qual. Life Res. 2011;**20**(10):1727-36

12. O_two CPAP unit. Secondary O_two CPAP unit 2018. http://otwo.com/emergency-cpap/o_two-single-use-cpap/.

13. Health Do. Health Research Authority Secondary Health Research Authority 2019. https://www.hra.nhs.uk/.

14. Kruse RL, Alper BS, Reust C, et al. Intention-to-treat analysis: who is in? Who is out? J. Fam. Pract. 2002;**51**(11):969-71

15. Teare MD, Dimairo M, Shephard N, et al. Sample size requirements to estimate key design parameters from external pilot randomised controlled trials: a simulation study. Trials 2014;**15**:264

For peer review only

16. Arain M, Campbell MJ, Cooper CL, et al. What is a pilot or feasibility study? A review of current practice and editorial policy. BMC Med. Res. Methodol. 2010;**10**:67-67

17. Leon AC, Davis LL, Kraemer HC. The role and interpretation of pilot studies in clinical research. J. Psychiatr. Res. 2011;**45**(5):626-29

18. Thabane L, Ma J, Chu R, et al. A tutorial on pilot studies: the what, why and how. BMC Med. Res. Methodol. 2010;**10**:1-1

19. Bello G, De Pascale G, Antonelli M. Noninvasive Ventilation. Clin. Chest Med. 2016;**37**(4):711-21

20. Hensel M, Strunden MS, Tank S, et al. Prehospital non-invasive ventilation in acute respiratory failure is justified even if the distance to hospital is short. Am. J. Emerg. Med. 2019;**37**(4):651-56

21. Jacob J, Arranz M, Sancho Ramoneda M, et al. [Noninvasive mechanical ventilation in emergency services in Catalonia: the VNICat registry cohort study]. Emergencias : revista de la Sociedad Espanola de Medicina de Emergencias 2017;**29**(1):33-38

22. Luiz T, Kumpch M, Gruttner J, et al. Prehospital CPAP Therapy by Emergency Physicians in Patients with Acute Respiratory Failure due to Acute Cardiogenic Pulmonary Edema or Acutely Exacerbated COPD. In Vivo 2016;**30**(2):133-9

23. Sahu N, Matthews P, Groner K, et al. Observational Study on Safety of Prehospital BLS CPAP in Dyspnea. Prehosp. Disaster Med. 2017;**32**(6):610-14

For peer review only

24. Willmore A, Dionne R, Maloney J, et al. Effectiveness and safety of a prehospital program of continuous positive airway pressure (CPAP) in an urban setting. Cjem 2015;**17**(6):609-16

For peer review only

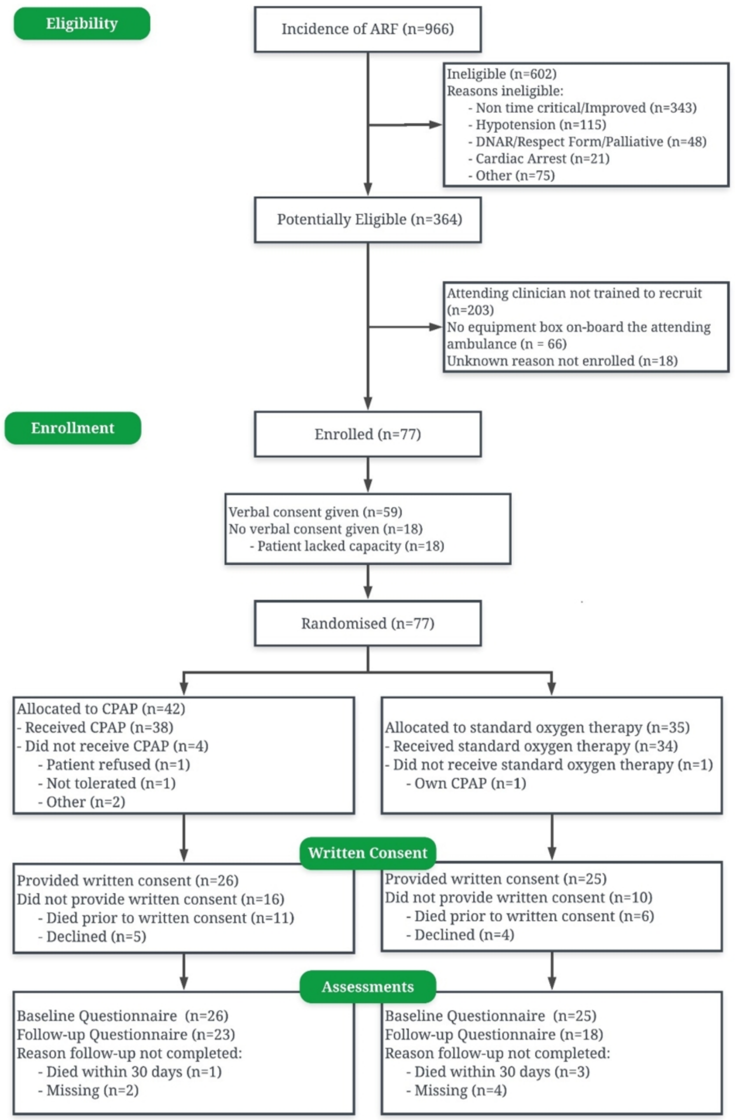

Figure 1. CONSORT diagram of participants' flow through the ACUTE pilot trial

224x313mm (300 x 300 DPI)

ACUTE pilot trial post-discharge healthcare resource use

Table showing post-discharge healthcare resource use up to 30 days after enrolment

		CPAP<br>N = 42	Standard Oxygen Therapy<br>N = 35	Total<br>N = 77
Discharged after index admission	n=	36	30	66
		22 (61.1%)	22 (73.3%)	44 (66.7%)
Readmission for any reason	n=	22	22	44
		5 (22.7%)	6 (27.3%)	11 (25.0%)
Healthcare provider consulted	n=	23	18	41
		14 (60.9%)	14 (77.8%)	28 (68.3%)
Healthcare provider seen	GP	7 (50.0%)	8 (57.1%)	15 (53.6%)
	Practice or district nurse	7 (50.0%)	5 (35.7%)	12 (42.9%)
	Emergency Department	4 (28.6%)	3 (21.4%)	7 (25.0%)
	Physiotherapist (out of hospital)	2 (14.3%)	3 (21.4%)	5 (17.9%)
	Other*	3 (21.4%)	7 (50.0%)	10 (35.7%)

*Other healthcare providers seen were: Oxygen supplier, care workers, community respiratory team, dentist, heart failure nurse, occupational therapy, outpatient appointment, outpatient x-ray, pharmacist, social worker. Some participants saw multiple other healthcare providers.

For peer review only

ACUTE pilot trial safety reporting procedures and adverse events

Flow chart detailing safety reporting procedures for the ACUTE study

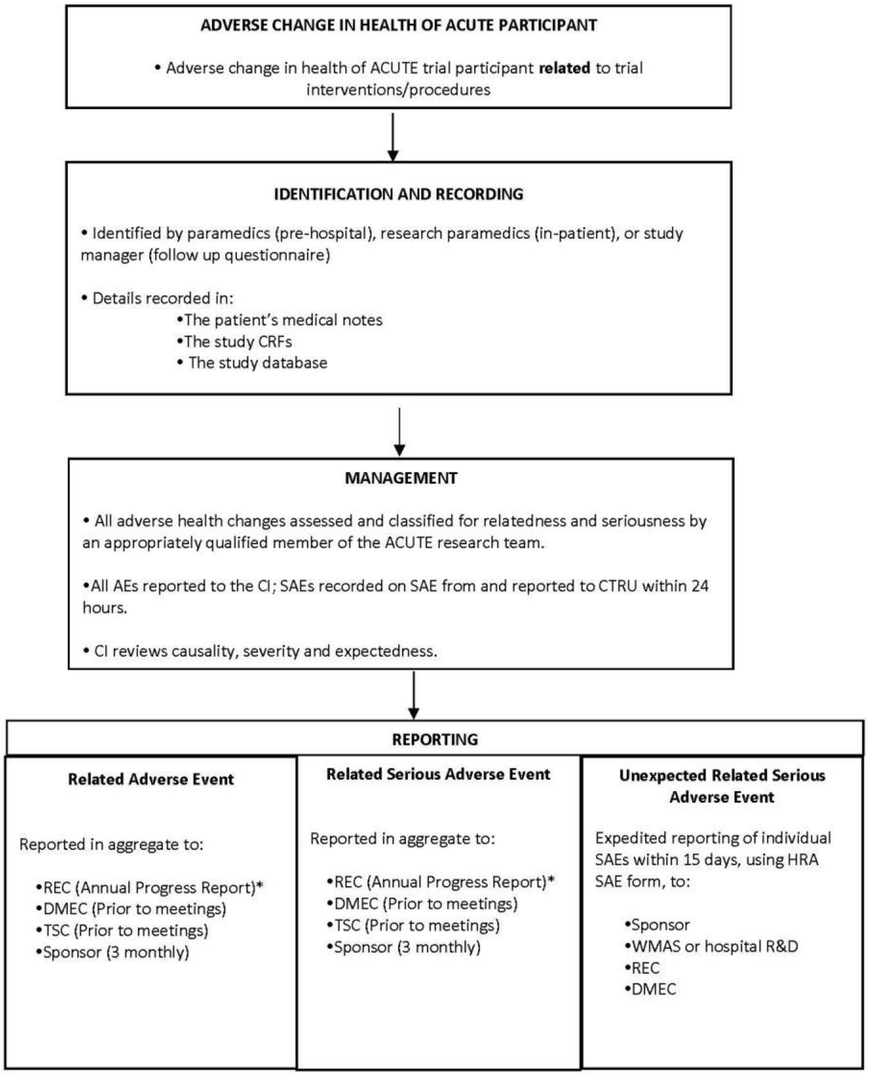

*Related AEs/SAEs resulting in treatment failure

Table of classification of adverse events.

Adverse Health Change Terminology*	Definition
**Seriousness**	
**Adverse Event (AE)**	An adverse change in health that occurs while a patient is taking part in a study
**Serious Adverse Event (SAE)**	Any adverse event occurring while a patient is taking part in a study, that results in:<br><br>• Death<br>• Life Threatening Illness<br>• Hospitalisation<br>• Prolongation of Hospitalisation<br>• Disability or Incapacity<br>• Congenital abnormality or birth defect<br>• Other adverse health change resulting in inability to perform routine activities or significant medical event
**Causality**	
**Unrelated**	An adverse change in health that occurs while a patient is taking part in a study which is not caused by or related to trial treatments.
**Related**	An adverse change in health that occurs while a patient is taking part in a study, which is caused by or related to trial treatments. An AE or SAE is considered related if the relationship between the event and trial treatments is:<br><br>• Possible - There is some evidence to suggest a causal relationship. However, the influence of other factors may have contributed to the event (e.g. the participant's clinical condition, other concomitant treatments).<br>• Probable - There is evidence to suggest a causal relationship and the influence of other factors is unlikely, or<br>• Definite - There is clear evidence to suggest a causal relationship and other possible contributing factors can be ruled out.
**Expectedness**	
**Unexpected**	Any adverse health change that is **NOT** consistent with the known and expected adverse events of trial treatments i.e. it is not listed in the protocol or related documents/literature as an expected occurrence.
**Expected**	Any adverse health change that **IS** consistent with the known and expected adverse events of trial treatments
**Severity***	
**Mild**	An adverse health change that does not interfere with routine activities
**Moderate**	An adverse health change that interferes with routine activities
**Severe**	An adverse health change that makes it impossible to perform routine activities

*The term 'severity', is used to describe the intensity, and should not be confused with 'serious' which is based on participant/event outcome or action criteria. For example, a headache may be severe but not serious, while a minor stroke is serious but may not be severe.

For peer review only

Table summary of the number of AEs and SAEs as well as the proportion of patients experiencing at least one AE or SAE for the ITT population

	CPAP	Standard Oxygen Therapy	Total
Patients	42	35	77
Number of AEs	27	20	47
Number of individuals with an AE	23 (54.8%)	17 (48.6%)	40 (51.9%)
Number of SAEs	22	17	39
Number of individuals with a SAE	19 (45.2%)	15 (42.9%)	34 (44.2%)

ACUTE pilot trial adverse events

Site	AE summary	AE category	Start date	End date	Serious?	Expected SAE?
Stoke	Headache	Other	19/08/2017	19/08/2017	No	No
Stoke	Arrested prior to receiving intervention	Cardiorespiratory arrest	13/09/2017	13/09/2017	Yes	No
Lichfield	Respiratory arrest	Other	17/09/2017	17/09/2017	Yes	No
Lichfield	Died prior to approach	Cardiorespiratory arrest	18/09/2017	18/09/2017	Yes	No
Stoke	Fractured NOF	Other	18/10/2017		Yes	No
Erdington	Social Care readmission	Other	23/10/2017	08/12/2017	Yes	No
Lichfield	CVA	Other	28/10/2017	15/12/2017	Yes	No
Stafford	Death	Cardiorespiratory arrest	29/10/2017	29/10/2017	Yes	Yes
Stafford	Death	Cardiorespiratory arrest	29/10/2017	29/10/2017	Yes	Yes
Stoke	Pt died	Cardiorespiratory arrest	16/11/2017	16/11/2017	Yes	No
Stoke	Re-admission within 30 days	Other	30/11/2017	30/11/2017	No	Yes
Stoke	Claustrophobia	Claustrophobia	12/12/2017	12/12/2017	No	Yes
Erdington	Distress at mask	Other	15/12/2017	15/12/2017	No	Yes

For peer review only

Erdington	Died within 30 days	Cardiorespiratory arrest	21/12/2017	21/12/2017	Yes	No
Stafford	Pt died	Cardiorespiratory arrest	29/12/2017	29/12/2017	Yes	Yes
Stoke	Mild claustrophobia	Claustrophobia	05/01/2018	05/01/2018	No	Yes
Erdington	Readmission to respiratory ward with pneumonia	Other	05/01/2018	15/01/2018	Yes	Yes
Stafford	Re-admission	Other	10/01/2018		Yes	No
Stoke	Death	Cardiorespiratory arrest	14/01/2018	14/01/2018	Yes	Yes
Stoke	Pt died	Cardiorespiratory arrest	17/01/2018	17/01/2018	Yes	Yes
Stafford	Pt died on day of recruitment	Cardiorespiratory arrest	23/01/2018	23/01/2018	Yes	Yes
Stafford	Died ahead of 30 day follow up	Cardiorespiratory arrest	27/01/2018	27/01/2018	Yes	Yes
Stafford	Died on elderly care ward	Cardiorespiratory arrest	28/01/2018	28/01/2018	Yes	Yes
Stoke	Agitation and distress with mask	Progressive respiratory distress	28/01/2018	28/01/2018	No	Yes
Erdington	Death	Cardiorespiratory arrest	06/02/2018	06/02/2018	Yes	Yes
Stafford	Patient died	Cardiorespiratory arrest	20/02/2018	20/02/2018	Yes	Yes
Lichfield	Died prior to 30 day	Cardiorespiratory arrest	03/03/2018	03/03/2018	Yes	Yes
Stafford	Pt re-admitted to hospital during 30-day period after recruitment	Other	08/03/2018	15/03/2018	Yes	Yes
Lichfield	Died prior to approach	Cardiorespiratory arrest	09/03/2018	09/03/2018	Yes	Yes
Erdington	Died following discharge prior to approach	Cardiorespiratory arrest	10/03/2018	10/03/2018	Yes	Yes
Lichfield	Died prior to approach	Cardiorespiratory arrest	17/03/2018	17/03/2018	Yes	Yes

For peer review only

Stoke	Pneumothorax	Tension pneumothorax	28/03/2018	28/03/2018	Yes	Yes
Stoke	Patient got worse	Vomiting	28/03/2018	28/03/2018	No	Yes
Stafford	Pt re-admitted, then died	Other	12/04/2018	15/04/2018	Yes	Yes
Lichfield	Mask wouldn't inflate	Other	17/04/2018	17/04/2018	No	No
Lichfield	Cardiac arrest	Cardiorespiratory arrest	08/05/2018	08/05/2018	Yes	Yes
Lichfield	Death	Cardiorespiratory arrest	31/05/2018	31/05/2018	Yes	Yes
Stafford	Died prior to approach	Cardiorespiratory arrest	03/06/2018	03/06/2018	Yes	Yes
Lichfield	Readmissions within 30 days	Other	30/06/2018	01/07/2018	Yes	Yes
Lichfield	Readmission within 30 days	Other	02/07/2018	06/07/2018	Yes	Yes
Lichfield	Claustrophobia and Progressive Respiratory Distress	Other	05/07/2018	05/07/2018	No	Yes
Lichfield	Pneumothorax	Other	05/07/2018	27/07/2018	Yes	Yes
Stafford	Re-admission	Other	06/07/2018		Yes	Yes
Lichfield	Readmission	Other	07/07/2018	27/07/2018	Yes	Yes
Stoke	Pt re-admitted during 30-day reference period	Other	25/07/2018	03/08/2018	Yes	Yes
Lichfield	Readmission	Other	28/07/2018		Yes	Yes
Stoke	Death	Cardiorespiratory arrest	22/08/2018	22/08/2018	Yes	Yes

For peer review only

BMJ Open

Is a Definitive Trial of Prehospital Continuous Positive Airway Pressure versus standard oxygen therapy for Acute Respiratory Failure Indicated? The ACUTE pilot randomised controlled trial

Journal:	*BMJ Open*
Manuscript ID	bmjopen-2019-035915.R3
Article Type:	Original research
Date Submitted by the Author:	01-Jun-2020
Complete List of Authors:	Fuller, Gordon; The University of Sheffield, School of Health and Related Research<br>Keating, Sam; The University of Sheffield, School of Health and Related Research<br>Goodacre, Steve; University of Sheffield, School of Health and Related Research<br>Herbert, Esther; The University of Sheffield, School of Health and Related Research<br>Perkins, Gavin; University of Warwick, Clinical Trials Unit<br>Rosser, Andy; West Midlands Ambulance Service NHS Foundation Trust<br>Gunson, Imogen; West Midlands Ambulance Service NHS Foundation Trust<br>Miller, Josh; West Midlands Ambulance Service NHS Foundation Trust<br>ward, matthew; West Midlands Ambulance Service NHS Foundation Trust<br>Bradburn, Mike; University of Sheffield, School of Health and Related Research<br>Thokala, Praveen; The University of Sheffield, School of Health and Related Research<br>Harris, Tim; Bart's Health/NHS, Emergency Medicine<br>Marsh, Maggie; Sheffield Emergency Care Forum<br>Scott, Alex; The University of Sheffield, School of Health and Related Research<br>Cooper, Cindy; University of Sheffield, Clinical Trials Research Unit
**Primary Subject Heading**:	Emergency medicine
Secondary Subject Heading:	Cardiovascular medicine, Respiratory medicine
Keywords:	Adult thoracic medicine < THORACIC MEDICINE, Adult intensive & critical care < INTENSIVE & CRITICAL CARE, ACCIDENT & EMERGENCY MEDICINE

SCHOLARONE™
Manuscripts

I, the Submitting Author has the right to grant and does grant on behalf of all authors of the Work (as defined in the below author licence), an exclusive licence and/or a non-exclusive licence for contributions from authors who are: i) UK Crown employees; ii) where BMJ has agreed a CC-BY licence shall apply, and/or iii) in accordance with the terms applicable for US Federal Government officers or employees acting as part of their official duties; on a worldwide, perpetual, irrevocable, royalty-free basis to BMJ Publishing Group Ltd ("BMJ") its licensees and where the relevant Journal is co-owned by BMJ to the co-owners of the Journal, to publish the Work in this journal and any other BMJ products and to exploit all rights, as set out in our licence.

The Submitting Author accepts and understands that any supply made under these terms is made by BMJ to the Submitting Author unless you are acting as an employee on behalf of your employer or a postgraduate student of an affiliated institution which is paying any applicable article publishing charge ("APC") for Open Access articles. Where the Submitting Author wishes to make the Work available on an Open Access basis (and intends to pay the relevant APC), the terms of reuse of such Open Access shall be governed by a Creative Commons licence – details of these licences and which Creative Commons licence will apply to this Work are set out in our licence referred to above.

Other than as permitted in any relevant BMJ Author's Self Archiving Policies, I confirm this Work has not been accepted for publication elsewhere, is not being considered for publication elsewhere and does not duplicate material already published. I confirm all authors consent to publication of this Work and authorise the granting of this licence.

For peer review only

Is a Definitive Trial of Prehospital Continuous Positive Airway Pressure versus standard oxygen therapy for Acute Respiratory Failure Indicated? The ACUTE pilot randomised controlled trial

Dr Gordon W Fuller[1]*, Dr Samuel Keating[2], Professor Steve Goodacre[1], Ms Esther Herbert[2], Professor Gavin D Perkins[3], Mr Andy Rosser[4], Ms Imogen Gunson[4], Mr Joshua Miller[4], Mr Matthew Ward[4], Mr Mike Bradburn[2], Dr Praveen Thokala[5], Professor Tim Harris[6], Ms Maggie Marsh[7], Dr Alex Scott[2], Professor Cindy Cooper[2]

Corresponding author*: Dr Gordon Ward Fuller

Work address: Centre for Urgent and Emergency Care Research, School of Health and Related Research, University of Sheffield, Regent Court, 30 Regent Street, Sheffield, S1 4DA, UK

Telephone: +44 (0) 7968 280641

E-mail: g.fuller@sheffield.ac.uk

1. Centre for Urgent and Emergency Care Research, School of Health and Related Research, University of Sheffield, Regent Court, 30 Regent Street, Sheffield, S1 4DA, UK

2. Clinical Trials and Research Unit, School of Health and Related Research, University of Sheffield, Regent Court, 30 Regent Street, Sheffield, S1 4DA, UK

3. Warwick Clinical Trials Unit, University of Warwick, Coventry, CV4 7AL, UK

4. West Midlands Ambulance Service, Trust Headquarters, Millennium Point, Waterfront Business Park, Waterfront Way, Brierley Hill, West Midlands, DY5 1LX, UK

5. Health Economics and Decision Science, School of Health and Related Research, University of Sheffield, Regent Court, 30 Regent Street, Sheffield, S1 4DA, UK

For peer review only

6. School of Medicine and Dentistry, Blizard Institute, Barts and The London School of Medicine and Dentistry, 4 Newark Street, London, E1 2AT, UK

7. Sheffield Emergency Care Forum, Clinical Research Office Sheffield, Royal Hallamshire Hospital, D Floor, Glossop Road, Sheffield, S10 2JF

Word count (excluding title page, abstract, references, figures and tables): 3,528 words

Key words: Continuous positive airway pressure (CPAP), acute respiratory failure, emergency medical services, pilot studies, randomised controlled trial

Contributions of authors:

The following drafted the manuscript: Gordon Fuller (Chief Investigator), Steve Goodacre (Co-chief Investigator), Sam Keating (Trial Manager), Esther Herbert (Statistician), Mike Bradburn (Co-applicant, statistician), Praveen Thokala (Co-applicant, health economist), Josh Miller (Research paramedic), Maggie Marsh (Co-applicant, PPI representative).

The following conceived of, or designed, the work: GF, SG, SM, PT, MM, MB, Gavin D Perkins (Co-applicant), Matthew Ward (Co-applicant), Tim Harris (Co-applicant), MM, Alex Scott (Qualitative researcher), Cindy Cooper Co-applicant (CTRU Director).

The following were involved in the acquisition of data for the work: Andy Rosser (Lead Research Paramedic), Matthew Ward (Co-applicant), Imogen Gunson (Research paramedic), Josh Miller (Research paramedic)

The following were involved in the analysis and the interpretation of data for the work: GF, SG, SK, EH, MB, PT, JM, IG, MM, AS

The following contributed to the management and conduct of the trial: SK, Sarah Gonzalez (Trial support officer), AR, MW

All authors revised the work critically for important intellectual content and were involved in the final approval of the version to be published.

All authors agree to be accountable for all aspects of the work in ensuring that questions related to the accuracy or integrity of any part of the work are appropriately investigated and resolved.

For peer review only

Data Sharing Statement:

All data requests should be submitted to the corresponding author for consideration. Access to anonymised data may be granted following review.

Competing interests: Professor Steve Goodacre is Deputy Director of the NIHR HTA Programme, Chair of the NIHR HTA Commissioning Board and member of the NIHR HTA Funding Strategy Group. Professor Gavin Perkins is a NIHR Senior Investigator and member of the Programme Grants for Applied Research Board. Professor Cindy Cooper is a member of the NIHR Clinical Trials Unit Standing Advisory Committee and of the UK Clinical Research Collaboration Registered Clinical Trials Unit Network Executive Group. All authors have completed the unified competing interest form at www.icmje.org/coi_disclosure.pdf (available on request from the corresponding author) and declare (1) no financial support for the submitted work from anyone other than their employer; (2) no financial relationships with commercial entities that might have an interest in the submitted work; (3) no spouses, partners, or children with relationships with commercial entities that might have an interest in the submitted work; and (4) no non-financial interests that may be relevant to the submitted work.

Funding statement

This work was supported by the National Institute for Health Research's Health Technology Assessment Programme, grant number HTA15/08/40. The funder, and CPAP manufacturer and supplier, were not involved in study design, conduct or publication.

For peer review only

ABSTRACT

Objectives: To determine the feasibility of a large-scale definitive multicentre trial of prehospital continuous positive airway pressure (CPAP) in acute respiratory failure (ARF).

Design: A single centre, open label, individual patient randomised, controlled, external pilot trial.

Setting: A single UK Ambulance Service, between August 2017 and July 2018.

Participants: Adults with respiratory distress and peripheral oxygen saturations below British Thoracic Society (BTS) target levels despite controlled oxygen treatment.

Interventions: Patients were randomised to prehospital CPAP (O-Two system) versus standard oxygen therapy in a 1:1 ratio using simple randomisation.

Primary and secondary outcome measures: Feasibility outcomes comprised recruitment rate, adherence to allocated treatment, retention and data completeness. The primary clinical outcome was 30 day mortality.

Results: 77 patients were enrolled (target 120), including 7 cases with a diagnosis where CPAP could be ineffective or harmful. CPAP was fully delivered in 74% (target 75%). There were no major protocol violations. Full data were available for all key outcomes (targets ≥90%). Overall 30 day mortality was 27.3%. Of these deceased patients, 14/21 (68%) either did not have a respiratory condition or had ceiling of treatment decisions implemented excluding hospital non-invasive ventilation and critical care.

Conclusions: Recruitment rate was below target and feasibility was not demonstrated. Limited compliance with CPAP, and difficulty in identifying patients who could benefit from CPAP, indicate that prehospital CPAP is unlikely to materially reduce mortality. A definitive effectiveness trial of CPAP is therefore not recommended.

Trial registration: ISRCTN12048261

For peer review only

Strengths and limitations of this study

- This study provides important information regarding the feasibility of a large-scale definitive randomised controlled trial investigating prehospital CPAP in acute respiratory failure.
- Expert recommendations for pilot randomised controlled trials were followed.
- A pragmatic study design was used to maximise generalisability to real-life prehospital practice.
- A novel allocation concealment method using identical sealed boxes was used.
- Patients and clinicians were not blinded to the intervention.

For peer review only

BACKGROUND

Acute respiratory failure (ARF) is a common medical emergency caused by cardiac or respiratory diseases, including heart failure, pneumonia, chronic obstructive pulmonary disease (COPD) and asthma.[1] The incidence of ARF has been estimated at 80 cases per 100,000 per year in the United States.[1] Although varying according to the underlying cause, the overall risk of death is high, with estimates of 30 day mortality ranging between 12% and 20%.[2] ARF has substantial health services costs, with patients often requiring prolonged hospital stays, ventilatory support and critical care admissions.[3, 4] ARF is responsible for over 3 million National Health Service (NHS) bed days and hospital costs of £9.6 million per year in England.[5,6]

Current prehospital management of ARF in the NHS uses a standard management approach of controlled oxygen therapy; supplemented by specific ancillary treatments directed at the underlying disease.[7] This therapy is usually delivered by ambulance service personnel without physician support. Continuous positive airways pressure (CPAP) is widely used in hospital to treat acute respiratory failure due to COPD, pneumonia, decompensated obstructive sleep apnoea, cardiogenic pulmonary oedema, and chest wall trauma;[8] and it has been suggested that it may be more effective if delivered earlier i.e. en route to hospital.[5] The difficulties of prehospital diagnosis mean that prehospital CPAP is likely to be applied generally to all cases of ARF, rather than directed towards those due to a specific cause.

Existing research investigating prehospital CPAP is inconclusive. A recent evidence synthesis suggested that prehospital CPAP may reduce the risk of mortality in ARF compared to standard treatment, but noted that the primary studies were relatively small, heterogeneous, at risk of bias, and were provided by specialist services, so may not be applicable to routine prehospital practice.[5] These findings suggest that although prehospital CPAP is a promising and potentially efficacious therapy, a large pragmatic trial is needed to confirm effectiveness when implemented as part of routine practice across a clinically relevant population with ARF.

Prior to a large pragmatic trial and economic evaluation, it is first necessary to estimate the incidence of eligible patients to determine whether a trial would be feasible and cost-effective. It is also important to determine whether prehospital CPAP can be delivered successfully in the context of the NHS ambulance services. For these reasons a stand-alone feasibility study is necessary to estimate the incidence of eligible patients, test the feasibility and acceptability of potential definitive trial methods, and address important uncertainties, such as patient selection, delivery of the intervention and event rates.

For peer review only

METHODS

Study design, aims and objectives

The ACUTE (Ambulance CPAP: Use, Treatment effect and Economics) study was an open label, individual randomised, pragmatic, parallel group, external pilot trial. It aimed to determine the feasibility of a definitive trial evaluating the effect of prehospital CPAP (intervention arm) compared to standard oxygen therapy (control arm) on 30-day mortality from ARF. The feasibility and effectiveness outcomes are listed in Table 1. The trial was registered prior to commencement with the International Standard Randomised Controlled Trials Number Registry (ISRCTN12048261) and a protocol, with pre-planned outcomes and analysis plan, has been previously published.[9] Health economic results will be reported separately.

Setting and study population

Recruitment took place across four hubs covering 1.5 million people in an English ambulance service between 1st August 2017 and August 2018 (last follow up completed 13th August 2018). Adults with respiratory distress and peripheral oxygen saturation below British Thoracic Society (BTS) target levels (88% for patients with COPD, 94% for other conditions) despite supplemental oxygen (titrated low flow oxygen for COPD, or titrated high flow oxygen in other conditions) were eligible.[10] Patients with pre-existing lack of capacity; or with limited potential to benefit from, or contraindications to CPAP, were excluded. Inclusion and exclusion criteria are detailed in Table 2 and were based on clinician judgement at the scene of incident.

For peer review only

Table 1. ACUTE trial objectives and outcomes

**Feasibility outcomes**	**Feasibility targets**
1. Rate of eligible patients per 100,000 population per year	•Target 8 per 100,000 per year i.e. 120 across the 1.5 million population of the 4 hubs
2. Proportion recruited and allocated to treatment appropriately	•Proportion recruited in error and classified as major or minor non-compliances (target 0% and ≤10%).
3. Adherence to allocated treatment	•Adherence to the allocation schedule (target ≥90%)<br>•Adherence to treatment in the CPAP arm (target ≥75%)
4. Retention and data completeness up to 30 days	•Retention at 30 days (target ≥90%)<br>•Data completeness (target ≥90%)
**Clinical outcome measures:**	
1. Proportion surviving to 30 days (primary outcome for definitive trial)	
2. Proportion undergoing endotracheal intubation by 30 days	
3. Proportion admitted to critical care at any point up to 30 days	
4. Mean and median length of hospital stay	
5. Change in Visual Analogue Scale (VAS) dyspnoea score from presentation to immediately before ED arrival	
6. Mean change in quality of life, measured with EQ-5D-5L[6]	
7. Key elements of post-discharge health-care resource use up to 30 days	

For peer review only

Table 2. ACUTE trial eligibility criteria

Inclusion criteria

ALL of:

- Adults ≥ 18 years
- Respiratory distress ,
- Hypoxia:
  - COPD: Sats <88% despite low flow oxygen
  - Other conditions: Sats <94% despite high flow oxygen.

Exclusion criteria

ANY of:

1. Hospital CPAP treatment available within 15 minutes of eligibility assessment.
2. Age < 18 years
3. Known to have terminal illness
4. Known pre-existing lack of capacity (confirmed by relatives, carers or documentary evidence, such as Lasting Power of Attorney)
5. Documented not for resuscitation status
6. Acutely incapacitated patients with known valid advanced directive declining non-invasive ventilation or participation in research
7. The patient has an oxygen alert card*
8. Anticipated inability to apply CPAP (e.g. facial deformity)
9. Respiratory failure due to chest trauma
10. Contraindication to CPAP (suspected pneumothorax, respiratory arrest, epistaxis, vomiting, hypotension)
11. Previous enrolment in the ACUTE trial
12. Pregnancy
13. Patients unable to communicate with ambulance service clinicians

*Patient-held card warning against use of high flow oxygen. Provided to patients with a history of previous hypercapnic acidosis with a PaO_2 >10.0 kPa—indicating that oxygen may have worsened the hypercapnia.

For peer review only

Randomisation

Enrolled patients were individually allocated to CPAP or standard oxygen therapy in a 1:1 ratio using simple randomisation. The randomisation sequence was computer generated by an independent statistician not directly involved in the conduct of the trial. The allocation schedule was held centrally on a password-protected, access-restricted network drive. The trial statistician did not have access to the randomisation sequence until after data lock.

Participant enrolment and consent

Ambulance service clinicians (paramedics and ambulance technicians) volunteering to participate in the ACUTE study and trained in trial procedures identified potential participants with ARF when attending emergency 999 ambulance calls. In patients judged to have capacity, verbal consent was obtained for participation. Eligible patients lacking mental capacity were enrolled in the trial without consent if determined to be in their best interests. In all cases, a research paramedic reviewed the participant in hospital as soon as possible after enrolment, obtaining consent for further data collection and participation in the trial according to the provisions of the Mental Capacity Act 2005. [11,12] In the contingency that consent was declined, prehospital data was retained and anonymised 30 day mortality data collected. If a patient died before approach for written consent, ethical approval allowed for collection of anonymised prehospital, hospital and 30 day mortality data.

Allocation concealment and blinding

CPAP devices and high concentration oxygen therapy masks were packaged in identically appearing, numbered, shrink wrapped, tamper proof sealed, trial equipment boxes. Equipment boxes were independently assembled in an audited process in accordance with the randomisation sequence at Sheffield Clinical Trials Research Unit. Boxes were supplied to participating ambulance hubs, and held unordered by number, in a secure designated storage area. Boxes were subsequently signed in and out for each shift by participating clinicians. Research paramedics audited the location and condition of all boxes, and adherence to the allocation schedule on a weekly basis. Due to the physical differences between the CPAP device and standard oxygen mask it was not possible to subsequently blind patients, ambulance service clinicians, hospital clinicians, or outcome assessors to the treatment arms.[9]

For peer review only

Trial treatments

Immediately after enrolment, paramedics opened the trial equipment box and provided treatment according to whether a CPAP device or high concentration oxygen mask was supplied. Patients in the intervention arm were treated with the O-Two unit (O-Two Medical Technologies, Ontario, Canada; supplied by SP Services(UK), Telford, UK), a lightweight, portable, open, single use, low flow CPAP system.[7] CPAP was started at 5 cm H2O and then incrementally increased by 1 cm H2O every 2–5 min to a maximum of 15 cm H2O. Patients in the control arm received standard oxygen therapy using nasal cannula, an air entrainment Venturi mask, a simple face mask, or a non-rebreathing reservoir face mask. The choice of flow rate and oxygen delivery device was titrated by ambulance service clinicians according to the patient's condition, and peripheral oxygen saturation levels.

Treatment in both arms was targeted to BTS guidelines for peripheral oxygen saturations.[8] Target peripheral oxygen saturations were 88-92% for patients with known/suspected COPD and 94-98% for patients with other suspected causes of acute respiratory failure. Ancillary condition-specific treatments were administered in both trial arms according to Joint Royal Colleges Ambulance Liaison Committee (JRCALC) Clinical Practice Guidelines.[7] Subsequent hospital management, including emergency department CPAP or non-invasive ventilation, was at the discretion of the hospital clinician.

Data collection and safety reporting

Electronic patient records and computer aided dispatch data were screened by research paramedics to identify potentially eligible, but unenrolled, patients. Routinely collected prehospital data (e.g. timings, vital signs) were collated from participants' electronic records by research paramedics. Additional trial specific information was collected using a trial form completed by recruiting ambulance service clinicians and contained within each equipment box. At 30 days, research paramedics reviewed the hospital records to collate details of subsequent progress, treatments, and vital status at 30 days. Quality of life and post-discharge resource use was assessed by questionnaire at 30 days following enrolment, either in person if still in hospital; or by telephone or post if discharged. Adverse health changes in participants were defined, monitored, recorded and reported according to UK Health Research Authority guidance.[13]

For peer review only

Sample size and statistical analyses

The baseline characteristics of enrolled patients are reported descriptively for the whole trial population, and separately per treatment arm. Feasibility outcomes are reported descriptively for the whole trial population, together with their 95% confidence interval. Summary estimates of relative and absolute effectiveness outcomes are presented, overall and stratified by treatment arm, with 95% confidence intervals. An intention to treat analysis was used, using either a full analysis set, or a complete case analysis for endpoints with missing outcome data.[14]

The ACUTE feasibility study aimed to recruit 120 patients over 12 months, the minimum number recommended to estimate binary parameters for the sample size calculation of the full trial.[15] Mortality under standard care was estimated at 12% and for the full trial a 5% absolute reduction was postulated (i.e. to 7%) in the intervention arm.[1, 3, 4] This sample size would allow estimation of mortality to within a standard error of 2.7% for use in the sample size calculation for any definitive trial; and feasibility outcomes to be estimated with a precision of <5%. Achieving this target would also indicate a recruitment rate sufficient to deliver the sample size for a definitive trial; and an incidence of ARF necessary to deliver a potentially cost-effective prehospital CPAP service.

All analyses were conducted in the R statistical package (R Foundation for Statistical Computing, Vienna, Austria) in accordance with a pre-specified statistical analysis plan and CONSORT principles. Following review of blinded outcome data during trial management group meetings an unexpectedly high overall mortality rate was noted. A post-hoc descriptive analysis of the deceased patients was therefore conducted.

Trial oversight, ethics and governance

The trial received ethical approval from the NHS Leeds East Research Ethics Committee (Reference: 16/YH/0406). A protocol amendment from permuted block to simple randomisation was approved prior to recruitment (16th February 2017, REC reference: 16/YH/0406). This work was supported by the National Institute for Health Research's Health Technology Assessment Programme, grant number HTA15/08/40. The funder, and CPAP manufacturer and supplier, were not involved in study design, conduct or publication.

For peer review only

Public and Patient Involvement

The public and patients were fully involved in the ACUTE study from conception to dissemination. The research proposal was developed in partnership with a service user co-applicant and patient advisory groups. A service user advisory group was enlisted for collaboration throughout the project; and a lay co-applicant provided advice on trial matters, attended TMG meetings, supported study management, and contributed to the study report and interpretation of results.

For peer review only

RESULTS

Recruitment and consent

Over the recruitment period, 364 patients with ARF meeting ACUTE trial eligibility criteria presented from the 1.5 million population of four participating ambulance service hubs. 161 of those patients (44.2%) were attended by ACUTE trained ambulance service clinicians and could potentially have been recruited. Of these 161, 77 (47.8%) were enrolled in the trial. Slightly more participants were randomised to the CPAP intervention arm (42 cases), than to the standard oxygen control arm (35 cases). Consent for further data collection was declined by 9 patients (11.7%) and 17 patients (22.1%) died prior to research paramedic approach for consent. Figure 1 presents the participant flow for the trial.

Baseline characteristics

The trial population was relatively elderly (median age 71 years), predominantly male (62.3%), and severely unwell (median VAS breathlessness score 9/10, median pulse 115 beats/minute, median respiratory rate 34 breaths/minute, and median initial peripheral oxygen saturations of 78.5%). The most common final hospital diagnoses were COPD (n=21/65, 32.3%) and lower respiratory tract infection (LRTI, n=28/65, 43.1%). A minority of cases (n=4/65, 6.2%) had a non-respiratory primary hospital diagnosis comprising abdominal aortic aneurysm, myocardial infarction, sepsis (not further specified), and liver failure (ascites). Pre-randomisation characteristics were similar across trial arms as summarised in Table 3.

For peer review only

[Figure 1 here]

For peer review only

Figure 1. CONSORT diagram of participants' flow through the ACUTE pilot trial

Table 3. Baseline characteristics of ACUTE trial participants by arm

Baseline Variable	Descriptive statistic	CPAP<br>N = 42	Standard Oxygen Therapy<br>N = 35	Total<br>N = 77
Age	Median (IQR)	70.0 (60.5, 76.8)	73.0 (65.0, 77.0)	71.0 (62.0, 77.0)
Sex	Male	27 (64.3%)	21 (60.0%)	48 (62.3%)
Hospital ARF diagnosis*	n=	36	30	66
	Asthma	1 (2.8%)	1 (2.9%)	2 (3.0%)
	COPD	10 (27.8%)	11 (36.7%)	21 (31.8%)
	Heart Failure	4 (11.1%)	2 (6.7%)	6 (9.1%)
	LRTI	17 (47.2%)	11 (36.7%)	28 (42.4%)
	Pulmonary embolism	1 (2.8%)	0 (0.0%)	1 (1.5%)
	Other	3 (8.3%)	4 (13.3%)	7 (10.8%)
Breathlessness at enrolment (VAS 0-10)	n=	41	35	76
	Median (IQR)	9.0 (8.0, 10.0)	9.0 (8.0, 9.5)	9.0 (8.0, 10.0)
First systolic blood pressure (mmHg)	n=	40	30	70
	Median (IQR)	136.0 (115.2, 150.5)	126.5 (112.0, 152.0)	134.5 (112.2, 152.0)
First Glasgow Coma Score	n=	42	35	77
	Median (IQR)	15.0 (14.0, 15.0)	15 (14.5, 15.0)	15 (14.0, 15.0)
First oxygen saturations (%)	n=	41	35	76
	Median (IQR)	78 (74.0, 85.0)	82 (75.5, 86.0)	78.5 (74.8, 86.0)
First pulse rate (bpm)	n=	42	33	75
	Median (IQR)	117.0 (105.0, 125.8)	111 (92.0, 121.0)	115 (100.0, 124.0)
First Respiratory rate (breaths/min)	n=	42	35	77
	Median (IQR)	36.0 (30.5, 40.0)	32 (24.0, 40.0)	34 (28.0, 40.0)
Duration between arrival at scene and	Median (IQR)	43.0 (34.0, 49.8)	36.0 (32.5, 46.5)	40.00 (34.0, 49.0)

For peer review only

Baseline Variable	Descriptive statistic	CPAP<br>N = 42	Standard Oxygen Therapy<br>N = 35	Total<br>N = 77
departure to hospital (mins)				
Duration between leaving the scene and arriving at hospital (mins)	Median (IQR)	13.0 (9.00, 18.75)	15.0 (10.0, 20.5)	13.0 (10.0, 20.0)

*Consent was declined for data collection in 9 cases, clinical records were unavailable in 2 cases, and in 1 case there was no clear underlying diagnosis apparent in the notes.
IQR: Interquartile range; bpm: beats per minute; COPD: Chronic Obstructive Pulmonary Disease; LRTI: Lower Respiratory Tract Infection.

For peer review only

Feasibility outcomes

Feasibility outcomes, compared to the pre-specified target, are summarised in Table 4. The sample size of 77 enrolled patients resulted in a recruitment rate of 5.1 (95% CI 4.1-6.4) per 100,000 population per year; falling short of the feasibility target of 8 per 100,000 population per year (i.e. 120 patients).

All patients were recruited appropriately with no major or minor protocol non-compliances (targets of ≤10% and 0% respectively). There was full adherence to the allocation schedule (feasibility target ≥90%). Treatment with CPAP was attempted in all patients enrolled in the intervention arm. All patients received appropriate standard oxygen management in the control arm, although one patient was enrolled who used their own CPAP machine.

CPAP was fully delivered as planned (i.e. administered until hospital arrival, or discontinued due to patient improvement after successful treatment) in 73.8% (31/42, target 75%) of intervention arm patients. CPAP was commenced in 90.5% of patients (38/42), with two patients refusing to wear the mask, one patient spontaneously improving and a fourth patient having a cardiac arrest prior to commencement. Of patients commencing CPAP, 31 (81.6%) continued with CPAP until they arrived at hospital: six did not tolerate CPAP and the remaining patient (with a final diagnosis of pulmonary fibrosis) was transferred to a standard oxygen non-rebreather mask due to non-improvement.

Full data were available for key outcomes, including all feasibility endpoints and vital status at 30 days, compared to the feasibility targets of ≥90% retention at 30 days and ≥90% data completeness. A small amount (<1%) of prehospital data describing baseline patient characteristics was not available due to missing values. A larger proportion of hospital data was missing secondary to lack of consent for collection (n=9, 11.7%), absence of local research approvals to access hospital data (n=2, 2.6%), or unclear or absent information in the hospital clinical records (differing across variables, ranging from n=0, 0%, for hospital length of stay to n=25, 32.5%, for emergency department management). Of patients alive at 30 days (n=56), 30 day follow up questionnaires examining quality of life and post-discharge health resource use, were fully completed by 40 patients (71.4%).

For peer review only - http://bmjopen.bmj.com/site/about/guidelines.xhtml

Table 4. Summary of feasibility results

Feasibility Outcome	Target	Result
Recruitment rate	8 per 100,000 population per year (i.e. 120 patients recruited	• 5.1 (95% CI 4.1-6.4) per 100,000 population per year<br>• 77 enrolled patients.
Major and minor non-compliances	0% and ≤10%	• 0% - major non-compliances<br>• 1.3% - minor non-compliance
Adherence to the allocation schedule	Target ≥90%	•100% adherence to allocation schedule
Adherence to treatment in the CPAP arm	Target ≥75%	• 74% -CPAP fully delivered as planned
Retention at 30 days	Target ≥90%	•100% - follow up for all feasibility endpoints and 30 day mortality
Data completeness	Target ≥90%	Data completeness for outcomes:<br>• 100% - feasibility outcomes<br>• 100% - 30 day mortality<br>• 81% - 30 day intubation<br>• 84% - admission to critical care<br>• 99% - clinician assessed breathlessness<br>• 86% - length of hospital stay<br>• 85% - baseline EQ-5D-5L*<br>• 71% - 30 day EQ-5D-5L*<br>• 73% - 30 day resource use*

*Of alive patients

For peer review only

Effectiveness and safety outcomes

Overall mortality of the study population was higher than expected with 27.3% (n=21/77) patients dying by 30 days. 28.6% (n=12/42) patients died by 30 days in the CPAP arm and 25.7% (n=9/35) in the standard oxygen arm (expected 12%). The absolute risk difference of mortality in CPAP group compared to standard oxygen therapy was 2.9% (95% CI -19.7-25.4%). An unplanned descriptive analysis of the deceased patients was undertaken to explore the circumstances for the unexpectedly high mortality rate. Data were not available for two cases due to lack of research approvals to access data, and two patients died from non-cardiorespiratory conditions not amenable to NIV (ruptured abdominal aneurysm and liver failure). Of the remaining 17 cases, 6 patients (35.3%) received hospital NIV (n=5/17, 29.4%) or mechanical ventilation (n=1, 5.9%). The other 11 patients had hospital ceiling of treatment decisions excluding hospital NIV and critical care management (11/17, 64/7%). Other secondary effectiveness outcomes are summarised in Table 5, and were similar across trial arms. Post-discharge resource use is summarized in supplementary file 1.

In total, 39 expected related serious adverse events (SAE) occurred in 34 patients (45.2% for the CPAP arm, 42.9% for the standard oxygen therapy arm). The majority were deaths (20/39, 51.2%) or re-admission to hospital within 30 days (13/39, 33.3%). There were no unexpected related SAEs. Two patients (1 intervention arm patient not receiving CPAP and 1 control arm patient) were categorised with related expected serious adverse events following diagnosis with pneumothoraces requiring intercostal drainage after hospital admission. There were no significant SAEs attributable to CPAP therapy. Adverse events are detailed in supplementary file 2.

For peer review only

Table 5. Summary of effectiveness outcomes

**Effectiveness outcome**	**CPAP**<br><br>**N=42**	**Standard Oxygen Therapy**<br>**N=35**	**Total**<br><br>**N=77**	**Absolute risk difference (95% CI)**
30 day mortality	n = 42<br>12<br>(28.6%)	n = 35<br>9<br>(25.7%)	n = 77<br>21<br>(27.3%)	2.9%<br>(-19.7 - 25.4)
Intubated	n = 33<br>2<br>(6.1%)	n = 29<br>1<br>(3.4%)	n = 62<br>3<br>(4.8%)	2.6%<br>(-10.5 - 15.7)
Admission to critical care	n = 35<br>4<br>(11.4%)	n = 30<br>2<br>(6.5%)	n = 65<br>6<br>(9.2%)	4.8%<br>(-12.1 – 21.7)
				Median of the difference (95% CI)
Median length of stay (days, IQR)	n = 22<br>10.0<br>(6.5, 12)	n = 22<br>7.0<br>(5, 9.8)	n = 44<br>8<br>(5.5, 11.2)	3.00<br>(-0.00, 6.00)
Change in patient reported breathlessness over prehospital interval (VAS, IQR)	n = 18<br>-3<br>(-4, -2)	n = 18<br>-2<br>(-4, -1)	n = 36<br>-2.5<br>(-4, -1)	-2.00<br>(-3.00, 0.00)
Change in clinician assessed breathlessness over prehospital interval (VAS, IQR)	n = 41<br>-3<br>(-5, -1)	n = 35<br>-2<br>(-3.5, 1)	n = 76<br>-2<br>(-4, -1)	-1<br>(-2, 0)
30 day EQ-5D-5L (IQR)	n = 22<br>0.82<br>(0.58, 0.95)	n = 18<br>0.73<br>(0.43, 0.89)	n = 40<br>0.76<br>(0.48, 0.92)	0.08<br>(-0.6, 0.26)
Median change in EQ-5D-5L (IQR)	n = 22<br>0.09<br>(-0.01, 0.16)	n = 18<br>0.10<br>(-0.06, 0.19)	n = 40<br>0.09<br>(-0.02, 0.18)	0.0 (-0.12, 0.16)

IQR: interquartile range; VAS: Visual analogue scale; EQ-5D-5L: EuroQOL-5D-5L Value Sets

For peer review only

DISCUSSION

Summary of results

Over 12 months 77 patients were enrolled, below the recruitment target of 120 participants. CPAP was fully delivered as planned in 74% of intervention arm patients (target 75%). There were no major protocol violations. Full data were available for key outcomes, including all feasibility endpoints and vital status at 30 days. Mortality was higher than expected, with 27.3% of patients dying by 30 days. Of the deceased patients 68% either did not have a respiratory condition or had explicit or implicit ceiling of treatment decisions excluding hospital NIV or critical care. Two patients, neither receiving CPAP, were diagnosed with a pneumothorax in the emergency department and were reported as expected related serious adverse events.

Interpretation

These findings suggest that pilot study methods could be used for a future definitive trial. Patients were appropriately recruited using a deferred consent model, there were no protocol violations, adherence to the allocation schedule was complete, and full data were available for key outcomes. The ACUTE pilot trial demonstrated a recruitment rate that was below the target rate considered a priori necessary to deliver a definitive pragmatic trial. However, the majority of potentially eligible cases presented to non-trial trained staff and it is therefore possible that recruitment could be improved if a full trial mandated participation of all ambulance service personnel.

External pilot trials are not designed or powered to generate estimates of clinical effect that should be used for decision-making.[16-18] We therefore draw no conclusions from comparisons of outcomes between CPAP and standard care. However, a number of findings from the ACUTE pilot trial can be used to inform a judgement regarding whether it would be plausible for a large trial to detect an effect from CPAP on mortality and therefore whether a definitive trial might be worthwhile.

Firstly, it is apparent that prehospital identification of patients with the potential to benefit from CPAP may be challenging. A small, but significant minority of cases were ultimately diagnosed with conditions where CPAP could not conceivably be beneficial.[8, 19] It is also concerning that pneumothoraces requiring intercostal drainage were detected in 2 cases. Although neither patient received prehospital CPAP, the potential for iatrogenic harm is conspicuous.

For peer review only

Secondly, delivery of CPAP was relatively limited, with 74% of intervention arm patients continuing treatment to hospital as planned. Although CPAP may be efficacious, the potential to demonstrate effectiveness would be restricted by lack of treatment compliance.

Thirdly, a key rationale for the implementation of prehospital CPAP is that earlier instigation of treatment will improve outcomes over and above the availability of hospital NIV.[5] In the pilot trial, relatively short on-scene times and conveyance times were recorded (median of 40 minutes and 13 minutes respectively). The potential time advantage from prehospital administration of CPAP may be too small to produce meaningful benefit.

Finally, illness severity was much higher than anticipated. The increased overall 30 day mortality risk of 27.3% might initially appear to offer a definitive trial a greater opportunity to detect a clinically relevant survival benefit. However, many enrolled patients had apparent treatment limitation decisions for ward level hospital care only, indicating a study population with a high prevalence of end-stage cardiorespiratory disease, multiple severe comorbidities, or very poor pre-morbid performance status where CPAP treatment might be futile and overly burdensome. However, it should be noted that CPAP could also affect important patient centred outcomes such as symptom relief, which might be detected by differences in dyspnoea VAS scores.

In summary, although the higher than expected mortality rate might suggest increased potential to detect an absolute difference in mortality, the challenges of providing prehospital CPAP, and characteristics of the patients who would benefit from CPAP, suggest limited potential to improve survival. It therefore appears unlikely that a trial powered to detect a plausible effect size could be designed.

Limitations

The purpose of a feasibility study is to determine if a large-scale trial can be performed.[18] Therefore, challenges that might be interpreted as weaknesses when appraising a definitive RCT (such as low rates of recruitment and adherence) actually represent important learning points in the feasibility setting. Modification of eligibility criteria to specify the presence of a primary cardio-respiratory diagnosis as an inclusion criteria, and exclusion of patients with home CPAP machines, could select a more appropriate study population. The use of retrospective case note review for hospital data collection improved the efficiency of data collection, but is associated with well recognised limitations that could have resulted

For peer review only

in inaccurate measurement of study endpoints. Furthermore, collection of more detailed data on NIV treatment in the emergency department would be beneficial in any future trial.

Generalisability

Trial eligibility criteria were broad, excluding only patients where CPAP was contraindicated (e.g. vomiting), where entry would likely not be appropriate (e.g. Do not resuscitate status, pre-existing loss of capacity), or where verbal consent was not possible (e.g. language barrier). The clinical findings reported herein should therefore have strong generalisability to patients presenting with ARF in NHS ambulance services. External validity to other settings, with different demographics or prehospital systems, is less clear. For example, international emergency medical services may have shorter on-scene times, use CPAP in less severe ARF, or use prehospital physicians with ultrasound skills to detect pneumothorax before CPAP application. Furthermore, a novel CPAP device was used. Although there are considerable advantages to the studied O-Two unit including small size, low cost and simplicity, there may also be limitations compared to other more complex prehospital CPAP systems e.g. FiO2 and CPAP level are jointly determined by the oxygen flow rate. Consequently, efficacy could differ with other methods of delivering prehospital CPAP.

Comparison to previous literature

A series of recent observational studies have also demonstrated that CPAP can be implemented by EMS.[20-24] These studies are consistent with the ACUTE experience of sub-maximal CPAP adherence and difficult prehospital diagnosis, including treatment of patients with pneumothoraces. A recent systematic review identified 10 trials and quasi-randomised studies comparing prehospital NIV (including CPAP) with standard oxygen therapy.[5] Network meta-analysis suggested that prehospital CPAP is an effective treatment for ARF, with evidence that it reduces mortality (odds ratio 0.41; 95% CrI 0.20 to 0.77) and intubation rate (0.32; 95% CrI 0.17 to 0.62) compared to standard care.2 However, some included studies were at risk of selection bias from lack of allocation concealment and information bias, secondary to unblinded outcome assessment. Furthermore, the meta-analysis findings have doubtful external validity to prehospital practice in many settings. Only one trial included undifferentiated respiratory failure patients, and the methods used to deliver prehospital CPAP (physician or paramedics with online physician support) are not routine in many prehospital care systems, reflected in higher levels of CPAP compliance.[5]

Conclusions

For peer review only

Pilot trial recruitment rate was below the target rate. Limited compliance with CPAP, and a study population including patients who could not benefit from CPAP, suggest that a clinically significant effect size is not plausible. A definitive effectiveness trial of CPAP is therefore not recommended. These findings also argue against routine implementation of CPAP into many ambulance services; but would not preclude a CPAP service provided by clinicians with extended training (e.g. prehospital physicians) where advanced clinical skills might allow selective targeting of treatment to an appropriate subgroup of patients.

For peer review only

REFERENCES

1. Creagh-Brown B. Respiratory failure. Medicine 2016;**44**(6):342-45

2. Stefan MS, Shieh MS, Pekow PS, et al. Epidemiology and outcomes of acute respiratory failure in the United States, 2001 to 2009: a national survey. J. Hosp. Med. 2013;**8**(2):76-82

3. Ray P, Birolleau S, Lefort Y, et al. Acute respiratory failure in the elderly: etiology, emergency diagnosis and prognosis. Crit. Care 2006;**10**(3):R82

4. Delerme S, Ray P. Acute respiratory failure in the elderly: diagnosis and prognosis. Age Ageing 2008;**37**(3):251-57

5. Pandor A, Thokala P, Goodacre S, et al. Pre-hospital non-invasive ventilation for acute respiratory failure: a systematic review and cost-effectiveness evaluation. Health Technol. Assess. 2015;**19**(42):v-vi, 1-102

6. Health Do. Hospital Episode Statistics. Secondary Hospital Episode Statistics 2014. http://www.hesonline.nhs.uk

7. Fisher JD JRCALC, National Ambulance Service Medical Directors, and Association of Ambulance Chief Executives. *UK ambulance services clinical practice guidelines: 2013. Class Professional Publishing.*: Class Professional Publishing, 2013.

8. Non-invasive ventilation in acute respiratory failure. Thorax 2002;**57**(3):192-211

For peer review only

9. Fuller GW, Goodacre S, Keating S, et al. The ACUTE (Ambulance CPAP: Use, Treatment effect and economics) feasibility study: a pilot randomised controlled trial of prehospital CPAP for acute respiratory failure. Pilot and feasibility studies 2018;**4**:86-86

10. O'Driscoll BR, Howard LS, Earis J, et al. BTS guideline for oxygen use in adults in healthcare and emergency settings. Thorax 2017;**72**(Suppl 1):ii1-ii90

11. Herdman M, Gudex C, Lloyd A, et al. Development and preliminary testing of the new five-level version of EQ-5D (EQ-5D-5L). Qual. Life Res. 2011;**20**(10):1727-36

12. O_two CPAP unit. Secondary O_two CPAP unit 2018. http://otwo.com/emergency-cpap/o_two-single-use-cpap/.

13. Health Do. Health Research Authority Secondary Health Research Authority 2019. https://www.hra.nhs.uk/.

14. Kruse RL, Alper BS, Reust C, et al. Intention-to-treat analysis: who is in? Who is out? J. Fam. Pract. 2002;**51**(11):969-71

15. Teare MD, Dimairo M, Shephard N, et al. Sample size requirements to estimate key design parameters from external pilot randomised controlled trials: a simulation study. Trials 2014;**15**:264

For peer review only

16. Arain M, Campbell MJ, Cooper CL, et al. What is a pilot or feasibility study? A review of current practice and editorial policy. BMC Med. Res. Methodol. 2010;**10**:67-67

17. Leon AC, Davis LL, Kraemer HC. The role and interpretation of pilot studies in clinical research. J. Psychiatr. Res. 2011;**45**(5):626-29

18. Thabane L, Ma J, Chu R, et al. A tutorial on pilot studies: the what, why and how. BMC Med. Res. Methodol. 2010;**10**:1-1

19. Bello G, De Pascale G, Antonelli M. Noninvasive Ventilation. Clin. Chest Med. 2016;**37**(4):711-21

20. Hensel M, Strunden MS, Tank S, et al. Prehospital non-invasive ventilation in acute respiratory failure is justified even if the distance to hospital is short. Am. J. Emerg. Med. 2019;**37**(4):651-56

21. Jacob J, Arranz M, Sancho Ramoneda M, et al. [Noninvasive mechanical ventilation in emergency services in Catalonia: the VNICat registry cohort study]. Emergencias : revista de la Sociedad Espanola de Medicina de Emergencias 2017;**29**(1):33-38

22. Luiz T, Kumpch M, Gruttner J, et al. Prehospital CPAP Therapy by Emergency Physicians in Patients with Acute Respiratory Failure due to Acute Cardiogenic Pulmonary Edema or Acutely Exacerbated COPD. In Vivo 2016;**30**(2):133-9

23. Sahu N, Matthews P, Groner K, et al. Observational Study on Safety of Prehospital BLS CPAP in Dyspnea. Prehosp. Disaster Med. 2017;**32**(6):610-14

For peer review only

24. Willmore A, Dionne R, Maloney J, et al. Effectiveness and safety of a prehospital program of continuous positive airway pressure (CPAP) in an urban setting. Cjem 2015;**17**(6):609-16

For peer review only

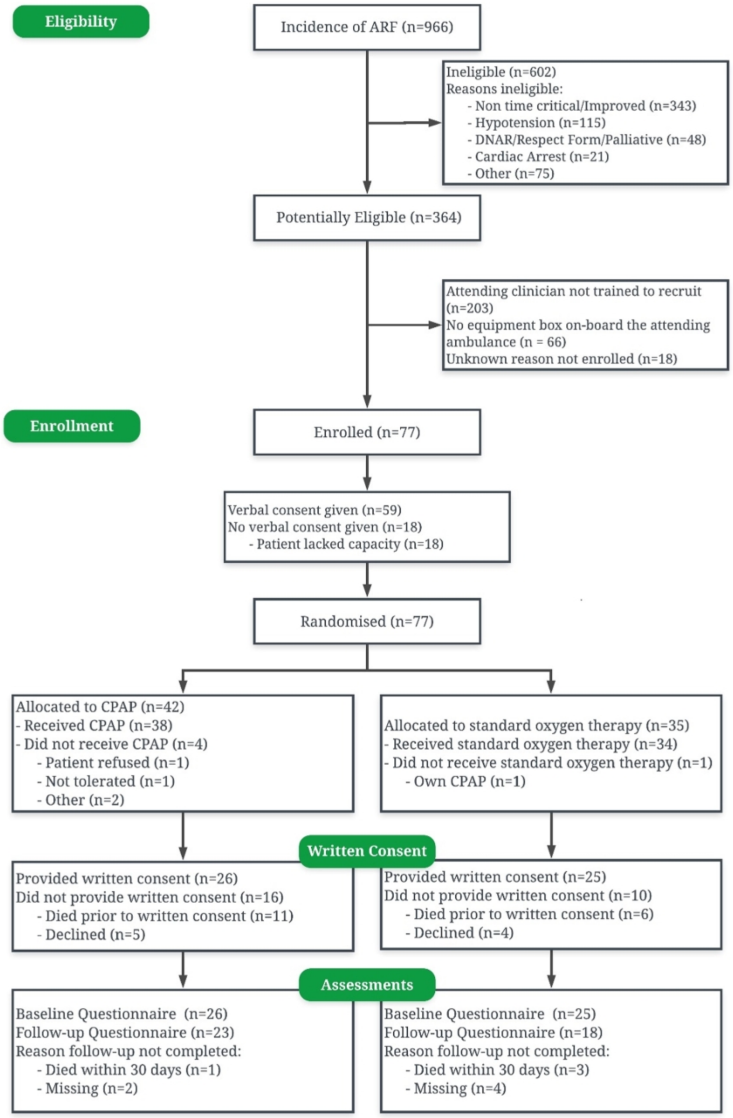

Figure 1. CONSORT diagram of participants' flow through the ACUTE pilot trial

224x313mm (300 x 300 DPI)

ACUTE pilot trial post-discharge healthcare resource use

Table showing post-discharge healthcare resource use up to 30 days after enrolment

		CPAP<br>N = 42	Standard Oxygen Therapy<br>N = 35	Total<br>N = 77
Discharged after index admission	n=	36	30	66
		22 (61.1%)	22 (73.3%)	44 (66.7%)
Readmission for any reason	n=	22	22	44
		5 (22.7%)	6 (27.3%)	11 (25.0%)
Healthcare provider consulted	n=	23	18	41
		14 (60.9%)	14 (77.8%)	28 (68.3%)
Healthcare provider seen	GP	7 (50.0%)	8 (57.1%)	15 (53.6%)
	Practice or district nurse	7 (50.0%)	5 (35.7%)	12 (42.9%)
	Emergency Department	4 (28.6%)	3 (21.4%)	7 (25.0%)
	Physiotherapist (out of hospital)	2 (14.3%)	3 (21.4%)	5 (17.9%)
	Other*	3 (21.4%)	7 (50.0%)	10 (35.7%)

*Other healthcare providers seen were: Oxygen supplier, care workers, community respiratory team, dentist, heart failure nurse, occupational therapy, outpatient appointment, outpatient x-ray, pharmacist, social worker. Some participants saw multiple other healthcare providers.

For peer review only

ACUTE pilot trial safety reporting procedures and adverse events

Flow chart detailing safety reporting procedures for the ACUTE study

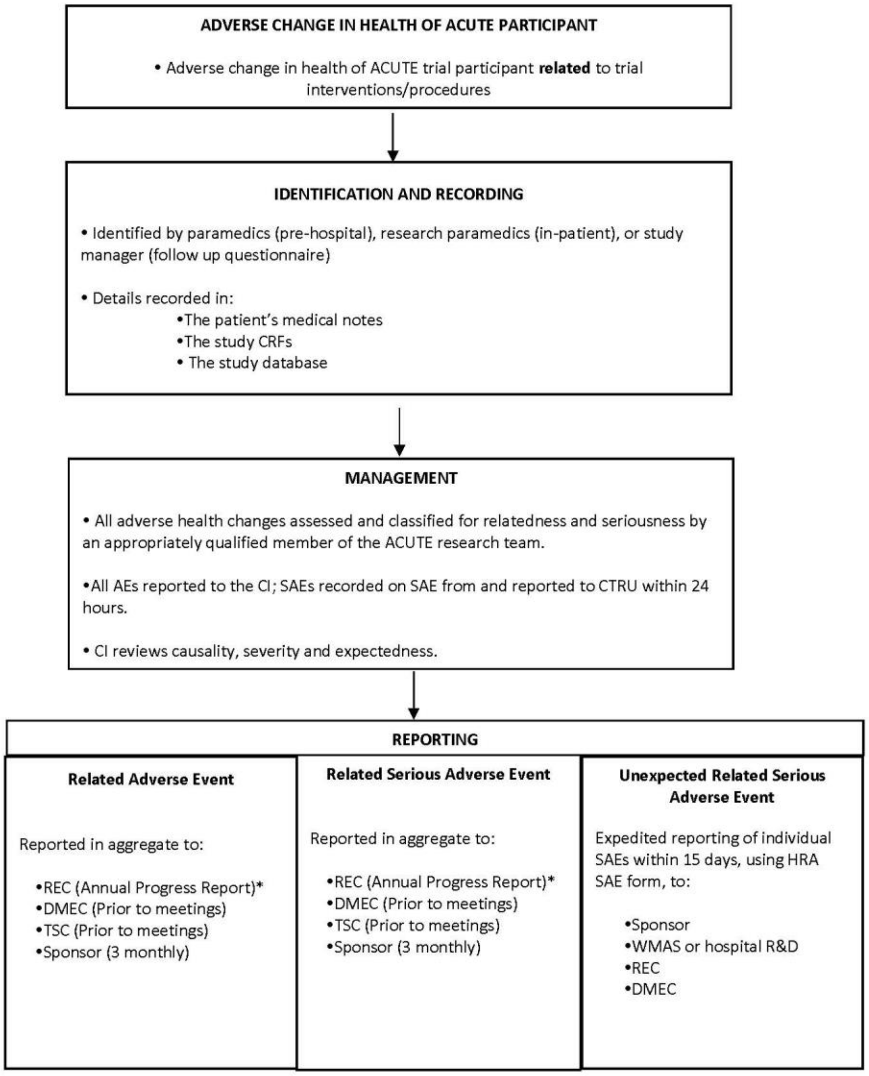

*Related AEs/SAEs resulting in treatment failure

Table of classification of adverse events.

Adverse Health Change Terminology*	Definition
**Seriousness**	
**Adverse Event (AE)**	An adverse change in health that occurs while a patient is taking part in a study
**Serious Adverse Event (SAE)**	Any adverse event occurring while a patient is taking part in a study, that results in:<br><br>• Death<br>• Life Threatening Illness<br>• Hospitalisation<br>• Prolongation of Hospitalisation<br>• Disability or Incapacity<br>• Congenital abnormality or birth defect<br>• Other adverse health change resulting in inability to perform routine activities or significant medical event
**Causality**	
**Unrelated**	An adverse change in health that occurs while a patient is taking part in a study which is not caused by or related to trial treatments.
**Related**	An adverse change in health that occurs while a patient is taking part in a study, which is caused by or related to trial treatments. An AE or SAE is considered related if the relationship between the event and trial treatments is:<br><br>• Possible - There is some evidence to suggest a causal relationship. However, the influence of other factors may have contributed to the event (e.g. the participant's clinical condition, other concomitant treatments).<br>• Probable - There is evidence to suggest a causal relationship and the influence of other factors is unlikely, or<br>• Definite - There is clear evidence to suggest a causal relationship and other possible contributing factors can be ruled out.
**Expectedness**	
**Unexpected**	Any adverse health change that is **NOT** consistent with the known and expected adverse events of trial treatments i.e. it is not listed in the protocol or related documents/literature as an expected occurrence.
**Expected**	Any adverse health change that **IS** consistent with the known and expected adverse events of trial treatments
**Severity***	
**Mild**	An adverse health change that does not interfere with routine activities
**Moderate**	An adverse health change that interferes with routine activities
**Severe**	An adverse health change that makes it impossible to perform routine activities

*The term 'severity', is used to describe the intensity, and should not be confused with 'serious' which is based on participant/event outcome or action criteria. For example, a headache may be severe but not serious, while a minor stroke is serious but may not be severe.

For peer review only

Table summary of the number of AEs and SAEs as well as the proportion of patients experiencing at least one AE or SAE for the ITT population

	CPAP	Standard Oxygen Therapy	Total
Patients	42	35	77
Number of AEs	27	20	47
Number of individuals with an AE	23 (54.8%)	17 (48.6%)	40 (51.9%)
Number of SAEs	22	17	39
Number of individuals with a SAE	19 (45.2%)	15 (42.9%)	34 (44.2%)

ACUTE pilot trial adverse events

Site	AE summary	AE category	Start date	End date	Serious?	Expected SAE?
Stoke	Headache	Other	19/08/2017	19/08/2017	No	No
Stoke	Arrested prior to receiving intervention	Cardiorespiratory arrest	13/09/2017	13/09/2017	Yes	No
Lichfield	Respiratory arrest	Other	17/09/2017	17/09/2017	Yes	No
Lichfield	Died prior to approach	Cardiorespiratory arrest	18/09/2017	18/09/2017	Yes	No
Stoke	Fractured NOF	Other	18/10/2017		Yes	No
Erdington	Social Care readmission	Other	23/10/2017	08/12/2017	Yes	No
Lichfield	CVA	Other	28/10/2017	15/12/2017	Yes	No
Stafford	Death	Cardiorespiratory arrest	29/10/2017	29/10/2017	Yes	Yes
Stafford	Death	Cardiorespiratory arrest	29/10/2017	29/10/2017	Yes	Yes
Stoke	Pt died	Cardiorespiratory arrest	16/11/2017	16/11/2017	Yes	No
Stoke	Re-admission within 30 days	Other	30/11/2017	30/11/2017	No	Yes
Stoke	Claustrophobia	Claustrophobia	12/12/2017	12/12/2017	No	Yes
Erdington	Distress at mask	Other	15/12/2017	15/12/2017	No	Yes

For peer review only

Erdington	Died within 30 days	Cardiorespiratory arrest	21/12/2017	21/12/2017	Yes	No
Stafford	Pt died	Cardiorespiratory arrest	29/12/2017	29/12/2017	Yes	Yes
Stoke	Mild claustrophobia	Claustrophobia	05/01/2018	05/01/2018	No	Yes
Erdington	Readmission to respiratory ward with pneumonia	Other	05/01/2018	15/01/2018	Yes	Yes
Stafford	Re-admission	Other	10/01/2018		Yes	No
Stoke	Death	Cardiorespiratory arrest	14/01/2018	14/01/2018	Yes	Yes
Stoke	Pt died	Cardiorespiratory arrest	17/01/2018	17/01/2018	Yes	Yes
Stafford	Pt died on day of recruitment	Cardiorespiratory arrest	23/01/2018	23/01/2018	Yes	Yes
Stafford	Died ahead of 30 day follow up	Cardiorespiratory arrest	27/01/2018	27/01/2018	Yes	Yes
Stafford	Died on elderly care ward	Cardiorespiratory arrest	28/01/2018	28/01/2018	Yes	Yes
Stoke	Agitation and distress with mask	Progressive respiratory distress	28/01/2018	28/01/2018	No	Yes
Erdington	Death	Cardiorespiratory arrest	06/02/2018	06/02/2018	Yes	Yes
Stafford	Patient died	Cardiorespiratory arrest	20/02/2018	20/02/2018	Yes	Yes
Lichfield	Died prior to 30 day	Cardiorespiratory arrest	03/03/2018	03/03/2018	Yes	Yes
Stafford	Pt re-admitted to hospital during 30-day period after recruitment	Other	08/03/2018	15/03/2018	Yes	Yes
Lichfield	Died prior to approach	Cardiorespiratory arrest	09/03/2018	09/03/2018	Yes	Yes
Erdington	Died following discharge prior to approach	Cardiorespiratory arrest	10/03/2018	10/03/2018	Yes	Yes
Lichfield	Died prior to approach	Cardiorespiratory arrest	17/03/2018	17/03/2018	Yes	Yes

For peer review only

Stoke	Pneumothorax	Tension pneumothorax	28/03/2018	28/03/2018	Yes	Yes
Stoke	Patient got worse	Vomiting	28/03/2018	28/03/2018	No	Yes
Stafford	Pt re-admitted, then died	Other	12/04/2018	15/04/2018	Yes	Yes
Lichfield	Mask wouldn't inflate	Other	17/04/2018	17/04/2018	No	No
Lichfield	Cardiac arrest	Cardiorespiratory arrest	08/05/2018	08/05/2018	Yes	Yes
Lichfield	Death	Cardiorespiratory arrest	31/05/2018	31/05/2018	Yes	Yes
Stafford	Died prior to approach	Cardiorespiratory arrest	03/06/2018	03/06/2018	Yes	Yes
Lichfield	Readmissions within 30 days	Other	30/06/2018	01/07/2018	Yes	Yes
Lichfield	Readmission within 30 days	Other	02/07/2018	06/07/2018	Yes	Yes
Lichfield	Claustrophobia and Progressive Respiratory Distress	Other	05/07/2018	05/07/2018	No	Yes
Lichfield	Pneumothorax	Other	05/07/2018	27/07/2018	Yes	Yes
Stafford	Re-admission	Other	06/07/2018		Yes	Yes
Lichfield	Readmission	Other	07/07/2018	27/07/2018	Yes	Yes
Stoke	Pt re-admitted during 30-day reference period	Other	25/07/2018	03/08/2018	Yes	Yes
Lichfield	Readmission	Other	28/07/2018		Yes	Yes
Stoke	Death	Cardiorespiratory arrest	22/08/2018	22/08/2018	Yes	Yes

For peer review only

CONSORT 2010 checklist of information to include when reporting a pilot or feasibility randomized trial in a journal or conference abstract

Item	Description	Reported on line number
Title	Identification of study as randomised pilot or feasibility trial	Title page
Authors *	Contact details for the corresponding author	Title page
Trial design	Description of pilot trial design (eg, parallel, cluster)	7
Methods		
Participants	Eligibility criteria for participants and the settings where the pilot trial was conducted	7
Interventions	Interventions intended for each group	11
Objective	Specific objectives of the pilot trial	6
Outcome	Prespecified assessment or measurement to address the pilot trial objectives**	8
Randomization	How participants were allocated to interventions	7,10
Blinding (masking)	Whether or not participants, care givers, and those assessing the outcomes were blinded to group assignment	10
Results		
Numbers randomized	Number of participants screened and randomised to each group for the pilot trial objectives**	15
Recruitment	Trial status†	N/A
Numbers analysed	Number of participants analysed in each group for the pilot objectives**	14,15
Outcome	Results for the pilot objectives, including any expressions of uncertainty**	14-21
Harms	Important adverse events or side effects	20
Conclusions	General interpretation of the results of pilot trial and their implications for the future definitive trial	24,25
Trial registration	Registration number for pilot trial and name of trial register	7
Funding	Source of funding for pilot trial	12

Citation: Eldridge SM, Chan CL, Campbell MJ, Bond CM, Hopewell S, Thabane L, et al. CONSORT 2010 statement: extension to randomised pilot and feasibility trials. BMJ. 2016;355.

**this item is specific to conference abstracts*

***Space permitting, list all pilot trial objectives and give the results for each. Otherwise, report those that are a priori agreed as the most important to the decision to proceed with the future definitive RCT.*

†For conference abstracts.

For peer review only
